# Speed it up: How temperature drives toxicokinetics of organic contaminants in freshwater amphipods

**DOI:** 10.1111/gcb.16542

**Published:** 2022-12-09

**Authors:** Johannes Raths, Vid Švara, Benedikt Lauper, Qiuguo Fu, Juliane Hollender

**Affiliations:** ^1^ Department of Environmental Chemistry Swiss Federal Institute of Aquatic Science and Technology – Eawag Dübendorf Switzerland; ^2^ Institute of Biogeochemistry and Pollutant Dynamics, ETH Zürich Zürich Switzerland; ^3^ UNESCO Chair on Sustainable Management of Conservation Areas, Engineering & IT Carinthia University of Applied Sciences Villach Austria; ^4^ Department of Effect‐Directed Analysis Helmholtz Centre for Environmental Research – UFZ Leipzig Germany

**Keywords:** aquatic invertebrates, Arrhenius, bioconcentration, biotransformation, *Gammarus pulex*, *Hyalella azteca*, micropollutants

## Abstract

The acceleration of global climate change draws increasing attention towards interactive effects of temperature and organic contaminants. Many studies reported a higher sensitivity of aquatic invertebrates towards contaminant exposure with increasing or fluctuating temperatures. The hypothesis of this study was that the higher sensitivity of invertebrates is associated with the changes of toxicokinetic processes that determine internal concentrations of contaminants and consequently toxic effects. Therefore, the influence of temperature on toxicokinetic processes and the underlying mechanisms were studied in two key amphipod species (*Gammarus pulex* and *Hyalella azteca*). Bioconcentration experiments were carried out at four different temperatures with a mixture of 12 exposure relevant polar organic contaminants. Tissue and medium samples were taken in regular intervals and analysed by online solid‐phase extraction liquid chromatography high‐resolution tandem mass spectrometry. Subsequently, toxicokinetic rates were modelled and analysed in dependence of the exposure temperature using the Arrhenius equation. An exponential relationship between toxicokinetic rates versus temperature was observed and could be well depicted by applying the Arrhenius equation. Due to a similar Arrhenius temperature of uptake and elimination rates, the bioconcentration factors of the contaminants were generally constant across the temperature range. Furthermore, the Arrhenius temperature of the toxicokinetic rates and respiration was mostly similar. However, in some cases (citalopram, cyprodinil), the bioconcentration factor appeared to be temperature dependent, which could potentially be explained by the influence of temperature on active uptake mechanisms or biotransformation. The observed temperature effects on toxicokinetics may be particularly relevant in non‐equilibrated systems, such as exposure peaks in summer as exemplified by the exposure modelling of a field measured pesticide peak where the internal concentrations increased by up to fourfold along the temperature gradient. The results provide novel insights into the mechanisms of chemical uptake, biotransformation and elimination in different climate scenarios and can improve environmental risk assessment.

## Graphical abstract

The acceleration of global climate change draws increasing attention towards interactive effects of temperature and organic contaminants. An exponential relationship between toxicokinetic rates in aquatic invertebrates versus temperature was observed and could be well depicted by applying the Arrhenius equation. Our results provide novel insights into the mechanisms of chemical uptake, biotransformation and elimination in different climate scenarios and can support risk assessment implementations.

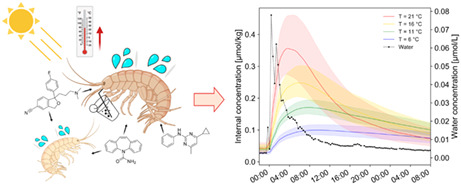

## 1 INTRODUCTION

### 1.1 Threats towards aquatic biodiversity

Environmental pollution and climate change are two major threats to ecosystem integrity and biodiversity (IPBES, 2019; IPCC, 2021). Increased pollution and higher temperatures worldwide already resulted in a 83% decrease of the Freshwater Living Planet Index since 1970 (Grooten et al., 2018). While environmental parameters and exposure profiles in the field are highly fluctuating on both the temporal and spatial scale, laboratory experiments for environmental risk assessment are highly standardized. This is especially the case when it comes to studies required in the registration processes such as the REACH legislation (EC, 2006) or regulation of plant protection products in the European Union (EC, 2002). Retrospective risk assessment, such as monitoring studies of freshwater ecosystems, often reveals higher contaminant exposure risk (i.e. internal contaminant concentrations) of biota than expected based on extrapolation from laboratory data (Lauper et al., 2021; Munz et al., 2018). Furthermore, effects of multiple stressors such as environmental parameters (i.e. temperature), inter‐ and intraspecies interactions as well as contaminant mixture effects have been demonstrated to increase the adverse effect of organic contaminants on biota in the field (Holmstrup et al., 2010; Hooper et al., 2013).

### 1.2 Interaction of climate and pollution

The observed interaction between organic contaminants and increasing temperature scenarios towards ectothermic organisms, such as aquatic invertebrates, is gaining more attention recently. This is also due to the fact that increased water concentrations of pesticides generally coincide with higher water temperatures in the application season (Arlos et al., 2020; Chow et al., 2020; Lauper et al., 2021; Munz et al., 2017; Phillips & Bode, 2004). More severe effects of organic contaminants on freshwater organisms were observed at higher temperatures, increasing daily temperature fluctuations (DTF) and less heat adapted populations (Theys et al., 2020; Verheyen et al., 2022; Verheyen & Stoks, 2019). However, the mechanistic understanding of this interaction is limited (Polazzo et al., 2022). For instance, it remains unclear to which extent these observations are related to toxicokinetic (determines internal concentration of contaminants) rather than toxicodynamic (determines damage caused by the internal contaminant concentration) processes. Important toxicokinetic parameters are uptake, elimination and biotransformation rates as well as the bioconcentration factor (BCF, ratio of internal and exposure concentration under equilibrium conditions). Systematic investigations on the impact of temperature on toxicokinetics, such as determination of toxicokinetic rates or assuring equilibrium conditions, are rare (Dai et al., 2021; Mangold‐Döring et al., 2022). Furthermore, contrasting results such as higher (Buchwalter et al., 2003; Camp & Buchwalter, 2016; Dai et al., 2021; Nawaz & Kirk, 1996) (caddisfly, stonefly, mayfly, earthworm, daphnids), indifferent (Cerveny et al., 2021; Kuo & Chen, 2021) (fish, midge) or lower (Brown et al., 2021; Muijs & Jonker, 2009) (frog, aquatic worm) internal contaminant concentrations at higher temperatures are reported in ectothermic aquatic organisms.

### 1.3 Amphipods in risk assessment

The present study chooses the two aquatic amphipod species *Gammarus pulex* (Linnaeus, 1758) and *Hyalella azteca* (Saussure, 1858) as model organisms. Both species are common shredders of benthic communities, can be highly abundant and are a key link in trophic transfer from lower to higher trophic levels. However, they are geographically widespread in different continents—*G. pulex* in Europe and Asia (Graça et al., 1994) and *H. azteca* in Central and North America (US EPA, 2000). *G. pulex* is more sensitive towards changes in environmental parameters (i.e. temperature, oxygen, salinity) than *H. azteca* (Cottin et al., 2012; Javidmehr et al., 2015; Maltby, 1995). Amphipods are well established for laboratory studies (McCahon & Pascoe, 1988; US EPA, 2000) but are recently also used for retrospective monitoring approaches (Berlioz‐Barbier et al., 2014; Lauper et al., 2021; Miller et al., 2015; Munz et al., 2018). In line with the 3R principle of animal testing (de Wolf et al., 2007; Russell & Burch, 1959), amphipods are discussed as an alternative test system to bioaccumulation studies with fish according to OECD 305 (Kosfeld et al., 2020; OECD, 2012; Schlechtriem et al., 2019). However, when comparing responses of the two species towards chemical exposure, not only the species differences but also differences in the natural habitats or experimental test parameters (i.e. temperature) are integrated. Thus, potential observed species differences could as well be an artefact of different test parameters. Furthermore, both monitoring and laboratory data are difficult to transfer globally and to different climate regimes or future scenarios if the impact of temperature is not considered.

### 1.4 Arrhenius theory

One approach to describe the temperature dependence of reaction rates is the Arrhenius equation (Laidler, 1984). The classic Arrhenius describes an exponential decrease of a chemical reaction rate with inverse temperature. It is applied under the assumption of a single, rate‐limiting, thermally activated process and an activation energy independent of temperature. This approach is widely used to temperature correct (bio‐)chemical processes such as degradation and membrane passage (EFSA, 2008; Filippov et al., 2003; Meynet et al., 2020) but is also used to describe physiological processes such as oxygen consumption (standard metabolic rate) (Arroyo et al., 2022; Brown et al., 2004). Furthermore, recent studies successfully applied the Arrhenius equation to evaluate thermal stress (Jørgensen et al., 2021) or temperature correct toxicity data (Gergs et al., 2019) of different species test systems.

### 1.5 Research objectives

In the present study, we aimed to systematically elucidate how temperature affects uptake, elimination and biotransformation rates, as well as bioconcentration factors in the two amphipod species in order to account for changing climatic conditions. Based on the Arrhenius theory, we expected an increase of toxicokinetic rates with temperature despite contradicting results (increasing, indifferent, decreasing) on the internal concentration in previous studies. To test this hypothesis, we studied a selection of 12 in surface waters frequently detected polar organic contaminants with different properties, such as differences in ion speciation and biotransformation capability. The obtained toxicokinetic rates were applied to different environmental temperature and exposure scenarios in order to evaluate the change of toxicokinetics under different climate scenarios. Eventually, the underlying mechanisms of temperature‐dependent toxicokinetics are discussed and recommendations for Arrhenius theory‐based implementations of temperature in environmental risk assessment are provided.

## 2 MATERIALS AND METHODS

### 2.1 Test animals

Specimens of *G. pulex* were collected in September 2020 from an uncontaminated creek near Zurich (Mönchaltdorfer Aa, 47.2749°N, 8.7892°E), located in a landscape conservation area. The water temperature at the time of sampling was 17°C. Adult specimens of *H. azteca* were taken from the in‐house laboratory culture (19 ± 1°C) of the Department of Environmental Chemistry, Eawag (Dübendorf, Switzerland) originally obtained from the laboratory of Fraunhofer IME (Schmallenberg, Germany). All animals used in the performed experiments were acclimated to the test conditions (tank, medium temperature, light condition, population density) for 4 days prior to the experiments. Further details on the test medium and organisms are provided in SI A1. Specimens of the *H. azteca* culture belonged to a clade originating from Florida. Specimens of *G. pulex* belonged to a clade distributed north of the Alps in eastern France, Switzerland and to Regensburg in Germany. Genetic specifications of the organisms were performed according to Švara et al. (2019). More details on the genetic specification are provided in SI A2.

### 2.2 Standard metabolic rates

In order to evaluate to what extent temperature‐related changes in toxicokinetics in the tested amphipods could be explained by physiological temperature responses, temperature‐dependent respiration was measured as a physiological endpoint. Respirometry experiments were conducted with *H. azteca* at four different temperatures (6, 11, 16 and 21°C) using a 10‐channel respirometer equipped with fibre‐optic oxygen mini sensors (FIBOX 3, PreSens). The oxygen consumption was measured in eight replicate chambers containing one specimen of *H. azteca* and two control chambers. Standard metabolic rates (in μg O_2_ g^−1^ h^−1^, dry weight *dw* basis) were determined and temperature relationships analysed along the lines of toxicokinetic rates. Details on the respiration experiments are provided in SI A8.

### 2.3 Test compounds

The exposure mixture of 12 polar compounds (including four ionic compounds) was chosen from organic contaminants that are regularly found in surface water monitoring studies as well as compounds with a mismatch (underestimation, i.e. azoxystrobin, citalopram, cyprodinil, fluopyram, thiacloprid) of predicted and measured internal concentrations in gammarids from Swiss rivers (Arlos et al., 2020; Lauper et al., 2021; Munz et al., 2018). The selection also intentionally contained compounds with identified biotransformation products in amphipods (Fu et al., 2018, 2020; Jeon et al., 2013; Rösch et al., 2016). The composition included six pesticides (azoxystrobin (AZ), cyprodinil (CY), fluopyram (FLU), tebuconazole (TEB), terbutryn (TER), thiacloprid (THI)), five pharmaceuticals (atenolol (AT), carbamazepine (CMZ), citalopram (CIT), diclofenac (DCF), sulfamethoxazole (SFX)) and one industrial compound (benzotriazole (BTX)). The selected compounds covered a log *D*_*ow*_ (octanol–water partitioning coefficient at pH 7.9) range from −1.3 to 4.0.

We assumed that the toxicokinetics of the compounds in the mixture did not interact (i.e. CYP‐450 inhibition by azole fungicides, Rösch et al., 2017) at the tested concentration (50 μg L^−1^). Furthermore, the compounds had different molecular targets (Table S1) and no known interference with each other. Despite similar enzymes involved in biotransformation (i.e. CYP‐450) of multiple compounds, the concentrations were not assumed to cause saturation effects. An overview of the test compounds and their properties is provided in SI A3.

### 2.4 Bioconcentration experiments

**FIGURE 1:**
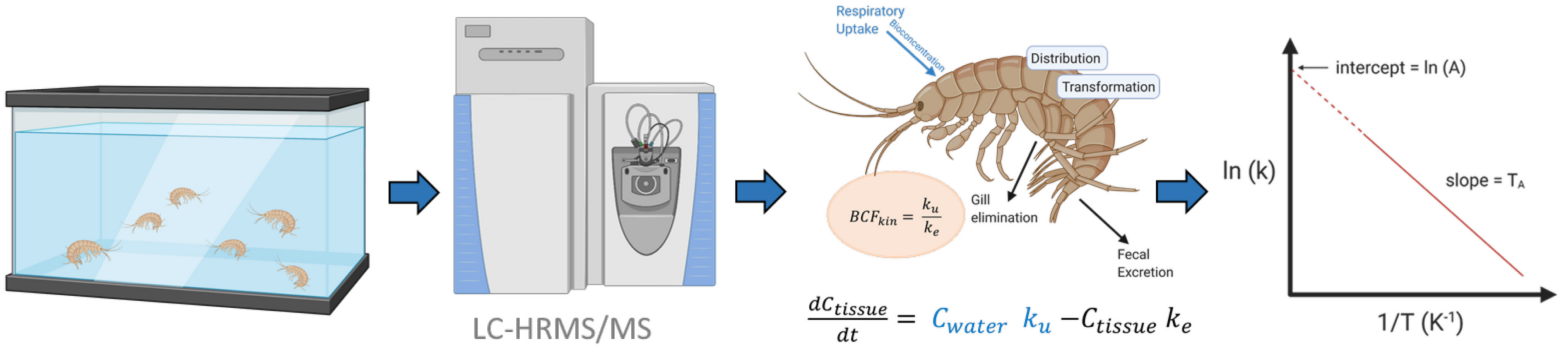
Simplified workflow of the bioconcentration experiments and subsequent modelling. From left to right: Uptake–elimination experiments, determination of internal concentrations using LC‐HRMS/MS, toxicokinetic modelling using the BYOM platform and application of the Arrhenius equation.

A simplified workflow of the bioconcentration experiments and subsequent modelling is presented in Figure 1.

For each of the two species, one bioconcentration experiment was performed at each of four different temperatures (6, 11, 16 and 21°C). The temperatures represent the range gammarids experience at their collection site (Baumgartner & Robinson, 2015) or in other streams during monitoring studies (Lauper et al., 2021). Furthermore, the range included the temperatures at which the two species are usually tested in laboratory experiments, which is 23 ± 2°C for *H. azteca* (Schlechtriem et al., 2019; US EPA, 2000) and 11–16°C for *G. pulex* (Ashauer et al., 2012; Fu et al., 2018; Jeon et al., 2013; Miller et al., 2016). One additional experiment at 11°C was set up with heat shock euthanized gammarids (few seconds in 55°C tap water) to investigate the impact of physiological activity (filtration, biotransformation) on toxicokinetics. Each toxicokinetic experiment consisted of a 1‐day uptake phase followed by up to 3 days of elimination. During the uptake phase, the test population was exposed to an exposure medium containing 50 μg L^−1^ of each of the 12 test compounds in order to achieve sufficient internal concentrations for parent and biotransformation products (BTPs) analysis but keep toxic effects minimized (most LC_50_s > mg/L, see SI A3). No food was provided during the uptake phase to exclude additional uptake from the diet. For the elimination phase, remaining organisms were transferred into uncontaminated test medium containing leaf discs (*G. pulex*) or ground fish food flakes (*H. azteca*). The experiments were performed under static conditions in glass tanks containing 6 L of test medium (SI A1) and a population density of 34 and 126 individuals per litre of *G. pulex* and *H. azteca* respectively. All experiments were conducted at the same time in four different climate chambers (one for each temperature) and a 16‐h/8‐h light/dark cycle. Medium and animal samples (4 gammarids or 15 hyalella) were taken as duplicates in regular intervals over the period of the experiments. Animals that died during the test were excluded. Preliminary experiments with *G. pulex* with a reduced temperature range and reduced sampling rate are described in SI A9.

### 2.5 Sample preparation

Samples were collected and extracted by liquid extraction as introduced elsewhere (Rösch et al., 2016). In brief, sampled animals were rinsed with nanopure water (NPW), dry blotted on tissue paper, transferred into 2 ml centrifuge vials, weighed (wet weight (ww), conversion factors to dry weight are compiled in SI A4) and frozen in liquid nitrogen. For sample extraction, 300 mg of 1 mm zirconia/silica beads (BioSpec Products, Inc.), 100 μl of isotope labelled internal standard mixture (250 μg L^−1^ deuterated reference standards, Table S7) in methanol and 500 μl of pure methanol were added before samples were homogenized using a FastPrep bead beater (two cycles of 15 s at 6 m s^−1^; MP Biomedicals). Afterwards, samples were centrifuged (10,000 *g* × 6 min, 4°C). The solvent was collected with syringes and filtered through 0.45 μm regenerated cellulose filters. The filters were washed with another 400 μl of pure methanol and the two filtrates combined.

Medium samples (500 μl) were collected from the tanks, spiked with 100 μl of internal standard mixture in methanol and mixed with another 400 μl of pure methanol. All samples were stored at −20 °C until chemical analysis.

### 2.6 Chemical analysis

Chemical analysis was performed using an automated online solid‐phase extraction system coupled with a reversed phase liquid chromatography and high‐resolution tandem mass spectrometer (online‐SPE‐LC‐HRMS/MS; Q Exactive, Thermo Fisher Scientific Inc.). An electrospray ionization interface was used for ionization. Full scan acquisition was performed with a resolution of 70,000 (at m/z 200) in polarity switching mode followed by data‐dependent MS/MS scans (five scans at positive mode and two at negative mode) with a resolution of 17,500 (at m/z 200) and an isolation window of 1 m/z. Detailed information on the test system, quality control and quantification are provided in SI A5.

A suspect screening on BTPs was based on a list of previously identified and reported BTPs in amphipods or other animals and plants (Table S9). BTPs were screened using the acquired HRMS/MS raw data requiring their unique presence in the treatment and absence in all controls. If available, BTPs were quantified using a reference standard. Other BTPs were semi‐quantified based on the calibration curve of the parent compound. BTPs of terbutryn were semi‐quantified based on the calibration of irgarol‐descyclopropyl (TER_M214), due to a similar retention time and the higher ionization efficiency of the BTPs (Jeon et al., 2013; Kosfeld et al., 2020). Quantification was only performed for compounds with a peak area ≥5% of the parent compound at 24 h of exposure (SI A5).

### 2.7 Determination of lipid and protein content

Samples for lipid and protein content analysis were collected as described for the chemical analysis. Lipid content was determined gravimetrically (Smedes, 1999) following an adapted protocol based on Raths et al. (2020). Total protein content was determined using the Pierce BCA Protein Assay Kit (ThermoScientific) with bicinchoninic acid (Janssen et al., 2012). Details on the methods are provided in SI A6.

### 2.8 Toxicokinetic modelling

For the determination of uptake and elimination rates and kinetic bioconcentration factors (BCF_kin_), a one‐compartment first‐order model (‘parent model’) was applied. The model was implemented in the Matlab (R2019b)‐based scripts of the ‘Acute *Calanus* package’ version 1.1 (Jager et al., 2017) of the Build Your Own Model (BYOM) platform (https://www.debtox.info/byom.html). The parent tissue concentration *C*_tissue*,p*_ (μmol kg_ww_^−1^) in the organisms over time was described by the following ordinary differential equation:

(1) $$\frac{dC_{\text{tissue},p}\left(t\right)}{\mathrm{dt}}=C_{\text{water}}\left(t\right)∙k_{u}-C_{\text{tissue},p}\left(t\right)∙k_{e}$$

where *C*_water_ is the average medium concentration (μmol L^−1^), the uptake rate *k*_u_ (L kg_ww_^−1^ day^−1^) describes dermal and respiratory uptake and the elimination rate *k*_e_ (day^−1^) integrates the elimination of the parent compound by active and passive excretion as well as biotransformation.

For the compounds azoxystrobin, citalopram, cyprodinil, diclofenac, tebuconazole and terbutryn, additional models with an implementation of biotransformation (‘biotransformation model’) as independent elimination process were fitted. To reduce modelled parameters and uncertainties, biotransformation pathways were simplified by grouping BTPs into total primary *C*_tissue,m,1st_ and secondary *C*_tissue,m,2nd_ BTP tissue concentrations (Fu et al., 2018) with the corresponding parent compound or BTP as precursor respectively. Thereby the total elimination of the parent *k*_*e*_ is separated into an elimination rate for excretion *k*_e,p_ (day^−1^) and a rate for primary biotransformation *k*_m,1st_ (day^−1^). Secondary biotransformation is described analogously using *k*_m,2nd_ (day^−1^). Furthermore, elimination rates for the primary *k*_e,1st_ (day^−1^) and secondary *k*_e,2nd_ (day^−1^) BTPs are introduced. The first‐order ordinary differential equations employed in the model are described as follows:

Parent compound:

(2) $$\frac{dC_{\text{tissue},p}\left(t\right)}{\mathrm{dt}}=C_{\text{water}}\left(t\right)∙k_{u}-C_{\text{tissue},p}\left(t\right)∙k_{e,p}-C_{\text{tissue},p}\left(t\right)∙k_{m,1\mathrm{st}}$$

Primary BTPs:

(3) $$\frac{dC_{\text{tissue},m,1\mathrm{st}}\left(t\right)}{\mathrm{dt}}=C_{\text{tissue},p}\left(t\right)∙k_{m,1\mathrm{st}}-C_{\text{tissue},m,1\mathrm{st}}\left(t\right)∙k_{e,1\mathrm{st}}-C_{\text{tissue},m,1\mathrm{st}}\left(t\right)∙k_{m,2\mathrm{nd}}$$

Secondary BTPs:

(4) $$\frac{dC_{\text{tissue},m,2\mathrm{nd}}\left(t\right)}{\mathrm{dt}}=C_{\text{tissue},m,1\mathrm{st}}\left(t\right)∙k_{m,2\mathrm{nd}}-C_{\text{tissue},m,2\mathrm{nd}}\left(t\right)∙k_{e,2\mathrm{nd}}$$

Kinetic BCFs (BCF_kin_) were calculated based on the kinetic rates:

(5) $${\mathrm{BCF}}_{\mathrm{kin}}=\frac{k_{u}}{k_{e}}\;\text{or}\;\frac{k_{u}}{k_{e,p}+k_{m,1\mathrm{st}}}\;$$

Additionally, apparent bioconcentration factors (Arnot & Gobas, 2006) after 24 h of exposure (BCF_24h_, L kg_ww_^−1^) were calculated as the ratio between the experimental determined average concentration of the parent compound in the test medium and the internal concentration after 24 h (*C*_tissue*,p,* (24h)_, μmol kg_ww_^−1^):

(6) $${\mathrm{BCF}}_{24h}=\frac{C_{\text{tissue},p,\left(24h\right)}\;}{C_{\text{water}}}$$

All model parameters were fitted simultaneously to the measured internal concentrations using the analytical solution according to Jager and Ashauer (2018). Data sets were weighted by the number of animals per replicate. During the uptake phase, the average measured medium concentration was used and medium concentrations were set to zero during the elimination phase, which was confirmed by the chemical analysis. Best‐fit parameters and 95% confidence intervals (CIs), using profile likelihoods, were used for further data processing.

If the biotransformation models generated various outputs of comparable Akaike information criterions (AIC, difference less than 2), the final model solution was selected in favour of a higher R^2^ for the parent and primary BTP, rather than the secondary BTP. The decision was based on the fact that the secondary BTP concentrations were determined with higher uncertainty. Information on the calculation of elimination half‐life times *t*_1/2_ and time to reach 95% of the steady‐state *t*_*ss*_ (equilibrium condition) are provided in SI A7.

### 2.9 Arrhenius equation

In order to determine the dependence of toxicokinetic rates and standard metabolic rates on temperature, the Arrhenius equation was applied, which assumes an exponential relationship between temperature and the reaction rates. The natural logarithm of the modelled rates (ln *k*) was plotted against the inverse temperature (T^−1^ in K^−1^) and the Arrhenius temperature *T*_A_ (K) was derived from the slope of a linear regression using the following equation:

(7) $$\ln\;k=-T_{A}\;\frac{1}{T}+\ln\;A$$

where *A* is the frequency factor and intercept with the axis of ordinate.

The linear regression fits were calculated in GraphPad Prism 9.4.0 (GraphPad Software, Inc.). Arrhenius temperatures were compared between the different experimentally determined toxicokinetic and physiological rates in the present study, as well as physiological *T*_A_ estimates obtained from the Add‐my‐Pet database (AmP, 2018) for *G. pulex* (*T*_A_ = 10,560 K) and *H. azteca* (*T*_A_ = 10,830 K). The temperature dependency of the BCF_kin_ was assessed by using a linear regression model. If the slope of the fit was significantly different from zero (*p* < .05), a temperature dependency of the BCF_kin_ was concluded.

### 2.10 Model simulations

Two different exposure scenarios, which were a short‐term exposure peak (i.e. due to pesticides mobilized from surface run‐off) and daily temperature fluctuations (DTF) at a constant exposure (i.e. wastewater treatment plant outflow) were modelled in order to compare the impact of temperature on the internal concentrations of amphipods. For this purpose, the python‐based script established and described by Lauper et al. (2021) was fed with the Arrhenius' parameters (parent model) determined in the present study. The differential equation (Equation 1) was solved numerically using Heun's method (Ascher & Petzold, 1998). At each iterative time point, the water temperature was interpolated linearly from the given data points and toxicokinetic rates were subsequently calculated using the Arrhenius equation (Equation 7).

The short‐term exposure peak scenario used a realistic surface water concentration profile (fluopyram and cyprodinil) based on monitoring data in Lauper et al. (2021) with high temporal resolution. The tissue concentrations of *G. pulex* and *H. azteca* were modelled at the four tested temperatures for the same exposure event.

In the DTF scenario, a constant exposure concentration of 50 μg L^−1^ (carbamazepine in *H. azteca*) was chosen and modelled with both a constant temperature (16°C) as well as a daily fluctuating temperature profile (adapted from Verheyen & Stoks, 2019), which had the same average temperature, but ranged over 10°C from 11 to 21°C. The temperature dependence of the toxicokinetic rates (*k*) was implemented using the Arrhenius relationship:

(8) $$k\left(T\right)=A∙e^{-\frac{T_{A}}{T}}$$

## 3 RESULTS

### 3.1 Temperature effects on the standard metabolic rate

The measured temperatures during the respirometry experiments were 7.3, 11.6, 16.4 and 21.2°C (SD = 0.1°C). The standard metabolic rate of *H. azteca* increased exponentially with temperature from 330 ± 80 to 1400 ± 400 (μg O_2_ g^−1^ h^−1^ dw; Figure S5). The standard metabolic rates were very similar to data for adult *H. azteca* generated earlier (Mathias, 1971) as well as the ones reported for mayfly larvae (Camp & Buchwalter, 2016) (Figure S6). The Arrhenius temperature (*T*_A_) calculated from the present data was 8030 ± 1580 K compared to 9070 ± 710 K based on Mathias (1971) (Figure S7). The present *T*_*A*_ was also in range of estimates provided by the Add‐my‐Pet database for *G. pulex* (*T*_A_ = 10,560 K) and *H. azteca* (*T*_A_ = 10,830 K). Based on the very similar reported values for both species, the experimentally determined physiological *T*_*A*_ for *H. azteca* was compared to the *T*_A_s of the toxicokinetic data sets of both amphipod species. A similar *T*_*A*_ of physiological and toxicokinetic rates could indicate that both processes were affected by temperature at the same magnitude.

### 3.2 Measured concentrations and bioconcentration experiment test parameters

The measured temperatures during the bioconcentration experiments were 6.1, 11.0, 15.4 and 21.2°C (SD: 0.2, 0.3, 0.1 and 0.1°C). Measured temperatures are used in all calculations, but for simplification, nominal temperatures are used for nomenclatures. Measured medium concentrations during the uptake phase differed less than 20% from the nominal concentration, except for the medium of *G. pulex* at 21°C with about 20% (and 30% for cyprodinil) lower concentrations at the end of the uptake phase. The measured medium and tissue concentrations are provided in SI B1 to B3. Oxygen saturation was between 80% and 100%. The pH of the test media was 7.9 ± 0.1 and 8.4 ± 0.1 for *G. pulex* and *H. azteca*, respectively. The observed mortality was increasing with temperature from 2, 5, 6 to 12% for *H. azteca* and 14, 10, 21 to 28% for *G. pulex*. The increase of mortality with temperature was similar to that observed elsewhere (Verheyen & Stoks, 2019). A mortality of 20% is usually set as an accepted threshold for regulatory bioconcentration experiments (OECD, 2012) which slightly exceeded for *G. pulex* at higher temperatures. Thus, the corresponding gammarid data should be interpreted with care. However, little deviation from the drawn regressions was observed if data of the 21°C treatment were excluded (SI A20). In pretests, specimens of *G. pulex* were observed to die spontaneously in the control medium at 23°C (35% mortality after 4 day), but much less at 21°C (15% mortality after 4 day). Thus, the highest temperature experiment was probably performed close to the physiological limit of the tested gammarid population.

### 3.3 Temperature effects on lipid and protein contents

Lipid and protein contents showed minor differences across the temperatures and thus are summarized in SI A6. The influence of size and lipid content on bioconcentration of the present compound mixture was investigated in a pretest (SI A9) using *G. pulex* and showed no lipid content dependency of the *BCF*_*24h*_ at 16°C (Figures S8 and S9). For completeness, lipid content normalized BCFs are provided in SI B4 and B5.

### 3.4 Temperature effects on toxicokinetic rates of the parent compounds

**FIGURE 2:**
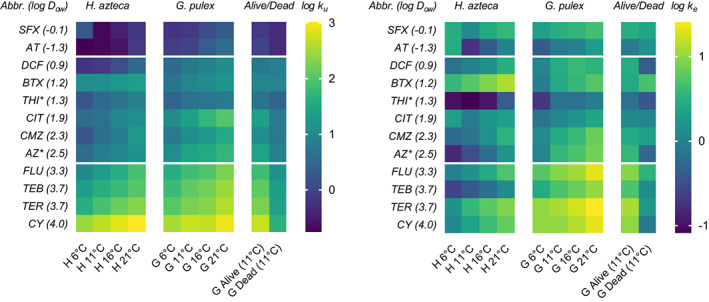
Heat map of the log‐normalized uptake (left) and elimination (right) rates modelled from the toxicokinetic experiments using the first‐order one‐compartment model. The log *D*_*ow*_ is shown in brackets behind the compound shortcut. Compounds are sorted by log *D*_*ow*_. AT, atenolol, AZ, azoxystrobin; BTX, benzotriazole; CIT, citalopram; CMZ, carbamazepine; CY, cyprodinil; DCF, diclofenac; FLU, fluopyram; SFX, sulfamethoxazole; TEB, tebuconazole; TER, terbutryn; THI, thiacloprid. * = AZ and THI showed two‐compartment kinetics (SI A10). The grouping is based on a cluster analysis of the uptake rate for *G. pulex* in Figure S22.

An overview of the modelled toxicokinetic rates for the parent compounds is presented in Figure *2*. Detailed values of the model fits and parameters (including calculated half‐life time *t*_*1/2*_ and *t*_*ss*_) are provided in SI A10 and A12.

The applied one‐compartment toxicokinetic models provided a good fit for most compounds and species. Few models were limited by concentrations falling below the LOQ (partially atenolol and sulfamethoxazole in *H. azteca*). In case of an apparent second compartment with slow elimination kinetics (azoxystrobin, thiacloprid), the one‐compartment model fit overestimated the elimination rates and resulted in large confidence intervals in both species (SI A10). Similar observations were made for another neonicotinoid (imidacloprid, Švara et al., 2021) and azoxystrobin (Kosfeld et al., 2020) elsewhere. As the interactions of the two compartments are not understood yet, no suitable two‐compartment model could be applied. Thus, the one‐compartment fits of the two compounds have to be interpreted carefully and are labelled accordingly.

All toxicokinetic rates showed an exponential increase with increasing temperature. *G. pulex* tended to have higher toxicokinetic rates than *H. azteca*. The toxicokinetic rates in alive gammarids at 11°C were much higher than in dead (heat shock inactivated) gammarids at the same temperature. Based on chemical properties, a trend of higher uptake and elimination rates with increasing log *D*_*ow*_ (− 0.1 to 4.0) was observed.

**FIGURE 3:**
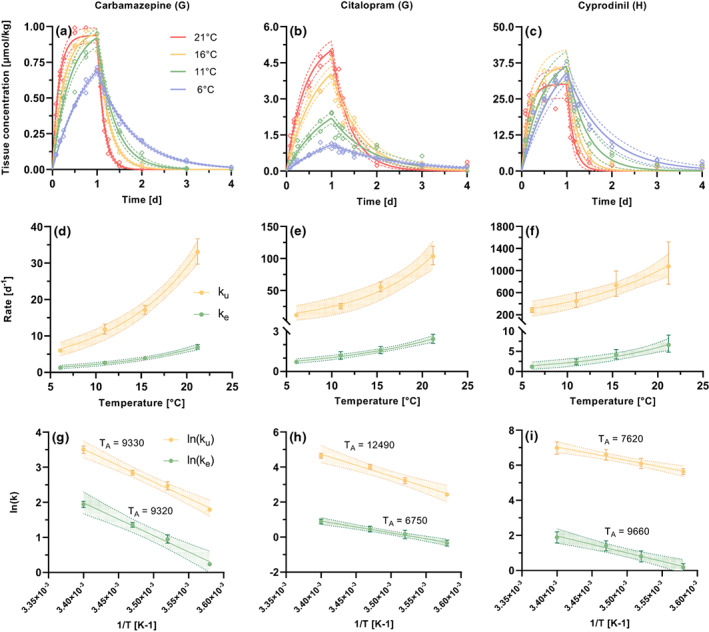
Exemplary comparison of the toxicokinetic model fits for three compounds with different patterns (a–c) in G = *G. pulex*, H = *H. azteca*. The exposure concentration was 50 μg L^−1^ for all compounds. Measured tissue concentrations are presented as data points, and the model fits as continuous lines with 95% CIs as dotted lines. Exponential increase fits (d–f) as well as the Arrhenius relationships (g–i) are shown in the rows below. The data points represent the modelled rates and 95% CIs. The lines represent the exponential increase (d–f) and linear regression (g–i) fits with the 95% CIs as dotted lines. Fit parameters are provided in SI A10 and SI A14. Please note different y‐axis scales.

**FIGURE 4:**
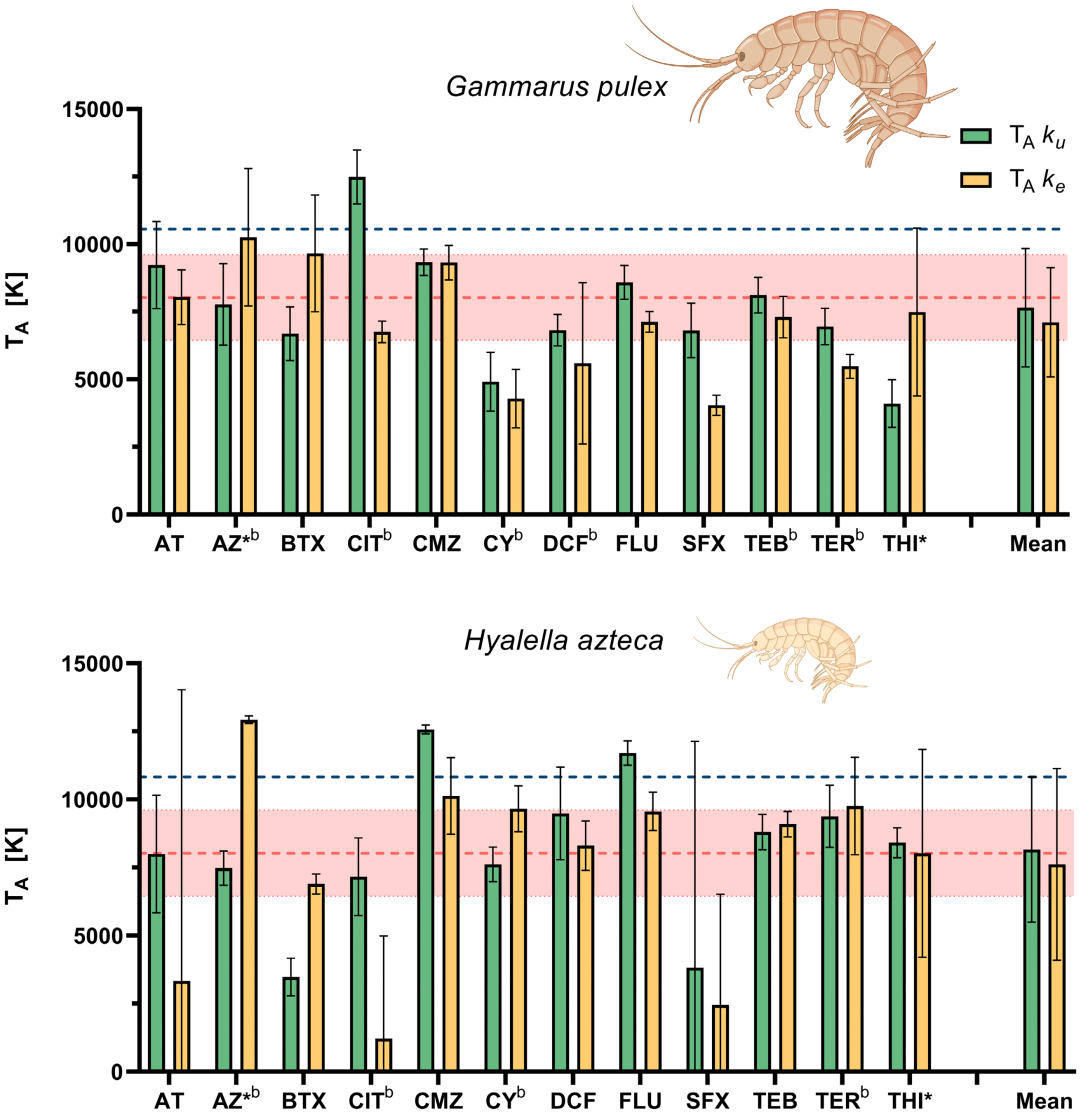
Arrhenius temperatures of the temperature‐dependent uptake and elimination rates in *Gammarus pulex* (top) and *Hyalella azteca* (bottom) (±SE). The dotted red lines represent the experimental determined physiological *T*_A_ (± SE) for *H. azteca*. The dark blue lines represent the *T*_*A*_ values from the AmP database. AT, atenolol; AZ, azoxystrobin; BTX, benzotriazole; CIT, citalopram; CMZ, carbamazepine; CY, cyprodinil; DCF, diclofenac; FLU, fluopyram; SFX, sulfamethoxazole; TEB, tebuconazole; TER, terbutryn; THI, thiacloprid. * = AZ and THI showed two‐compartment kinetics (SI A10). ^b^ = additional *T*_*A*_ values of the biotransformation models are presented in Figure 5. Underlying data are provided in SI A14.

Exemplary one compartment toxicokinetic model fits at the four different temperatures are presented in Figure 3 for three different compounds (a–c). Additionally, the exponential relationship between the rates and temperature is visualized (d–f) and the linear fits for the determination of *T*_*A*_ shown below (g–i). All Arrhenius fit parameters are provided in SI A14. The exponential relationship between temperature and the toxicokinetic rates could be described in three different patterns: (a) compounds where both uptake and elimination rates were affected by temperature proportionally (i.e. carbamazepine and most other compounds, see overlapping SE in Figure 4), (b) compounds where temperature exerted a higher impact on the uptake rates (citalopram and fluopyram in *G. pulex*) and (c) compounds where temperature exerted a higher impact on the elimination rates (cyprodinil in *H. azteca*). The modelled toxicokinetic rates were confirmed with results of a pretest with reduced sampling rates SI A11.

An overview of calculated values for *T*_A_ of the toxicokinetic rates in both species is presented in Figure 4. The overall average *T*_A_ was 7380 ± 2080 K and 7890 ± 3070 K (±SD) for *G. pulex* and *H. azteca* respectively. Both averages overlap with the experimentally determined physiological *T*_A_ (8030 ± 1580 K) and are close to the *T*_A_ from the AmP database (10,560 and 10,830, AmP, 2018). The *T*_A_ of most individual rates was also in range of the physiological *T*_A_. However, there were exceptions, such as lower *T*_A_s for both cyprodinil rates and *k*_e_ of sulfamethoxazole and terbutryn as well as a higher *T*_A_ of *k*_u_ of citalopram, in *G. pulex*. In *H. azteca*, the *T*_A_ of *k*_u_ of benzotriazole and *k*_e_ of citalopram were lower and the *T*_A_ of *k*_u_ of carbamazepine, fluopyram and the *k*_e_ of azoxystrobin were much higher than the values for the physiological *T*_A_.

### 3.5 Temperature effects on biotransformation rates

**FIGURE 5:**
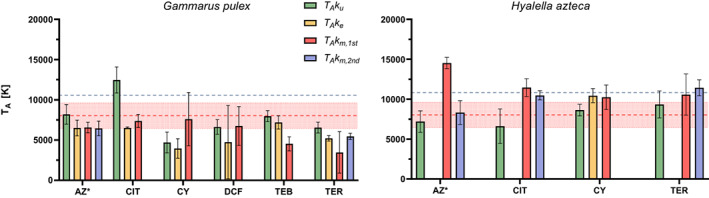
Arrhenius temperatures for the toxicokinetic rates calculated with the biotransformation model. *Gammarus pulex* (left) and *Hyalella azteca* (right) (±SE). The dotted red lines represent the experimental determined physiological *T*_A_ for *H. azteca*. The dark blue lines represent the *T*_A_ values from the AmP database. AZ, azoxystrobin; CIT, citalopram; CY, cyprodinil; DCF, diclofenac; TEB, tebuconazole; TER, terbutryn. * = AZ showed two‐compartment kinetics (SI A10). No *T*_A_ was calculated for *k*_e_ of AZ, CIT and TER in *H. azteca*, as *k*_e_ was close to zero due to the dominance of biotransformation in the total parent elimination. Underlying data are provided in SI A14.

For six compounds (azoxystrobin, citalopram, cyprodinil, diclofenac, tebuconazole, terbutryn) in *G. pulex* and four compounds in *H. azteca* (azoxystrobin, cyprodinil, citalopram, terbutryn), the BTP concentrations were sufficient (sum BTPs >5% of parent concentration after 24 h of exposure) to calculate first and second biotransformation rates at all temperatures. An overview of the corresponding model fits and determined parameters is provided in SI A12. The estimated biotransformation rates contributed only a minor proportion (<7%) to the total parent elimination of cyprodinil, tebuconazole and terbutryn in *G. pulex* and cyprodinil in *H. azteca*. For azoxystrobin (up to 60%), citalopram (up to 20%) and diclofenac (up to 40%) biotransformation contributed in higher proportions to the overall elimination in *G. pulex*. Comparatively, total elimination of azoxystrobin, tebuconazole and terbutryn was dominated by the contribution (mostly >90%) of biotransformation in *H. azteca* (Figure S32). Consequently, *k*_*e*_ was close to zero in these cases and thus omitted from the *T*_A_ comparison. The *T*_A_s of most biotransformation rates (Figure 5) were very similar between primary and secondary BTPs as well as *k*_*e*_ and stayed close to the physiological *T*_*A*_. This was not the case for *k*_*m1*_ of tebuconazole. Furthermore, the *T*_A_ of *k*_u_ and *k*_e_ stayed very similar across modelled parameters from both one‐compartment models with and without biotransformation (Figures 4 and 5), with the exception of citalopram in *H. azteca*. The latter had a much lower *T*_A_ of *k*_e_ in the parent model than the *T*_A_ of the biotransformation rates, which was in range of the physiological *T*_*A*_.

The biotransformation models were limited by the high number of parameters and the fits resulted in high uncertainties of the BTP elimination rates. In case of diclofenac, one of the main BTPs (diclofenac taurine) could not be quantified due to its low ionization efficiency in the applied method (Fu et al., 2021). Generally, models are likely to be limited by the quantification of most BTPs using the parent calibration due to missing reference material, which results in higher uncertainties.

### 3.6 Temperature and species dependence of the bioconcentration factor

**FIGURE 6:**
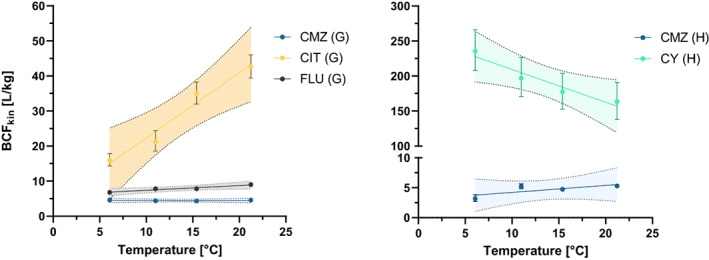
Temperature and BCF_kin_ (±95% CI) relationship in *Gammarus pulex* (left) and *Hyalella azteca* (right). CIT, citalopram; CMZ, carbamazepine; CY, cyprodinil; FLU, fluopyram. CMZ is presented as a reference of a temperature stable BCF_kin_, whereas the BCF_kin_ of CIT and FLU showed a positive and CY a negative relationship with temperature in *G. pulex* and *H. azteca* respectively.

For most compounds, the BCF_kin_ remained stable across different temperatures (slope not significantly different from zero; SI A15) because *k*_u_ and *k*_*e*_ were similarly affected by temperature (pattern of carbamazepine, Figures 3 and 4). However, for some compounds, temperature showed an effect on the BCF_kin_ (slope significantly different from zero, p < 0.05). The affected compounds were citalopram and fluopyram in *G. pulex*, as well as cyprodinil in *H. azteca* (Figure 6). The BCF_kin_ in *G. pulex* was increasing 2.7 (citalopram) and 1.3 fold (fluopyram) across the temperature range, whereas the decrease was down to 0.7 for cyprodinil in *H. azteca*. The *BCF*_*24h*_ (SI A10) of the mentioned compounds showed a similar trend as the BCF_kin_.

The BCF_kin_ of azoxystrobin decreased down to 0.4 along the temperature ranges in *H. azteca*. However, the *BCF*_*24h*_ of azoxystrobin remained stable between 11 and 21°C (6.3 ± 0.3, *H. azteca* and 5.8 ± 0.5, *G. pulex*) and most likely represented steady‐state conditions. Thus, it is concluded that the observed temperature dependence of the BCF_kin_ for azoxystrobin might have been an artefact of an oversimplified model. The creation of a suitable two‐compartment model would allow a re‐evaluation.

The BCF_kin_ was generally very similar between the two species (SI A16). However, gammarids tended to have a higher BCF_kin_ than *H. azteca* for low accumulative (log BCF_kin_ <0.65) compounds and *H. azteca* a higher BCF_kin_ for more accumulative compounds. Citalopram represented a clear outlier from this relationship as it showed much higher and temperature‐dependent BCF_kin_ in *G. pulex* compared to *H. azteca*. Lipid normalization was avoided according to SI A9 (see also above).

### 3.7 Influence of physiological activity

The comparison of toxicokinetics in living and dead gammarids (Figure 2) showed much faster kinetics, up to a factor of 12 (cyprodinil) for both *k*_u_ and *k*_*e*_, in living gammarids compared to in dead ones. The differences are probably due to the absence of filtration activity in the dead gammarids, which results in slower diffusion processes. However, their BCF_kin_s (SI A17) were much less different. For most compounds, no BTPs were detected in the extracts of the dead gammarids, leading to the assumption that biotransformation enzymes have been inactivated by denaturation. Only for azoxystrobin, some hydrolysis TPs were detected in the dead gammarids, possibly formed abiotically, but no phase 2 BTPs. Consequently, a higher BCF_kin_ of azoxystrobin (factor 2) and diclofenac (factor 8) in dead gammarids could potentially be explained by the absence of biotransformation in the inactivated gammarids. Both compounds had a higher contribution of biotransformation on the total elimination than other compounds (Figure S32). The BCF_kin_ of the cations atenolol and citalopram was higher (factor 4) in alive gammarids.

### 3.8 Modelled environmental scenarios

**FIGURE 7:**
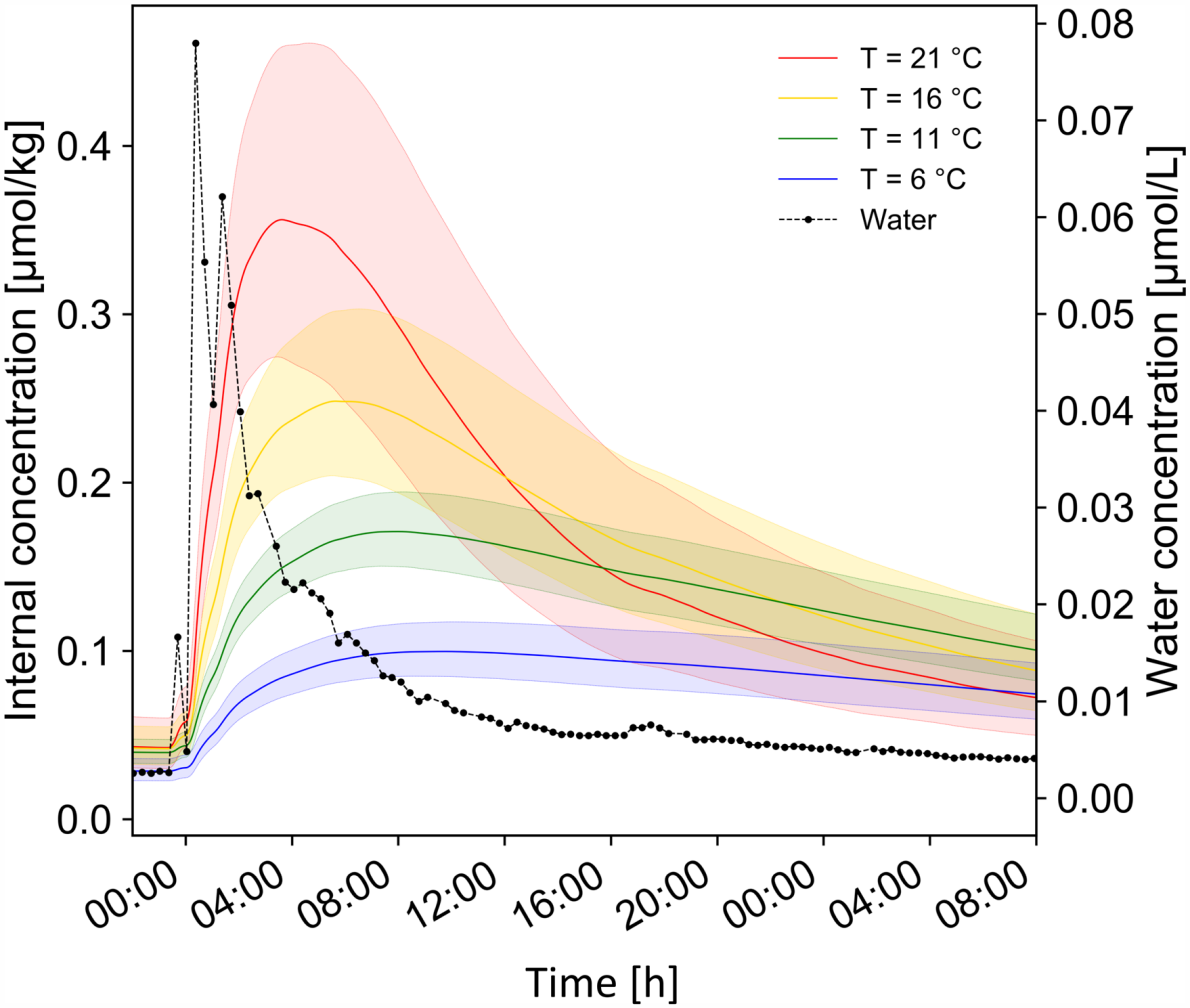
Temperature‐dependent internal concentration of fluopyram in *Hyalella azteca* during a monitored run‐off event (Lauper et al., 2021). Internal concentrations ±95% CI were modelled using the toxicokinetic rates determined in the present study.

The modelled toxicokinetic rates were applied to two different exposure scenarios: short‐term exposure peak and daily temperature fluctuation. The modelled internal concentration of fluopyram in *H. azteca* during an empirically determined short‐term exposure peak at the four different temperatures (Figure 7) showed an increasing internal peak concentration with increasing temperature resulting in a three to four times difference between the lowest and the highest temperature. Towards the decline of the water concentration, the internal concentration at the higher temperatures fell below the internal concentration at lower temperatures. A similar pattern was observed for *G. pulex* but with a faster elimination, due to the higher elimination rates (Figure S45). Furthermore, internal concentration profiles of cyprodinil in both species are presented in SI A19, which show similar trends than fluopyram.

**FIGURE 8:**
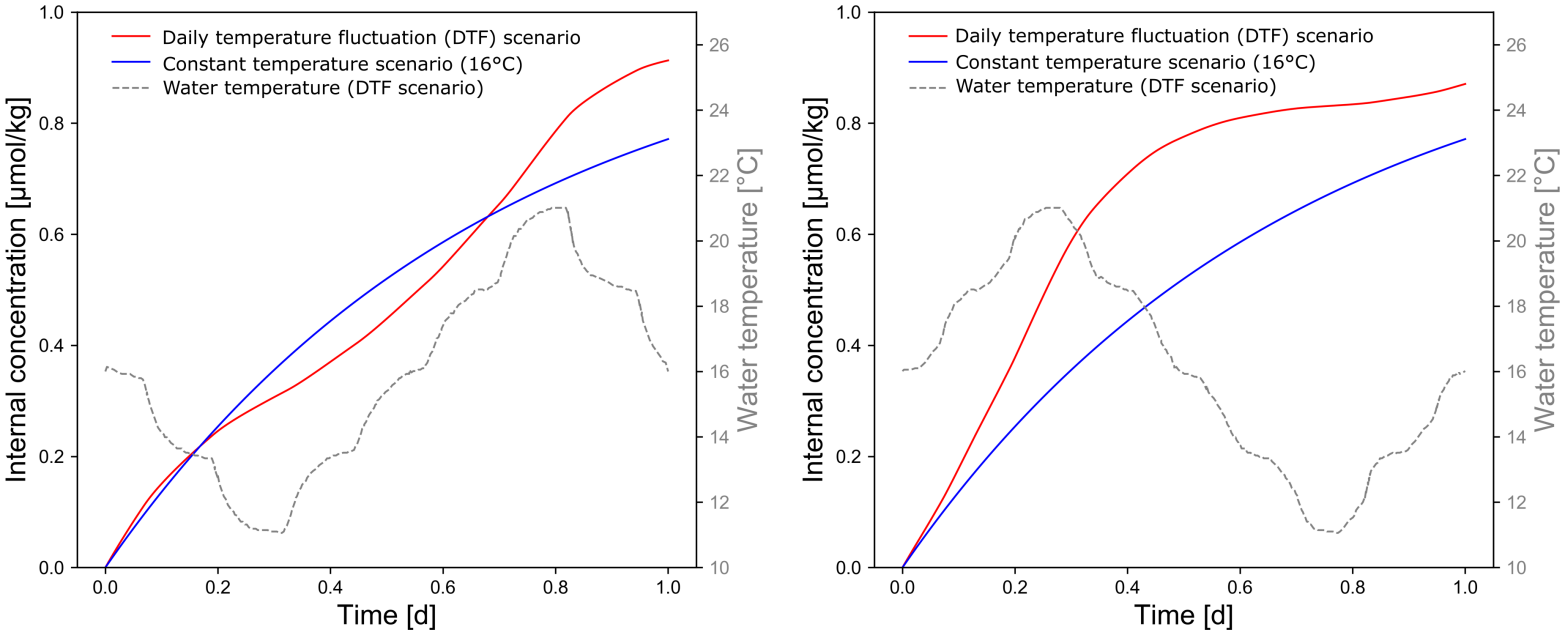
Modelled carbamazepine concentrations in *Hyalella azteca* during a daily temperature fluctuation (DTF) and constant temperature (16°C) scenario. Temperature profile starting with decreasing temperature (left) and increasing temperature (right).

The modelling of internal concentrations in a daily temperature fluctuation (DTF) scenario at a stable exposure concentration (Figure 8) showed that the internal concentration during the non‐equilibrium phase of 1 day was higher in the DTF scenario compared to a stable temperature. The difference was larger if the profile started with increasing temperature.

## 4 DISCUSSION

In the present study, increasing temperature caused an exponential increase of toxicokinetic rates in both amphipod species, which is contrary to the common assumption of toxicokinetic rates being constant parameters. However, the effect of temperature on equilibrium conditions (BCF_kin_) was negligible for most of the compounds. In the following section, the underlying mechanisms and consequences for environmental risk assessment are discussed.

### 4.1 Temperature dependence of toxicokinetic processes

The modelled kinetic BCFs remained mostly unaffected by different temperature treatments or showed very little temperature dependence (fluopyram), except for citalopram and cyprodinil. Thus, the primary temperature effect was an expansion or compression on the experimental time axis (Nørhave et al., 2014) with uptake and elimination rates being affected by temperature in the same magnitude. Thus, by calculating the BCF_kin_ from the ratio of both rates, the temperature effects on the rates equalled out. The determined toxicokinetic rates and BCFs were all in range of available parameters from previous experiments if compared to the correct corresponding temperature (SI A13). All compounds had a BCF_kin_ way below the regulatory threshold of 2000 (B criterion, European Commission, 2006) and are thus not considered bioaccumulative in a regulatory perspective.

The application of the Arrhenius equation provided a good approximation of the temperature dependency of the toxicokinetic rates in comparison to physiological rates such as the determined standard metabolic rates. Even though the Arrhenius equation is designed to be applied under the assumption of a single, rate‐limiting, thermally activated process (Laidler, 1984), the present integration of different chemical (i.e. diffusion) as well as biological (i.e. filtration, biotransformation) processes seemed to be applicable. Most toxicokinetic *T*_A_s were overlapping with the physiological *T*_A_s. Thus, a single species‐specific *T*_A_ determined in respiration experiments or obtained from a database (AmP, 2018) could be implemented as approximation into toxicokinetic models in order to account for temperature effects (i.e. Equation 8). Furthermore, the physiological *T*_A_ seems to be very similar across a broad range of invertebrate taxa (AmP, 2018; Camp & Buchwalter, 2016; Dai et al., 2021; Gergs et al., 2019), indicating a universally similar temperature response of ectotherms. However, it should be noted that the SE of toxicokinetic‐related *T*_A_s accounted for up to 28% (*G. pulex*) and 38% (*H. azteca*) of the modelled value, which may be caused by the accumulation of uncertainties from biological variance, chemical analysis and toxicokinetic modelling as well as compound‐specific differences.

The temperature effects on toxicokinetics may be driven by passive diffusion, enhanced by physiological activity (i.e. filtration) as suggested earlier by Dai et al. (2021). It has been demonstrated that filtration surface and activity is an important driver of toxicokinetics (Buchwalter et al., 2003). This could also be supported by the observed much higher uptake rates in alive gammarids than dead gammarids, but very similar kinetic BCFs. Additionally, Nawaz and Kirk (1996) reported a high impact of physiological activity on toxicokinetics by comparing internal concentrations of dead and living *Daphnia magna*. Furthermore, the *T*_A_ of biotransformation rates showed a very good agreement with the physiological *T*_*A*_. Physiological rates of ectotherms are highly affected by temperature changes within the physiological acceptable range (Hill et al., 2018) whereas diffusion following Fick's law may be less influenced by temperature in the range of physiological temperatures (Laidler, 1984), as changes in physiological temperature are relatively small compared to the absolute temperature. Toxicokinetics may also be influenced by membrane permeability directly affected by temperature or indirectly affected by changes in lipid composition as well as membrane‐bound enzyme density and activity reported in crustaceans (Lahdes et al., 2010; Pruitt, 1990). The convergence of test temperatures with an exceedance of physiological optimum temperatures may result in decreasing respiration rates (Galic & Forbes, 2017) and enzymatic activity (Jakob et al., 2021) in amphipods. In such cases, non‐classic Arrhenius relationships may apply (Arroyo et al., 2022; Meynet et al., 2020). However, such limitations (i.e. reduced rates at higher temperatures) were not observed in the present data set.

An analysis of a previously conducted study (Dai et al., 2021) with terrestrial worms (*Enchytraeus albidus*) exposed to phenanthrene (log K_ow_ 4.5) estimated a very similar *T*_A_ (7530 ± 860 K) for the uptake rate compared to physiological *T*_A_s of different earthworm species (SI A18), but also *H. azteca* in the present study. However, the *T*_A_ of elimination in earthworms was smaller (4940 ± 700 K), which resulted in an increasing BCF_kin_ with increasing temperature. The lower temperature dependency of the elimination may be caused by mostly passive elimination due to lacking filtration activity in terrestrial organisms or due to partitioning into lipids as an additional compartment.

The applicability of the Arrhenius relationship to other studies for ectotherms could not be tested, because either only two temperatures were studied, no toxicokinetic rates were modelled or no equilibrium conditions were confirmed (Brown et al., 2021; Buchwalter et al., 2003; Camp & Buchwalter, 2016; Cerveny et al., 2021; Geisler et al., 2012; Kuo & Chen, 2021; Muijs & Jonker, 2009; Nawaz & Kirk, 1996). The different experimental scopes and approaches may also be the reason for different reported relationships between temperature and BCF_kin_. Especially because the equilibrium conditions are much later reached at lower temperatures (Figure 3), this could mistakenly be reported as a positive bioconcentration–temperature relationship if experimental times are not chosen sufficiently or no kinetic BCFs are derived. This underlines the necessity of kinetic modelling approaches or confirmation of equilibrium conditions in bioaccumulation research.

### 4.2 Species differences

The two species showed minor differences in the calculated and modelled BCF_kin_. However, *k*_*u*_ and *k*_*e*_ were generally higher in *G. pulex*. Contrastingly, due to the higher surface to volume ratio, it would have been predicted that toxicokinetic rates are higher in *H. azteca*. It appears that other parameters, such as filtration rates, have a higher impact on the uptake and elimination kinetics (Buchwalter et al., 2003). This was shown by comparing the *T*_A_ of the standard metabolic rates and toxicokinetic rates. However, no related data on such parameters (i.e. O_2_ consumption) were available for the comparison of the two species. In both species, the response of toxicokinetic rates to temperature was similar, represented by similar *T*_A_s.

Even though lipid content normalization could potentially explain the higher BCFs of less polar compounds in *H. azteca* (two to three times higher lipid content), lipid normalization has to be handled carefully. The present and supplementing experiments with *G. pulex* of different lipid contents resulted in similar tissue concentrations of the exposed chemicals (SI A9). The experiments with *G. pulex* also covered the range of lipid contents of *H. azteca* in the present study. Furthermore, Arts et al. (1995) observed that an accumulated moderately lipophilic contaminant (triallate, log *K*_OW_ = 4.4) was indeed associated with lipid‐rich compartments in *H. azteca* and *Gammarus lacustris* (Sars, 1863). However, the accumulated amount did not always correlate to the total lipid content. Thus, it is likely that lipid content plays a minor role in accumulation of polar compounds or lipid composition is more important than total lipid content (Ewald & Larsson, 1994).

Another species‐specific observation in our study was a strong temperature dependency of the BCF_kin_ of citalopram in *G. pulex*. This was caused by a much higher acceleration of the uptake rate by temperature compared to the elimination rate. However, the same trend was not observed in *H. azteca*. A carrier‐mediated mechanism of citalopram uptake in mammalian brain cells was reported by Rochat et al. (1999). This transport process was saturable and temperature dependent. Furthermore, active uptake mechanisms of antidepressants into the nervous tissue of fish were suggested (Grabicova et al., 2014; Schultz et al., 2010). These pathways may also exist in amphipods and mechanistically explain the divergent temperature‐related behaviour of citalopram in *G. pulex* compared to other compounds. As the specification of the tertiary amine citalopram is similar (>95% cationic) in the two test systems, the difference may be rather explained by the higher biotransformation in *H. azteca*, which corresponds with the absence of the secondary amine dealkylation product didesmethylcitalopram (a main BTP of citalopram of humans (Sangkuhl et al., 2011)) in *G. pulex*. Differences in biotransformation pathways between *H. azteca* and *G. pulex* have been observed before (Fu et al., 2018, 2020). The decreasing BCF_kin_ of cyprodinil in *H. azteca* with increasing temperature may also be explained by biotransformation. However, this is speculative until further elucidation of the biotransformation pathways of cyprodinil.

It could be possible that biotransformation of the exposed compounds was affected by the exposure mixture. For instance, azole fungicides inhibit CYP‐450 enzymes in *G. pulex*, but only in higher concentrations than in the present study (Fu et al., 2018; Rösch et al., 2017). Furthermore, the presence of CYP‐biotransformed compounds could result in a CYP induction as reported for crustaceans and other aquatic invertebrates (Ashley et al., 1996; Cedergreen et al., 2021; Snyder, 2000). Additionally, antibiotics such as sulfamethoxazole could have affected the gut microbiome (Edlund et al., 2012; Gorokhova et al., 2015), which potentially contributes to biotransformation through bacterial enzymes. However, the toxicokinetic parameters obtained (parent and biotransformation model) in the present study were very consistent (overlapping CIs) with previous single compound toxicokinetic experiments using *H. azteca* (Fu et al., 2018, azoxystrobin; Kosfeld et al., 2020, terbutryn). For some compounds, toxicokinetic parameters of *G. pulex* were consistent with data obtained from a different exposure mixture tested in gammarids of the same population (Arlos et al., 2020, benzotriazole, diclofenac and sulfamethoxazole), but differed for other compounds and/or other gammarid populations (atenolol, carbamazepine and citalopram, see SI A13). Thus, we conclude that the transfer of our results towards individual compounds or other exposure mixtures and concentrations may be generally applicable, especially for controlled laboratory cultures (at present, laboratory cultures of gammarids are difficult to establish, Alther et al., 2023). However, data have to be interpreted more carefully when applied to organisms originating from a less controlled environment and different clades or populations.

### 4.3 Model implementations

The modelled environmental scenarios demonstrated that the temperature dependence of toxicokinetics could have a very high impact on the internal concentration during short‐term exposure peaks. During such events, usually no steady‐state conditions are reached; thus, the uptake rate is the main determinant of internal concentrations. The exponential relationship of temperature and uptake rates may strongly increase the risk of such short‐time exposure events compared to estimations from laboratory studies as shown for a real‐world exposure scenario (Figure 7). The interaction of increasing temperature and organic contaminants, promoting toxic effects in amphipods, may be especially alarming in small (agricultural) streams in spring and summer. The combination of higher temperatures (Baumgartner & Robinson, 2015; Dalhoff et al., 2018) in these months and the peak of pesticide concentrations (Arlos et al., 2020; Chow et al., 2020; Lauper et al., 2021; Munz et al., 2017; Phillips & Bode, 2004) may be a key driver of acute effects in amphipods.

In the DTF scenario, a slightly higher internal concentration of carbamazepine was calculated compared to the constant average temperature. This was caused by the exponential temperature relationship, which leads to a greater increase of the uptake rate during high‐temperature periods than the reduction during the low‐temperature periods (Jensen's inequality rule; Denny, 2017). The internal concentration increase was in a similar range observed in a DTF experiment with earthworms (Dai et al., 2021). However, their measured internal concentrations under DTF conditions were slightly higher than predicted from non‐linear extrapolations based on the constant exposure temperatures. It was suggested that this could be caused by an increase of metabolic activity in stress situations. The same effect would potentially be observed in experiments with aquatic invertebrates. Nevertheless, the toxicokinetics in the DTF scenario would not explain the strong increase in sensitivity of aquatic invertebrates towards contaminants demonstrated elsewhere (Verheyen et al., 2022; Verheyen & Stoks, 2019). Thus, the observed increase in toxicity was most likely caused by toxicodynamic (i.e. combined stress) but not toxicokinetic mechanisms.

The present research enabled the explicit modelling of temperature effects on toxicokinetics alone. However, additional toxicodynamic models would be required in order to evaluate resulting toxic effects. Alternatively, toxicokinetic–toxicodynamic models including temperature corrections (i.e. Arrhenius based) have been successfully used recently to analyse temperature‐dependent toxicity directly, but thereby reducing mechanistic toxicokinetic information (Goussen et al., 2020; Mangold‐Döring et al., 2022; Rakel et al., 2022).

### 4.4 Practical considerations for the risk assessment

The relative similarity of the two organisms regarding toxicokinetics of the tested compounds allows the assumption that *H. azteca* may be preferred in bioaccumulation assessment frameworks for practical reasons. The simple cultivation provides a homogenous test population throughout the year and many standardized test protocols are available. Furthermore, different genetic lines have been sequenced and comprehensive toxicogenomic studies have been performed (Poynton et al., 2018). As the BCF_kin_ of more lipophilic compounds was higher in *H. azteca*, it would also be the more conservative test system in bioaccumulation assessments.

However, gammarids are much more sensitive towards changes in environmental parameters, such as temperature (Cottin et al., 2012; Fulton et al., 2021). Furthermore, gammarids also tend to show similar or higher sensitivity in acute and chronic toxicity tests than *H. azteca* (Beaudouin & Péry, 2013; Brock et al., 2018; Bustos et al., 2022; Roman et al., 2007). This was shown despite higher temperatures—and thus even longer physiological time (Nørhave et al., 2014)—in experiments with *H. azteca*. Thus, *G. pulex* may be the preferred organism to study climate change impacts on the toxicity of contaminants.

## 5 CONCLUSION

Our results emphasize the importance of considering temperature effects when assessing toxicokinetics in ectothermic organisms in order to account for global climate change scenarios. We demonstrated that the application of the Arrhenius theory provides a good estimate to account for temperature effects on toxicokinetics of organic contaminants in two different aquatic amphipods, *G. pulex* and *H. azteca*, which are representing important key species in the aquatic food webs worldwide. The effect of temperature on toxicokinetics may result in higher toxicity, especially in short‐term exposure scenarios such as run‐off after pesticide application, whereas temperature effects may potentially be less important for bioaccumulation (B criterion) assessment. However, further investigations are needed to understand the mechanisms behind compounds with a temperature effect on the BCF_kin_ (i.e. citalopram). Finally, the combined risk of chemical pollution and climate change is a complex challenge, as changes in climate do not only influence toxicokinetics and toxicodynamics directly, but also pollutant dynamics such as different pesticide application and leaching patterns, and thus exposure profiles.

## CONFLICT OF INTEREST

The authors declare no competing financial interest.

## Supporting information


Data S1

## Data Availability

The data that support the findings of this study are openly available in the Eawag Research Data Institutional Collection (ERIC) at https://doi.org/10.25678/000779.
