# Supplementary material for: Speed it up: How temperature drives toxicokinetics of organic contaminants in freshwater amphipods
Source: Glob Chang Biol. 2022 Dec 9;29(5):1390–406. doi: 10.1111/gcb.16542 (PMC10107603; doi:10.1111/gcb.16542)
Supplement: Supplementary file 1 — Data S1 [file GCB-29-1390-s001.zip › GCB_16542_raths_temperature_SI_A.pdf]

Supporting Information A:

Speed it up: How temperature drives toxicokinetics of organic contaminants in freshwater amphipods

J. Raths[1,2], V. Švara[3,4], B. Lauper[1,2], Q. Fu[1], J. Hollender[1,2]*

[1]Department of Environmental Chemistry, Swiss Federal Institute of Aquatic Science and Technology - Eawag, Dübendorf, Switzerland

[2]Institute of Biogeochemistry and Pollutant Dynamics, ETH Zürich, Zürich, Switzerland

[3]UNESCO Chair on Sustainable Management of Conservation Areas, Engineering & IT, Carinthia University of Applied Sciences, Villach, Austria

[4]Department of Effect-Directed Analysis, Helmholtz Centre for Environmental Research - UFZ, Leipzig, Germany

E-mail: juliane.hollender@env.ethz.ch

Contents

Table of Figures III

Table of Tables........ V

SI A1: Animal husbandry 1

SI A2: Genetic specification 1

Polymerase chain reactions, sequencing, and genotyping information 1

COI PCR and sequencing reactions 1

Genotyping using microsatellite markers........ 1

Genetic variation analysis........ 2

COI sequence data........ 2

Microsatellite data 2

Principal component analysis........ 2

Summary of the genetic specification results 3

SI A3: Test compounds 4

SI A4: Wet weight – dry weight conversion factors 5

SI A5: Chemical analysis 5

Online SPE LC-HRMS/MS settings 5

Analytical quality parameters 7

Biotransformation product evaluation........ 10

SI A6: Determination of lipid and protein contents 14

Lipid and protein methods 14

Lipid and protein results........ 14

SI A7: Calculation of time to 95% steady state t_{ss} and elimination half-life $t_{1/2}$ 16

SI A8: Standard metabolic rate measurements 16

Standard metabolic rate methods 16

Standard metabolic rates results 17

SI A9: Pre-test on temperature dependent toxicokinetics and investigations on the influence of organism size and lipid content........ 19

Methods: Gammarus pulex........ 19

Methods: Exposure scenarios 19

Methods: Sample analysis........ 19

Methods: Bioconcentration 19

Results: Lipid content........ 20

Results: Bioconcentration – size and lipid effect 20

Results: Bioconcentration – temperature effect........ 21

SI A10: Modelled toxicokinetic parameters (parent models) 23

SI A11: Confirmation of the modelled toxicokinetic parameters using the pre-test dataset 35
SI A12: Modelled toxicokinetic parameters (biotransformation models) 39
BTP classification 39
Modelled parameters 42
SI A13: Comparison of modelled toxicokinetic parameters with literature values 48
SI A14: Arrhenius temperature calculations 51
SI A15: Temperature dependence of the kinetic BCF 55
SI A16: Species differences in the kinetic bioconcentration factor 56
SI A17: Dead vs. alive gammarids 58
SI A18: Arrhenius temperature calculations from Dai et al. (2021) 60
SI A19: Modelled simulations of different exposure and climate scenarios 61
SI A20: Effect of the exclusion of the 21°C *G. pulex* data set on calculated Arrhenius temperatures .. 61
References 64

Table of Figures

Fig. S1: PCA plot of genotyped individuals 2

Fig. S2: Chromatogram of cyprodinil BTP spiked and unspiked gammarid sample. 9

Fig. S3: Overview of the possible hydroxylation BTPs of cyprodinil (= CYM242). 9

Fig. S4: Lipid (top) and protein (bottom) contents in the two amphipod species. 15

Fig. S5: SMR (dry weight) of H. azteca across the four tested temperatures. 17

Fig. S6: SMR (ww) for the three compared studies. 17

Fig. S7: Arrhenius relationships of the SMR. 20

Fig. S8: Lipid content of the two size classes presented as mean ± SD (n = 3). 18

Fig. S9: BCF_{24h} of the two gammarid size classes at 16°C. 20

Fig. S10: Calculated BCF_{6h} across the tested temperature range. 21

Fig. S11: Calculated BCF_{24h} across the tested temperature range. 22

Fig. S12: Toxicokinetic models (parent model) of atenolol, azoxystrobin and benzotriazole in *G. pulex*. 24

Fig. S13: Toxicokinetic models (parent model) of carbamazepine, citalopram and cyprodinil in *G. pulex*. 25

Fig. S14: Toxicokinetic models (parent model) of diclofenac, fluopyram and sulfamethoxazole in *G. pulex*. 26

Fig. S15: Toxicokinetic models (parent model) of tebuconazole, terbutryn and thiacloprid in *G. pulex*. 27

Fig. S16: Toxicokinetic models (parent model) of atenolol, azoxystrobin, benzotriazole, carbamazepine, citalopram and cyprodinil in alive vs. dead *G. pulex*. 28

Fig. S17: Toxicokinetic models (parent model) of diclofenac, fluopyram, sulfamethoxazole, tebuconazole, terbutryn and thiacloprid in alive vs. dead *G. pulex*. 29

Fig. S18: Toxicokinetic models (parent model) of atenolol, azoxystrobin and benzotriazole in *H. azteca*. 30

Fig. S19: Toxicokinetic models (parent model) of carbamazepine, citalopram and cyprodinil in *H. azteca*. 31

Fig. S20: Toxicokinetic models (parent model) of diclofenac, fluopyram and sulfamethoxazole in *H. azteca*. 32

Fig. S21: Toxicokinetic models (parent model) of tebuconazole, terbutryn and thiacloprid in *H. azteca*. 33

Fig. S22: Heat maps and clustering of the log-normalised uptake and elimination rates 34

Fig. S23: Modelled and individual measured concentrations of the pre-test, part 1/3....................... 36

Fig. S24: Modelled and individual measured concentrations of the pre-test, part 2/3....................... 37

Fig. S25: Modelled and individual measured concentrations of the pre-test, part 3/3........................ 38
Fig. S26: Biotransformation pathways of citalopram in *G. pulex* and *H. azteca* 41
Fig. S27: Heat map of the modelled toxicokinetic rates (biotransformation model). 42
Fig. S28: Toxicokinetic models (biotransformation model) of azoxystrobin, citalopram and cyprodinil in *G. pulex*.. 43
Fig. S29: Toxicokinetic models (biotransformation model) of diclofenac, tebuconazole and terbutryn in *G. pulex*.. 44
Fig. S30: Toxicokinetic models (biotransformation model) of azoxystrobin, citalopram and cyprodinil in *H. azteca*.. 45
Fig. S31: Toxicokinetic models (biotransformation model) of diclofenac, tebuconazole and terbutryn in *H. azteca*.. 46
Fig. S32: A comparison of the modelled elimination and primary biotransformation rates. 47
Fig. S33: Arrhenius fits for the calculated toxicokinetic rates of the parent model in *H. azteca*.......... 52
Fig. S34: Arrhenius fits for the calculated toxicokinetic rates of the parent model in *G. pulex*............ 52
Fig. S35: Arrhenius fits for the calculated toxicokinetic rates of the biotransformation model in *H. azteca*.. 54
Fig. S36: Arrhenius fits for the calculated toxicokinetic rates of the biotransformation model in *G. pulex*. 54
Fig. S37: Relationship between temperature and BCF_{kin} of the biotransformation model. 55
Fig. S38: Relationship between temperature and BCF_{kin} of the parent model. 55
Fig. S39: Linear regression of the log BCF_{kin} in *G. pulex* and *H. azteca*.. 56
Fig. S40: Comparison of the BCF_{kin} of *G. pulex* and *H. azteca* 57
Fig. S41: Uptake rate compared between dead and alive *G. pulex*. 58
Fig. S42: Elimination rate compared between dead and alive *G. pulex*... 58
Fig. S43: Kinetic bioconcentration factor compared between dead and alive *G. pulex*. 59
Fig. S44: Arrhenius relationships drawn from Dai et al. (2021). 60
Fig. S45: Temperature dependent internal concentration of fluopyram in *G. pulex* during a monitored run off event (Lauper et al. 2021). 61
Fig. S46: Temperature dependent internal concentration of cyprodinil in *G. pulex* during a monitored run off event (Lauper et al. 2021). 62
Fig. S47: Temperature dependent internal concentration of cyprodinil in *H. azteca* during a monitored run off event (Lauper et al. 2021). 62
Fig. S48: Comparison of Arrhenius temperatures using the full data set and a reduced data set, which excluded the 21°C treatment 623

Table of Tables

Tab. S1: Physico-chemical properties and modes of action of the selected test-compounds. 4

Tab. S2: Experimentally determined wet and dry weight conversion factors 5

Tab. S3: Schedule of the online-SPE. 5

Tab. S4: Schedule of the liquid chromatography. 6

Tab. S5: Source parameters used for HRMS/MS measurement 6

Tab. S6: MS parameters used for HRMS/MS measurement with the Q Exactive mass spectrometer... 6

Tab. S7: Analytical quality parameters of the quantified parent compounds. 7

Tab. S8: Analytical quality parameters of the quantified biotransformation products (BTPs). 8

Tab. S9: Inclusion list and detection of BTPs. 10

Tab. S10: Exposure scenarios of the pre-test. 19

Tab. S11: Biotransformation product classification for the biotransformation models. 40

Tab. S12: Comparison of the modelled toxicokinetic parameters (parent model, *G. pulex*) of the present study with literature values. 48

Tab. S13: Comparison of the modelled toxicokinetic parameters (biotransformation model, *G. pulex*) of the present study with literature values. 49

Tab. S14: Comparison of the modelled toxicokinetic parameters (parent model, *H. azteca*) of the present study with literature values. 50

Tab. S15: Comparison of the modelled toxicokinetic parameters (biotransformation model, *H. azteca*) of the present study with literature values. 50

Tab. S16: Arrhenius temperatures of the toxicokinetic rates of the parent model. 51

Tab. S17: Arrhenius temperatures of the toxicokinetic rates of the biotransformation model. 53

SI A1: Animal husbandry

Sampled gammarids were brought to the laboratory and gradually acclimated to aerated artificial pond water (APW) (Naylor et al., 1989) at 16°C for three days before further acclimation to the experimental temperatures. *Hyalella azteca* (Saussure, 1858) were kept in supplemented cupper reduced tap water containing bromide, which is essential for *H. azteca* (Borgmann, 1996). Culture and maintenance procedures for *H. azteca* were as described by Schlechtriem et al. (2019). No sexing was performed for both species, but females carrying egg as well as gammarids parasitized by acanthocephalans (Tain et al., 2006) were excluded from experiments. Only adult specimens were used (gammarid body length > 0.8 cm (average wet weight of 23.4 ± 4.2 mg), hyalella age > 3 months (average wet weight of 3.1 ± 0.4 mg).

SI A2: Genetic specification

For species genetic characterization, genomic DNA was extracted from pereopods of 60 *Gammarus pulex* (Linnaeus, 1758) and 4 *Gammarus. fossarum* (Koch, 1836) individuals from sampling sites Grüningen (Mönchaltdorfer Aa, 47.2749 °N, 8.7892 °E) and Müsterlingen (Seebach, 47.3735 °N, 9.1436 °E), Switzerland, using the Qiagen DNeasy Blood & Tissue kit. DNA integrity and concentrations were checked on an agarose gel and with a NanoDrop spectrophotometer (Technologies Inc.). For twenty samples per site, a fragment of mitochondrial cytochrome oxidase I (COI) was amplified with polymerase chain reaction (PCR) and sequenced using LCO and HCO primers (Folmer et al., 1994).

Polymerase chain reactions, sequencing, and genotyping information

COI PCR and sequencing reactions

Each PCR reaction consisted of 2.5 µL of 10 mM dNTPs, 10 µL 5X Green GoTaq Flexi Buffer (Promega), 4 µL of 25 mM $MgCl_2$, 1 µL of GoTaq DNA polymerase, 5 µL of 10 µM primers (Folmer et al., 1994), and 22.5 µL of deionised water. To each reaction, 5 µL of DNA template with concentration between 40-80 ng/µL was added. PCR consisted of an initial activation step of 2 min at 95 °C, followed by 34 cycles of 1 min denaturation at 95 °C, 45 s annealing at 51 °C and 1 min elongation at 72 °C. The reaction was terminated after the final elongation of 5 min at 72 °C. The PCR products were checked on the agarose gel and cleaned using GeneJET PCR Purification Kit (Thermo Fisher Scientific) following the kit instructions. The 10 µL sequencing reaction of 150-250 ng DNA with 1 µL of the sequencing primer, Big dye mix (Thermo Fisher Scientific), and 5x sequencing buffer, respectively, was prepared for each PCR product. The program of 1 min at 96 °C, 30 cycles of 10 s at 96°C, 5 s at 50 °C and 4 min at 60 °C was applied. The products were purified by ethanol/EDTA (ethylenediaminetetraacetic acid) precipitation protocol (Applied Biosystems, 2010), and diluted in 10 µL HiDi formamide (Thermo Fisher Scientific). The samples were separated on an ABI Prism 3130XL Genetic Analyzer (Applied Biosystems). Amplification and sequencing of *H. azteca* DNA samples was conducted by AIM - Advanced Identification Methods GmbH (Leipzig, Germany).

Genotyping using microsatellite markers

For microsatellite analysis, 16 markers (Gergs et al., 2010; Švara et al., 2019; Westram et al., 2010) were amplified and genotyped on 60 *G. pulex* DNA samples following the protocol described in Švara et al. (2019) and (Schuelke, 2000). ABI Prism 3130XL Genetic Analyzer was used to separate the samples. Genotyping was done in Genemapper 4.0 (Applied Biosystems). The genetic variation of *G. pulex* was investigated with COI sequence and microsatellite analyses to examine whether distinct genotypes can be differentiated.

Genetic variation analysis

COI sequence data

COI sequence reads were assembled and edited in Sequencher 5.4.5, with gaps coded as (-). Sequence contigs and, as reference, sequences of *G. pulex* from the Holtemme River (see Švara et al., 2019) and other European rivers acquired from National Centre for Biotechnology Information (NCBI) ZKT075230.1_G_pulex_E, KT075232.1_G_pulex_D, KT075256.1_G_pulex_C, KF521835.1_G_fossarum were aligned using ClustalW in MEGA7 (Kumar et al., 2008) with default settings for alignment. Based on the alignment, maximum likelihood tree with best fitting Tamura 3-parameter model and nearest-neighbour-interchange tree Inference method (Tamura et al., 2011) were constructed. The percentage concordance was calculated with 1000 bootstrap iterations. The phylogenetic tree was visualized with FigTree v1.4.3 (http://tree.bio.ed.ac.uk/software/figtree/).

Microsatellite data

The population differentiation was tested calculating the F_{st} value assigning 10000 permutations and p-value of significance at 0.05 in hierfstat package in R. The data distribution, including reference *G. pulex* microsatellite data from the Holtemme River (see Švara et al. 2019), was visualized using PCA analysis from ade4 package in R.

Principal component analysis

The principal component analysis of the analysed specimens of *G. pulex* in comparison with *G. pulex* clade E is presented in Fig. S1.

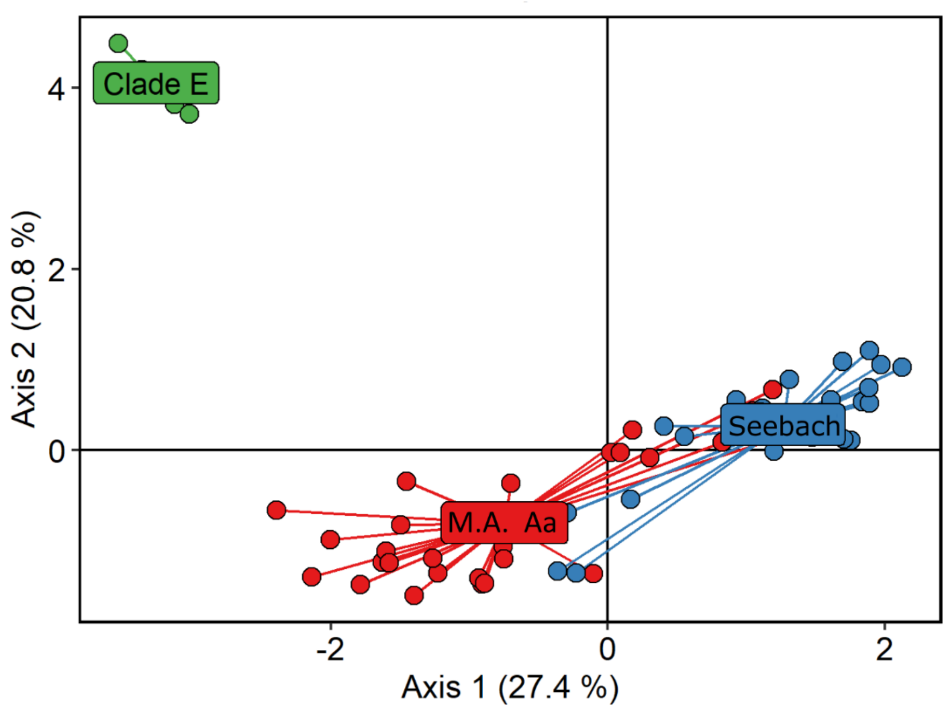

Fig. S1: PCA plot of genotyped individuals from Mönchaltdorfer Aa (M.A. Aa), which were used for toxicokinetic experiments in the present study, and Seebach, which were used by Lauper et al. (2021). Both are plotted against ZKT075230.1_G_pulex_E (Clade E). The first two axes cover 27.4% and 20.8 % of the whole variation.

Summary of the genetic specification results

Characterisation of the analysed species indicated that *H. azteca* cultured in our lab belonged to a *H. azteca* clade originating from Florida, with a 100% sequence match to sequences JX446314, JX446315, JX446313 acquired from National Centre for Biotechnology Information (NCBI; Major et al., 2013). According to the matches with NCBI sequences MF458710 and JF965940, *G. pulex* used in our analyses belongs to a clade distributed north of the Alps in eastern France, Switzerland and to Regensburg in Germany. The amphipods used here form a distinct population with a significant fixation index F_{st} of 0.29 in comparison to *G. pulex* from another analysed location in Switzerland (Müsterlingen, used in Lauper et al., 2021).

SI A3: Test compounds

Tab. S1: Physico-chemical properties and modes of action of the selected test-compounds derived from https://pubchem.ncbi.nlm.nih.gov/. Log D_{ow} is the Log K_{ow} adjusted to the pH of interest (pH 7.9). The log D_{ow} was derived from https://chemicalize.com/ using a QSAR analysis that calculates the octanol-water partitioning coefficients at pH 7.9. Available acute LC_{50} are shown for G. pulex or other crustaceans in brackets.

Compound	CAS	Molecular formula	Molecular weight	Class	MoA (Use)	log Kow	log Dow	pKa	Charge at pH 7.9	Acute LC_{50} [µg/L] (reference)
**Atenolol**	29122–68–7	$C_{14}H_{22}N_2O_3$	266.3	Pharmaceutical	Beta blocker (Blood pressure)	0.2	-1.3	9.6	cation	> mg/L Own observation
**Azoxystrobin**	131860–33–8	$C_{22}H_{17}N_3O_5$	403.4	Fungicide	Mitochondrial respiration inhibitor	2.5	2.5	n.d.	neutral	160 (Rösch et al., 2017)
**Benzotriazole**	95–14–7	$C_6H_5N_3$	119.1	Industrial/ household	Corrosion inhibitor	1.4	1.2	8.4	neutral	107000 (*Daphnia*) (Seeland et al., 2012)
**Carbamazepine**	298–46–4	$C_{15}H_{12}N_2O$	236.3	Pharmaceutical	Voltage-gated Sodium channels (anti-epileptic)	2.3	2.3	n.d.	neutral	> mg/L Own observation
**Citalopram**	59729–33–8	$C_{20}H_{21}FN_2O$	324.4	Pharmaceutical	Sel. serotonin reuptake inhibitor (Anti-depressant)	3.5	1.9	9.8	cation	3900 (Christensen et al., 2007)
**Cyprodinil**	121552–61–2	$C_{14}H_{15}N_3$	225.3	Fungicide	Inhibits protein synthesis	4.0	4.0	4.4	neutral	3000 (Ashauer et al, 2011)
**Diclofenac**	15307–86–5	$C_{14}H_{11}Cl_2NO_2$	296.1	Pharmaceutical	Cyclooxygenase inhibitor (Anti-inflammatory)	4.5	0.9	4.2	anion	216000 (Fu et al., 2020)
**Fluopyram**	658066-35-4	$C_{16}H_{11}ClF_6N_2O$	396.7	Fungicide	Succinate dehydrogenase inhibitor	3.3	3.3	n.d.	neutral	> mg/L Own observation
**Sulfamethoxazole**	723-46-6	$C_{10}H_{11}N_3O_3S$	253.3	Pharmaceutical	PABA competitor (Antibiotic)	0.8	-0.1	1.6	anion	> mg/L Own observation
**Tebuconazole**	107534–96–3	$C_{16}H_{22}ClN_3O$	307.8	Fungicide	Sterol biosynthesis inhibitor	3.7	3.7	5.0	neutral	1600 (*G. fossarum*) (Zubrod et al., 2015)
**Terbutryn**	886–50–0	$C_{10}H_{19}N_5S$	241.4	Herbicide	PSII inhibitor	3.7	3.7	4.3	neutral	2700 (*Daphnia*) (PPDB, 2020)
**Thiacloprid**	111988–49–9	$C_{10}H_9ClN_4S$	252.7	Insecticide	nAChR binding	1.3	1.3	n.d.	neutral	>9500 (Beketov & Liess, 2008)

SI A4: Wet weight – dry weight conversion factors

For the determination of wet weight – dry weight ratios, the wet weight of specimens was determined before specimens were euthanized with ethanol in pre weighted glass vials. The dry weight was determined after drying completely at 60°C over night. Conversion factors were determined as the ratio of wet weight to dry weight. The conversion factors are provided in Tab. S2.

Tab. S2: Experimentally determined wet and dry weight conversion factors for G. pulex and H. azteca.

Species	Wet/dry weight ratio	SD	n
G. pulex	5.4	0.3	3
H. azteca	3.7	0.3	32

SI A5: Chemical analysis

Online SPE LC-HRMS/MS settings

To prepare the online SPE column, 8-9 mg of Oasis HLB (15 µm particle size, Waters) were added to an empty stainless steel SPE cartridge (20 mm x 2.1 mm, BGB Analytik AG). Next, the cartridge was filled with about 8-9 mg of a mix of anion exchanger Strata X-AW, cation exchanger Strata X-CW (both ion exchangers: 30 µm, Phenomenex, UK) and Env+ (70 µm, Biotage, Sweden) in a ratio of 1:1:1.5 (X-AW : X-CW : Env+).

Tab. S3: Schedule of the online-SPE.

Time [min]	Acetonitrile [µL min-1]	Ammonium acetate solution (2 mM) [µL min-1]	SPE step
**0**		200	Elution of the sample from the cartridge (with elution pump) and washing of the loop.
**0.1**	4000		
**1.1**	4000		
**1.2**		4000	
**6.7**		4000	
**6.8**		400	
**7.3**		400	
**7.4**	400		Loading of the new sample into the loop and conditioning the cartridge.
**12.5**	400		
**12.6**		400	
**18.4**		400	
**18.5**		1270	Enrichment of the new sample.
**32.1**		1270	
**34.5**		1270	
**34.7**		1270	
**35**		200	

Tab. S4: Schedule of the liquid chromatography. Water and methanol were both acidified with 0.1% (vol.) formic acid. Chromatographic separation was performed with a reversed-phase column (Atlantis T3 C18 column, 5 µm, 3x150 mm, Waters, Batch No 0151351351)

Time [min]	H_2O [µL min^{-1}]	MeOH [µL min^{-1}]
**0.0**	0.260	0.040
**5.0**	0.260	0.040
**20.0**	0.015	0.285
**29.0**	0.015	0.285
**29.5**	0.260	0.040
**35.0**	0.260	0.040

*Tab. S5: Source parameters used for HRMS/MS measurement with the Q Exactive mass spectrometer. *External mass calibration with an in-house prepared amino acid solution (11 amino acids with m/z between 116 and 997) in positive and negative ionization mode.*

Parameter	Value
**Sheath gas (nitrogen) flow rate**	40 L min^{-1}
**Auxiliary gas (nitrogen) flow rate**	15 L min^{-1}
**Capillary temperature**	350 °C
**S-lens RF level**	50
**Mass calibration**	External*
**Spray voltage**	4 kV (positive ionization mode)
	3 kV (negative ionization mode)

Tab. S6: MS parameters used for HRMS/MS measurement with the Q Exactive mass spectrometer.

Parameter	Value
**Resolution**	70k
**Scan range**	100-1000 m/z
**Polarity**	Switching mode
**Resolution MS2**	17.5k

Analytical quality parameters

Tab. S7: Analytical quality parameters of the quantified parent compounds. ISTD = internal standard. All ISTDs were deuterated analogues of the target analyte.

	**Matrix factor**		**Relative recovery [%]**		**LOQ [µg/kg]**		
**Compound (ISTD)**	Gammarus	Hyalella	Gammarus	Hyalella	Gammarus	Hyalella	Quantification
**Atenolol (Atenolol-d7)**	0.49	0.86	113	97	1.5	4.3	Reference standard and internal standard
**Azoxystrobin (Azoxystrobin-d4)**	1.01	1.34	104	96	1.2	2.7	Reference standard and internal standard
**Benzotriazole (Benzotriazole-d4)**	0.52	0.88	100	95	6.4	18	Reference standard and internal standard
**Carbamazepine (Carbamazepine-d8)**	0.54	0.95	114	100	1.6	4.8	Reference standard and internal standard
**Citalopram (Citalopram-d6)**	1.13	1.61	92	93	12	27	Reference standard and internal standard
**Cyprodinil (Cyprodinil-d5)**	0.85	1.07	103	97	5.2	11	Reference standard and internal standard
**Diclofenac (Diclofenacd-4)**	0.56	0.53	98	97	3.5	5.3	Reference standard and internal standard
**Fluopyram (Fluopyram-d4)**	0.81	1.14	98	94	2.5	5.7	Reference standard and internal standard
**Sulfamethoxazole (Sulfamethoxazole-d4)**	0.49	1.02	101	98	0.5	1.8	Reference standard and internal standard
**Tebuconazole (Tebuconazole-d9)**	0.78	0.85	83	99	2.4	4.3	Reference standard and internal standard
**Terbutryn (Terbutryn-d5)**	0.97	1.23	110	91	12	25	Reference standard and internal standard
**Thiacloprid (Thiacloprid-d4)**	0.32	0.96	105	101	0.2	1.0	Reference standard and internal standard

Tab. S8: Analytical quality parameters of the quantified biotransformation products (BTPs). ISTD = internal standard. AT = atenolol; AZ = azoxystrobin; CMZ = carbamazepine; CIT = citalopram; CY = cyprodinil; DCF = diclofenac, TEB = tebuconazole; TER = terbutryn. Further details on the compound identification without reference standard using HRMS/MS date are described by Fu et al. (2018) (azoxystrobin), Rösch et al. (2016) (tebuconazole), Jeon et al. (2013) (terbutryn) and for cyprodinil below (Fig. S2 and Fig. S3). Terbutryn BTPs were quantified based on the calibration and recovery of TER_M214 (Irgarol-descyclopropyl), due to more similar retention times and higher ionisation efficiencies than the parent compound as applied earlier (Kosfeld et al., 2020). Concentrations of BTPs with recoveries of less than 80 % or more than 120 % were recovery corrected.

	**Matrix factor**		**Relative recovery [%]**		**LOQs [µg/kg]**		
**Parent shortcut_BTP name**	Gammarus	Hyalella	Gammarus	Hyalella	Gammarus	Hyalella	(Semi-)Quantification
**AT_Atenolol acid/Metoprolol acid**	0.64	0.87	150	99	0.5	1.1	Reference standard and ISTD of parent
**AZ_M378**	as AZ	as AZ	as AZ	as AZ	as AZ	as AZ	Based on parent
**AZ_M392**	as AZ	as AZ	as AZ	as AZ	as AZ	as AZ	Based on parent
**AZ_M638 (H)**	as AZ	as AZ	as AZ	as AZ	as AZ	as AZ	Based on parent
**AZ_Azoxystrobin acid**	0.92	1.23	120	114	2.8	6.2	Reference standard and ISTD of parent
**CMZ_Carbamazepine-10-11-epoxide**	0.52	1.06	127	126	0.3	1.1	Reference standard and ISTD of parent
**CIT_Citalopram N-desmethyl**	0.77	1.36	65	81	2.4	6.9	Reference standard and ISTD of parent
**CIT_Citalopram N-oxide**	0.42	0.93	28	41	0.1	0.5	Reference standard and ISTD of parent
**CIT_Citalopram-didesmethyl**	0.86	1.13	48	65	5.3	11.4	Reference standard and ISTD of parent
**CY_M242a (CY_CGA_304075)**	as CY	as CY	as CY	as CY	as CY	as CY	Based on parent
**CY_M242b**	as CY	as CY	as CY	as CY	as CY	as CY	Based on parent
**DCF_M310.0396 (DCF methyl esther)**	0.60	0.62	83	90	1.4	2.4	Reference standard and ISTD of parent
**TEB_M324a**	as TEB	as TEB	as TEB	as TEB	as TEB	as TEB	Based on parent
**TER_M214 (Irgarol-descyclopropyl)**	0.57	0.98	66	74	0.4	1.1	Reference standard and ISTD of parent
**TER_M258a**	as M214	as M214	as M214	as M214	as M214	as M214	Based on TER_M214 (Irgarol-descyclopropyl)
**TER_M258b**	as M214	as M214	as M214	as M214	as M214	as M214	Based on TER_M214 (Irgarol-descyclopropyl)
**TER_M272**	as M214	as M214	as M214	as M214	as M214	as M214	Based on TER_M214 (Irgarol-descyclopropyl)
**TER_M315a**	as M214	as M214	as M214	as M214	as M214	as M214	Based on TER_M214 (Irgarol-descyclopropyl)
**TER_M315b**	as M214	as M214	as M214	as M214	as M214	as M214	Based on TER_M214 (Irgarol-descyclopropyl)

The hydroxylation BTPs of cyprodinil (M242a and M242b) were compared with reference standards of CY CGA 275535, CGA 304075 and CGA 304076 obtained after the analysis. Thus, the reference standards could not be used for quantification. The CY_M242 reference standards were requested through the “ECPA Analytical Standards Request Form” by CropLife Europe, aisbl (https://croplifeeurope.eu/pre-market-resources/analytical-standards-reference-standard-compounds-for-water-monitoring-programmes/) and eventually provided free of charge by Syngenta. The comparison of a spiked and unspiked gammarid sample is presented in Fig. S2, which led to the identification of CY_M242a as CGA 304075. The BTP with no corresponding reference standard (CY_M242b) must be one of the phenol BTPs (Fig. S3), as it showed the same characteristic MS2 fingerprint as CGA 275535 and CGA 304075. The relative recoveries of all spiked reference standards were between 80 and 120 %, thus no correction of the calculated CY M242 concentrations was applied.

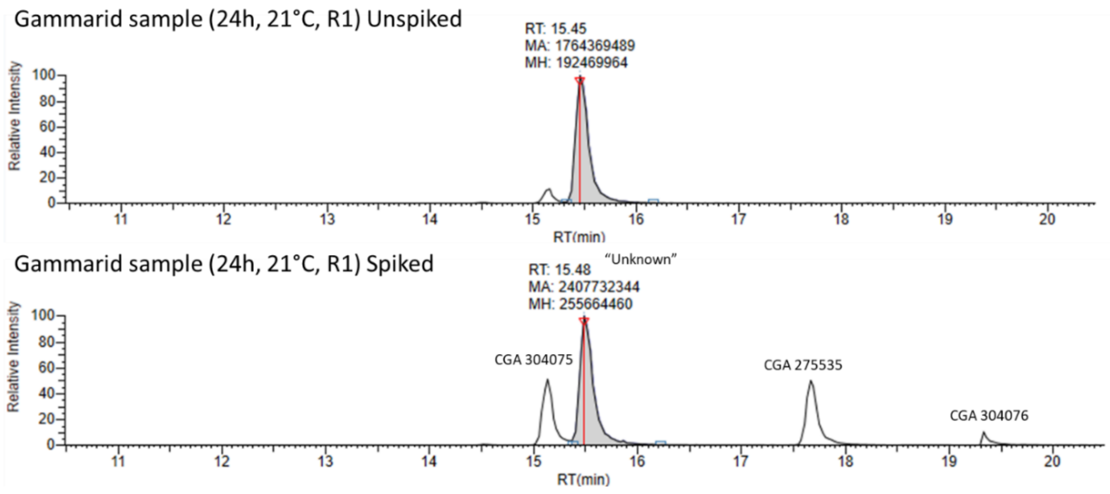

Fig. S2: Chromatogram of cyprodinil BTP spiked and unspiked gammarid sample. One of the two in gammarids and hyalella found hydroxylation BTPs (CY_M242a) could be identified as CGA 304075. The other one remained unidentified.

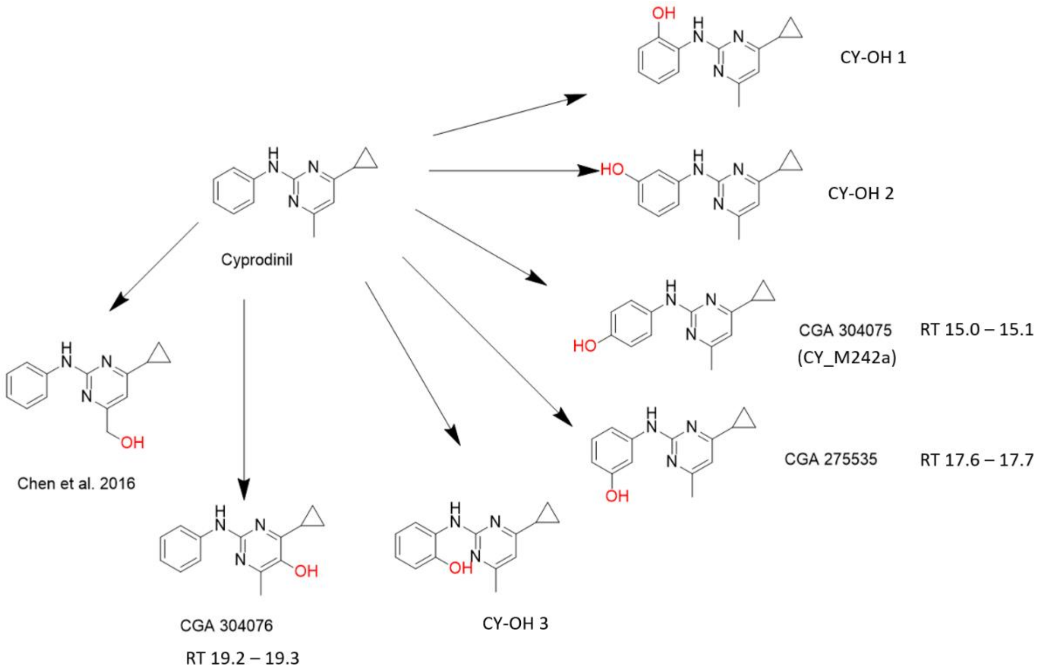

Fig. S3: Overview of the possible hydroxylation BTPs of cyprodinil (= CYM242). The unidentified (CY_M242b) BTP is one of the phenol BTPs (CY_OH 1 to 3).

Biotransformation product evaluation

Tab. S9: Inclusion list and detection of BTPs. Green shading: parent compound with reference standard in the calibration and ISTD; yellow shading: BTP with reference standard in the calibration; red shading: BTP with no reference standard. Mode = ionisation mode. G = G. pulex; H = H. azteca; Cal = calibration; Q = quantified. 1 (green) = compound detected in at least one sample; 0 (red) = compound not detected; 0.5 (orange) = compound detected (i.e. in calibration) but should not be present (potentially formed from parent compound or impurity). Such compounds were excluded from quantification and further analysis. y = yes (quantified); n = no (not quantified). BTPs with too little intensities were not quantified. References for detection of the BTPs in environmental matrices can be used for further details on the BTP identification. Some BTPs were added to the list, because reference standards were available.

**Compound**	**Molecular Formula**	**Mode**	**$[M+H]^+$ $[M-H]^-$**	G	H	Cal	Q	Reference/Reason
Atenolol	C14H22N2O3	pos	267.1703	1	1	1	y	
Atenolol Acid	C14H21NO4	pos	268.1543	1	1	1	y	Standard available
Atenolol-desisopropyl	C11H16N2O3	pos	211.1203	0	0	1	n	Standard available
Azoxystrobin	C22H17N3O5	pos	404.1241	1	1	1	y	
AZ_M214	C11H7N3O2	pos	214.0611	1	1	0	n	(Fu et al., 2018)
AZ_M362a	C20H15N3O4	pos	362.1135	1	1	0	y	(Fu et al., 2018)
AZ_M362b	C20H15N3O4	pos	362.1135	1	1	0	y	(Fu et al., 2018)
AZ_M378	C20H15N3O5	pos	378.1084	1	1	0	y	(Fu et al., 2018)
AZ_M390a	C21H15N3O5	pos	390.1084	1	1	0	n	(Fu et al., 2018)
AZ_M390b (Azoxystobin Acid)	C21H15N3O5	pos	390.1084	1	1	1	y	(Fu et al., 2018)
AZ_M392	C21H17N3O5	pos	392.1241	1	1	0	y	(Fu et al., 2018)
AZ_M420	C22H17N3O6	pos	420.1190	1	0	0.5	n	(Fu et al., 2018)
AZ_M436 (H)	C22H17N3O7	pos	436.1139	0	0	0	n	(Fu et al., 2018)
AZ_M485 (H)	C22H20N4O7S	pos	485.1125	0	0	0	n	(Fu et al., 2018)
AZ_M493	C24H20N4O6S	pos	493.1176	1	0	0	y	(Fu et al., 2018)
AZ_M497 (H)	C23H20N4O7S	pos	497.1125	0	1	0	n	(Fu et al., 2018)
AZ_M498	C22H17N3O9S	pos	498.0613	0	0	0	n	(Fu et al., 2018)
AZ_M513 (H)	C23H20N4O8S	pos	513.1074	0	0	0.5	n	(Fu et al., 2018)
AZ_M514	C22H17N3O10S	pos	514.0562	0	0	0	n	(Fu et al., 2018)
AZ_M525	C25H24N4O7S	pos	525.1438	1	0	0	n	(Fu et al., 2018)
AZ_M541 (H)	C25H24N4O8S	pos	541.1388	1	1	0	n	(Fu et al., 2018)
AZ_M552 (AZ glucosidation Peak1+2)	C27H25N3O10	pos	552.1613	1	1	0	n	(Fu et al., 2018)
AZ_M554a (H)	C27H27N3O10	pos	554.1769	0	1	0.5	n	(Fu et al., 2018)
AZ_M582a (H)	C28H27N3O11	pos	582.1718	0	0	0	n	(Fu et al., 2018)
AZ_M618	C26H25N3O13S	pos	618.1035	0	0	0	n	(Fu et al., 2018)
AZ_M630	C27H25N3O13S	pos	630.1035	0	0	0	n	(Fu et al., 2018)
AZ_M632 (H)	C27H27N3O13S	pos	632.1192	0	0	0	n	(Fu et al., 2018)
AZ_M638 (H)	C30H27O13N3	pos	638.1617	0	1	0	y	(Fu et al., 2018)
AZ_M640 (H)	C30H29O13N3	pos	640.1773	0	1	0	y	(Fu et al., 2018)
AZ_M660 (H‡)	C28H27O14N3S	pos	660.1141	0	0	0	n	(Fu et al., 2018)
AZ_M684 (H)	C31H29O15N3	pos	684.1671	0	0	0	n	(Fu et al., 2018)
AZn_2-hydroxybenzonitrile (2HBzN)	C7H5NO	neg	118.0293	0	0	0	n	(Fu et al., 2018)
Benzotriazole	C6H5N3	pos	120.0556	1	1	1	y	
BTX_N-sulfate	C6H5N3O3S	pos	200.0124	0	0	0	n	(Damalas et al., 2018)

BTX_O-sulfate	C6H5N3O4S	pos	216.0074	0	0	0	n	(Damalas et al., 2018)
BTX_Nglucuronide	C12H8N3O6	pos	291.0486	0	0	0	n	(Damalas et al., 2018)
BTX_5-Methyl-1H-benzotriazole	C7H7N3	pos	134.0713	0	0	1	n	Standard available
BTX_Dimethyl-Benzotriazole	C8H9N3	pos	148.0869	0	0	1	n	Standard available
BTX_5-OH	C6H6N3O	pos	137.0584	0	0	0	n	(Huntscha et al., 2014)
BTX_4-OH	C6H6N3O	pos	137.0584	0	0	0	n	Huntscha et al., 2014)
BTX_1-Hydroxy	C6H5N3O	pos	136.0505	0	0	1	n	Huntscha et al., 2014)
Carbamazepine	C15H12N2O	pos	237.1022	1	1	1	y	
CMZ_dihydro	C15H14N2O1	pos	239.1179	0	1	1	n	Standard available
CMZ_dihydro-dihydroxy	C15H14N2O3	pos	271.1077	0	0	1	n	Standard available
CMZ_Oxcarbazepine	C15H12N2O2	pos	253.0972	0	0	1	n	Standard available
CMZ_Iminostilben	C14H11N1	pos	194.0964	0	0	1	n	Standard available
CMZ_epoxid	C15H12N2O2	pos	253.0972	1	1	1	y	Standard available
Citalopram	C20H21FN2O	pos	325.1711	1	1	1	y	
CIT N-desmethyl	C19H19FN2O	pos	311.1554	1	1	1	y	Standard available
CIT N-oxide	C20H23N2O2F1	pos	341.1660	1	1	1	y	Standard available
CIT didesmethyl	C20H21FN2O2	pos	297.1398	1	1	1	y	Standard available
Cyprodinil	C18H17FN2O	pos	226.1339	1	1	1	y	
CY-TP CGA 249287	C8H11N3	pos	150.1026	0	0	1	n	(Kiefer et al., 2019)
CY_M378	C17H19N3O5S	pos	378.1118	0	0	0	n	(Sapp et al., 2004)
CY_M362	C17H19N3O4S	pos	362.1169	0	0	0	n	(Sapp et al., 2004)
CY_M242a (CY_CGA_304075)	C14H15N3O	pos	242.1288	1	1	0	y	(Sapp et al., 2004)
CY_M242b, c, d, e	C14H15N3O	pos	242.1288	1	1	0	y	(Sapp et al., 2004)
CY_M345	C17H20N4O2S	pos	345.1380	0	0	0	n	(Sapp et al., 2004)
CY_M240	C14H13N3O	pos	240.1131	0	1	0	n	(Sapp et al., 2004)
CY_M151	C8H10N2O	pos	151.0866	0	0	0	n	(Sapp et al., 2004)
CY_M136	C7H9N3	pos	136.0869	0	0	0	n	(Sapp et al., 2004)
Diclofenac	C14H11Cl2NO2	pos	296.0240	1	1	1	y	
DCF_M310.03 (Diclofenacmethyl ester)	C15H13Cl2NO2	pos	310.0396	1	1	1	y	(Fu et al., 2020)
DCF-M403 (Diclofenac taurine)	C16H16Cl2N2O4S	pos	403.0282	0	0	1	n	(Fu et al., 2020)
DCF_M310.003	C14H9Cl2NO3	pos	310.0032	1	0	0	n	(Fu et al., 2020)
DCF_M294	C14H9Cl2NO2	pos	294.0083	1	0	0	n	(Fu et al., 2020)
DCF_M312	C14H11Cl2NO3	pos	312.0188	1	0	0	n	(Fu et al., 2020)
DCF_M324	C15H11Cl2NO3	pos	324.0192	0	0	0	n	(Fu et al., 2020)
DCF_M326	C15H13Cl2NO3	pos	326.0348	0	0	0	n	(Fu et al., 2020)
DCF_M371	C14H9Cl2NO5S	pos	371.9492	0	0	0	n	(Fu et al., 2020)
DCF_M417	C16H16Cl2N2O5S	pos	417.0086	0	0	0	n	(Fu et al., 2020)
DCF_M538	C20H21Cl2NO10S	pos	538.0335	0	0	0.5	n	(Fu et al., 2020)
Fluopyram	C16H11ClF6N2O	pos	397.0537	1	1	1	y	
FLU_TMB (Fluopyram Benzamide)	C8H6F3NO	pos	190.0474	1	1	1	n	(Vargas-Pérez et al., 2020)

PCA (3-Chloro-5-(trifluoromethyl)picolinic acid) (or TPA, Wie 2016)	C7H3ClF3NO2	neg	225.9877	0	0	1	n	(Vargas-Pérez et al., 2020)
FLU_TPAA (Fluopyram-PAA in Vargas)	C8H5ClF3NO2	pos	240.0034	0	1	0	n	(Vargas-Pérez et al., 2020)
FLU-7-OH /8-OH	C16H11ClF6N2O2	pos	413.0486	1	1	0	n	(Vargas-Pérez et al., 2020)
FLU_2,9-bis(trifluoromethyl)-6,7-dihydropyrido[2,3-e][2]benzazocin-8(5H)-one	C16H10F6N2O	pos	361.0770	0	0	0	n	(Vargas-Pérez et al., 2020)
FLU-OH	C16H9ClF6N2O2	pos	411.0329	0	0	0	n	(Vargas-Pérez et al., 2020)
FLU-OH-GA	C22H19ClF6N2O8	pos	589.0807	0	0	0	n	(Vargas-Pérez et al., 2020)
FLU-OH-glc	C22H21ClF6N2O7	pos	575.1014	0	0	0	n	(Vargas-Pérez et al., 2020)
FLU-OH-glc-MA	C25H23ClF6N2O10	pos	661.1018	0	0	0	n	(Vargas-Pérez et al., 2020)
FLU-OH-SA	C16H11ClF6N2O5S	pos	493.0054	0	0	0	n	(Vargas-Pérez et al., 2020)
FLU-olefin	C16H9ClF6N2O	pos	395.0381	0	0	0	n	(Vargas-Pérez et al., 2020)
FLU-pic	C7H5ClF3N	pos	196.0136	0	0	0	n	(Vargas-Pérez et al., 2020)
FLUn-benzoic acid	C8H5F3O2	neg	189.0163	0	0	0	n	(Vargas-Pérez et al., 2020)
FLUn-methyl-sulfoxide	C8H6SNO3	neg	291.0204	0	0	0	n	(Vargas-Pérez et al., 2020)
FLUn_2,9-bis(trifluoromethyl)-6,7-dihydropyrido[2,3-e][2]benzazocin-8(5H)-one	C16H10F6N2O	neg	359.0619	0	0	0	n	(Vargas-Pérez et al., 2020)
Tebuconazole	C16H22ClN3O	pos	308.1524	1	1	1	y	
TEB_COOH	C16H22ClN3O3	pos	340.1422	1	1	0	n	(Rösch et al., 2016)
TEB_M324a (TEB OH)	C16H22ClN3O2	pos	324.1473	1	1	0	y	(Rösch et al., 2016)
TEB_M388	C16H23ClN3O4P	pos	388.1187	1	1	0	n	(Rösch et al., 2016)
TEB_M404	C16H23ClN3O5P	pos	404.1137	1	1	1	n	(Rösch et al., 2016)
Triazole alanine	C5H8N4O2	neg	155.0569	0	0	1	n	Standard available
Terbutryn	C10H19N5S	pos	242.1434	1	1	1	y	
TER_Irgarol-descyclopropyl	C8H15N5S1	pos	214.1121	1	1	1	y	(Jeon et al., 2013)
TER_MTE258A	C10H20ON5S	pos	258.1384	1	1	0	y	(Jeon et al., 2013)
TER_MTE258B	C10H20ON5S	pos	258.1384	1	1	0.5	y	(Jeon et al., 2013)
TER_MTE272	C10H18O2N5S	pos	272.1174	1	1	0	y	(Jeon et al., 2013)
TER_MTE315A	C12H22N6O2S	pos	315.1602	1	1	0	y	(Jeon et al., 2013)
TER_MTE315B	C12H22N6O2S	pos	315.1602	1	1	0	y	(Jeon et al., 2013)
TER_MTE501	C19H33O6N8S	pos	501.2236	1	1	0	n	(Jeon et al., 2013)
Thiacloprid	C10H9ClN4S	pos	253.0309	1	1	1	y	
Thiacloprid Amide	C10H11N4O1S1Cl1	pos	271.0415	0	0	1	n	(Y.-J. Dai et al., 2010)
THI-NH	C9H10ClN3S	pos	228.0357	0	0	0.5	n	(Ford & Casida, 2008)

THI-ole-NH	C9H9ClN3S	pos	227.0278	0	0	0	n	Ford & Casida, 2008)
THI-4-OH	C10H9ClN4OS	pos	269.0258	0	0	0	n	Ford & Casida, 2008)
THI-NCONH2	C12H9ClN4OS	pos	293.0258	0	0	0	n	Ford & Casida, 2008)
Sulfamethoxazole	C10H11N3O3S	pos	254.0594	1	1	1	y	
N4-Acetylsulfamethoxazole	C12H13N3O4S	neg	294.0549	0	0	1	n	Standard available
Sulfamethoxazole N1-glucuronide	C16H19N3O9S1	neg	428.0764	0	0	1	n	Standard available

SI A6: Determination of lipid and protein contents

Lipid and protein methods

Samples for lipid and protein content analysis were collected as described for the chemical analysis.

Lipid content was determined gravimetrically (Smedes, 1999) following a for Eawag facilities adapted protocol based on Raths et al. (2020). Samples were homogenised using the above ("Sample preparation", main document) described FastPrep bead beater and zirconia/silica beads protocol, but 1 mL of cyclohexane:isopropanol (5:4, v:v). Afterwards, 0.6 mL NPW were added, samples vortexed and centrifuged (1000 g, 10 min, 20°C). The organic phase was transferred into a pre-weighed glass vial. The procedure was repeated after addition of 0.55 mL cyclohexane:isopropanol (87:13, v:v).). The combined extracts were evaporated to dryness at 60°C and the lipid content was determined gravimetrically. Only Eppendorf 2 mL tubes were used for lipid determination, as other tubes appeared to have leachates that influence the evaporated lipid extract weight (Kretschmann et al., 2011).

Total protein content was determined using the Pierce BCA Protein Assay Kit (ThermoScientific) with bicinchoninic acid (Janssen et al., 2012). Briefly, organisms were homogenised after the addition of 300 mg 1 mm zirconia/silica beads using a nitrogen cooled Bead Ruptor Elite (two cycles of 15 s at 6 m s^{-1}; Omni International) at 4°C. After homogenisation, 1 mL of T-PER Reagent (ThermoScientific) was added, samples vortexed and centrifuged (20 000 g, 5 min, 4°C). The supernatant was collected and 1:4 (v:v) diluted in T-PER Reagent. 25 µL of the dilution were incubated for 30 min at 37 °C in the assay working solution in a 96-well plate. Afterwards, absorbance was measured at 562 nm using a microplate reader (Infinite M200, Tecan Trading AG). The results were related to protein concentrations relative to a calibration with albumin standards.

Lipid and protein results

The lipid content in *G. pulex* ranged from 0.9 ± 0.1 to 1.1 ± 0.1% ww (± SD) across the four temperature treatments (Fig. S4). Lipid content in *H. azteca* was two to three times higher and with 2.5 ± 0.3 to 2.9 ± 0.3% ww (± SD). Generally, a significantly ($p < 0.05$) increased lipid content from 6 °C to 21 °C was observed in both species. However, the relative differences between the temperatures were small.

The protein content (Fig. S4) ranged from 1.8 ± 0.4 to 2.1 ± 0.3% ww (± SD) in *G. pulex* and was higher in *H. azteca* with 2.7 ± 0.3 to 3.4 ± 0.2% ww (± SD). The protein content showed no clear temperature related trend in both species, but was significantly lower at 6 and 16°C compared to 11°C and lower at 16°C compared to 21°C in *H. azteca.* However, the relative differences were small. Later, a method by Maloney et al. (2021) was found to consistently extract approximately 25% more proteins from *G. pulex* tissue, thus the present results may underestimate the total protein content. However, the relative difference in the present results may still be comparable.

Lipid contents were in the range of lipid contents (if necessary converted to a ww basis using the conversion factors of Tab. S2) reported elsewhere for *G. pulex* (0.2 to 2.3% ww, Dalhoff et al., 2018; 2.6 ± 0.3% ww, Fu et al., 2018; 0.9 to 1.4% ww, Götz et al., 2021) and *H. azteca* (0.7 to 4.4% ww, Arts et al., 1995; 1.9 ± 0.7% ww, Fu et al., 2018; 1.9 to 3.6% ww, Raths et al., 2020; 1.0 to 4.3% ww Schlechtriem et al., 2019). Protein contents were slightly below protein contents reported for *G. pulex* (2.6 to 8.3 % ww, Dalhoff et al., 2018).

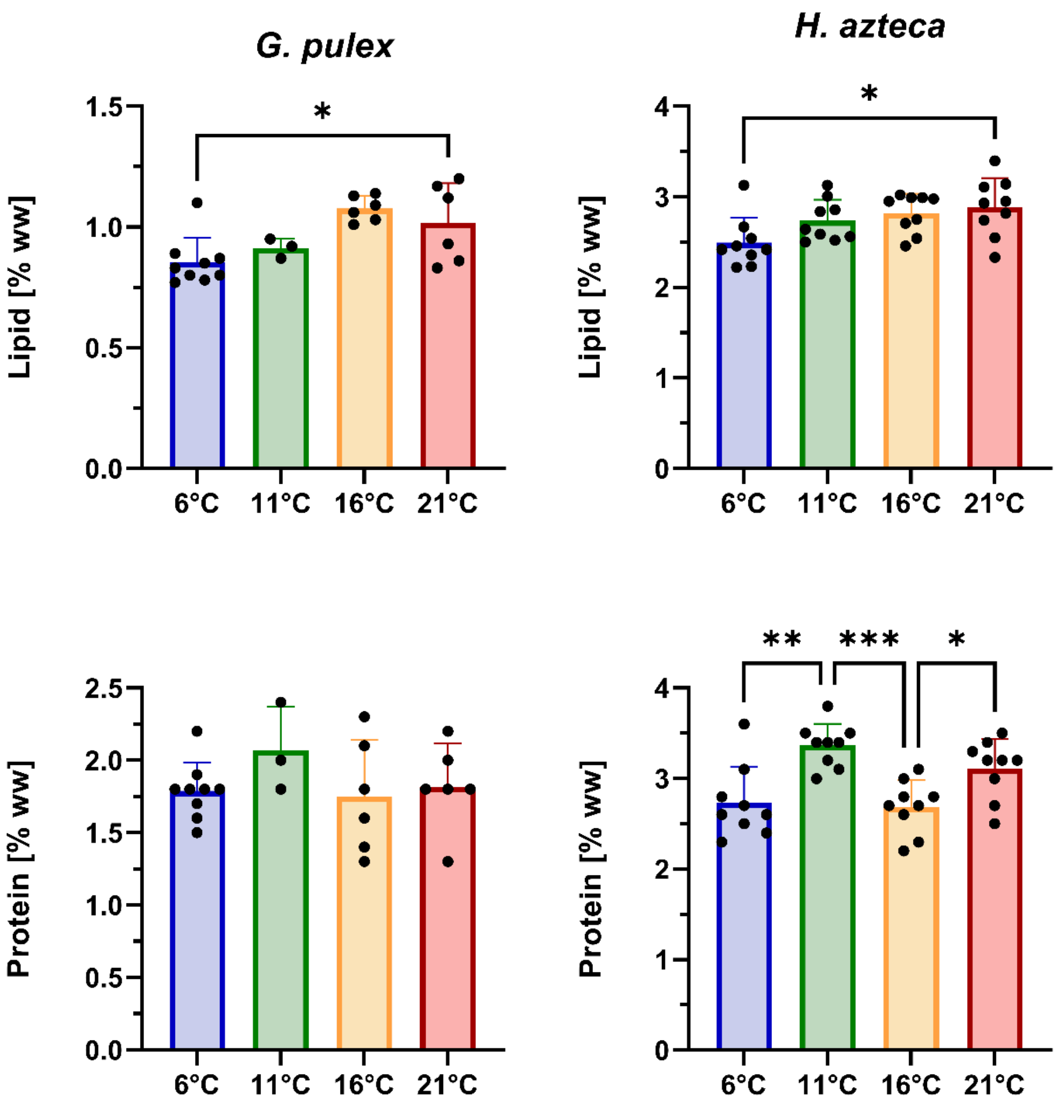

*Fig. S4: Lipid (top) and protein (bottom) contents in the two amphipod species at different temperatures on a wet weight basis. * = p < 0.05; *** = p < 0.001 (two-way ANOVA, Tukey post-hoc).*

SI A7: Calculation of time to 95% steady state t_{ss} and elimination half-life $t_{1/2}$

The following calculations are performed according to Arlos et al. (2020). The time to 95% steady state can be calculated as:

$$t_{ss} = \frac{\ln(0.05)}{-k_e}$$

Alternatively, if biotransformation is considered:

$$t_{ss} = \frac{\ln(0.05)}{-(k_e + k_{m,1st})}$$

The elimination half-life was calculated as following:

$t_{1/2}$ of the parent compound:

$$t_{1/2,p} = \frac{\ln 2}{k_e + k_{m,1st}}$$

$t_{1/2}$ of primary BTPs:

$$t_{1/2,1st} = \frac{\ln 2}{k_{e,1st} + k_{m,2nd}}$$

$t_{1/2}$ of secondary BTPs:

$$t_{1/2,2nd} = \frac{\ln 2}{k_{e,2nd}}$$

For the simple TK models without biotransformation $k_{m,1st}$ would be set to zero.

SI A8: Standard metabolic rate measurements

Standard metabolic rate methods

Standard metabolic rates of *H. azteca* were assessed using a 10-channel respirometer equipped with fibre-optic oxygen mini- sensors (FIBOX 3, PreSens). The respirometry chambers were inset into a hollow aluminium block that was temperature regulated by a circulating water flow originating from a heater/chiller, which held the temperature stable. Individual acclimated specimens of *H. azteca* were placed in the respirometry chambers containing 20 mL medium and an integrated stir bar. For each temperature (6°C, 11°C, 16°C and 21°C) eight replicates and two controls (no animal) were run. Oxygen consumption was determined with a linear regression model based on the dissolved oxygen concentration within a 6 h time window (30 s measurement intervals) of linear oxygen decrease within the 16 h experiment. After the experiment, the exact medium volume was determined gravimetrically and both, wet and dry weight of the specimens determined to the nearest 0.01 mg. The standard metabolic rates (in µg g^{-1} h^{-1}) were calculated by normalising changes in dissolved oxygen over time O_t (µg L^{-1} h^{-1}) in each chamber compared to the average of the controls O_c (µg L^{-1} h^{-1}), divided by the medium volume *V* (L) and the dry weight *dw* (g) of each replicate specimen:

$$SMR = \frac{(O_t - O_c)}{V * dw}$$

Standard metabolic rates results

The obtained standard metabolic rates (dw basis) and exponential fit are presented in Fig. S5. A comparison of the present study with two other studies with *H. azteca* (Mathias, 1971) and mayfly (Camp & Buchwalter, 2016) is presented in Fig. S6. The standard metabolic rates had to be converted to a wet weight basis in order to be comparable with the mayfly larvae data, which were reported on a ww basis only. The Arrhenius relationship of the standard metabolic rates is presented in Fig. S7 and compared to the other respiration rate studies with *H. azteca* (Mathias, 1971) and mayfly (Camp & Buchwalter, 2016) which resulted in very similar Arrhenius temperatures (8030 to 9380 K).

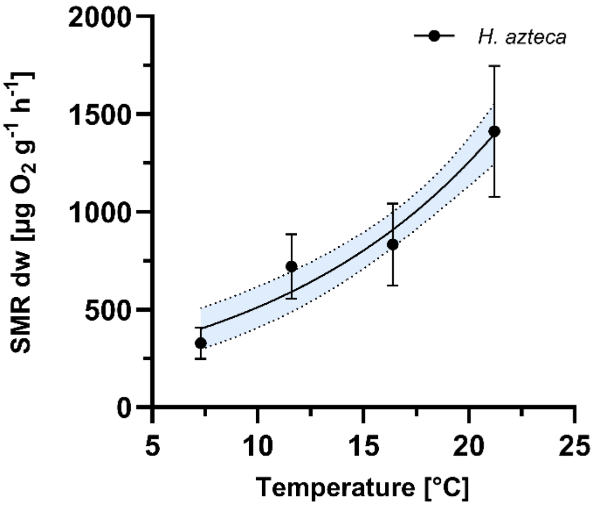

Fig. S5: Standard metabolic rate (dry weight) of H. azteca across the four tested temperatures. Presented mean ± SD (n =8) and exponential growth fit with 95% CIs. SMR = standard metabolic rate.
R^2= 0.75, $SMR = 210 * e^{0.09\,T}$.

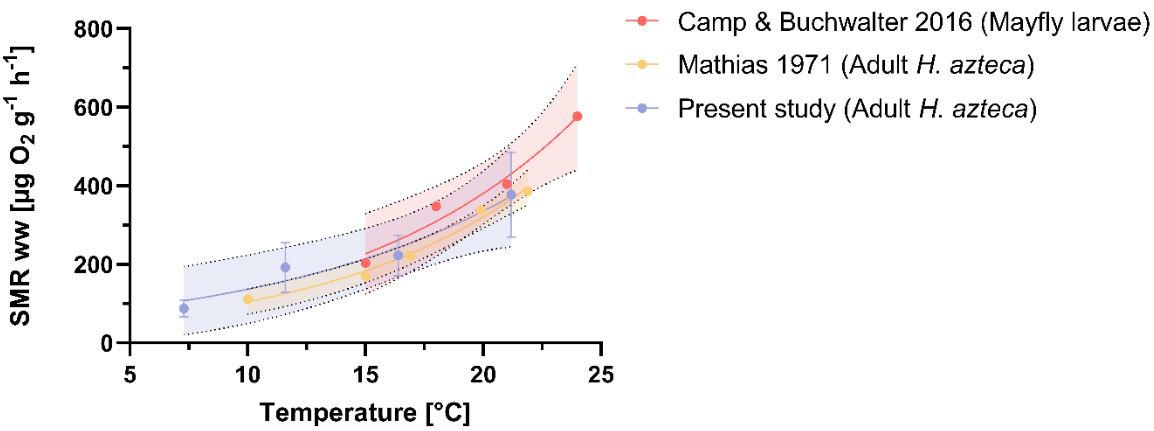

Fig. S6: SMR (ww) for the three compared studies. Presented as mean (present study ± SD, n =8) and exponential fit with 95% CIs. SMR = standard metabolic rate.

Camp & Buchwalter 2016 (Mayfly larvae):	*R^2= 0.97* $SMR = 48 * e^{0.10\,T}$
Mathias 1971 (Adult H. azteca):	*R^2= 0.98* $SMR = 34 * e^{0.11\,T}$
Present study Adult H. azteca	*R^2= 0.95* $SMR = 56 * e^{0.09\,T}$

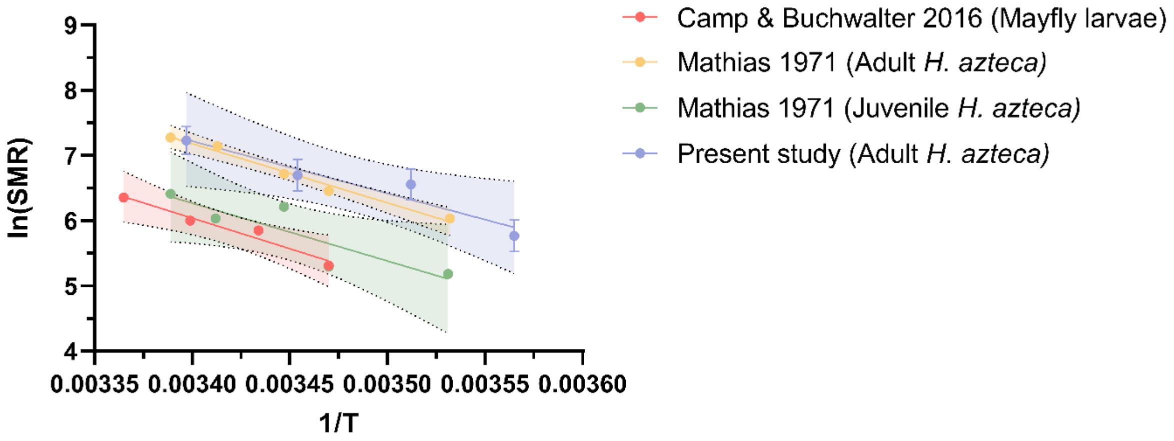

Fig. S7: Arrhenius relationships of the standard metabolic rates. Standard metabolic rates are presented on a dw basis, except for Camp & Buchwalter 2016 which is on a ww basis. Presented as mean (present study ± SD, n =8) and linear fit with 95% CIs. SMR = standard metabolic rate.

Camp & Buchwalter 2016 (Mayfly larvae):	*R^2= 0.96*	*ln(SMR) = -9380*1/T + 38.0*	*$p < 0.05$.*
Mathias 1971 (Adult H. azteca):	*R^2= 0.98*	*ln(SMR) = -9070*1/T + 38.0*	*$p < 0.001$.*
Mathias 1971 (Juvenile H. azteca):	*R^2= 0.77*	*ln(SMR) = -8870*1/T + 36.4*	*$p < 0.05$.*
Present study Adult H. azteca	*R^2= 0.93*	*ln(SMR) = -8030*1/T + 34.5*	*$p < 0.05$.*

SI A9: Pre-test on temperature dependent toxicokinetics and investigations on the influence of organism size and lipid content

Methods: Gammarus pulex

Specimens of *G. pulex* were collected from a small uncontaminated as described in the main manuscript. The water temperature at the time of sampling (11.05.2020) was 14°C. Female gammarids with eggs, as well as obviously parasitized individuals were avoided. Gammarid size for the main experiment was between 1.3 and 1.5 cm (on average 37 mg ww). Further, gammarids with a size of ≤ 0.8 cm (on average 14 mg ww) were collected to assess size effects. Gammarids were acclimated for four days at 16°C in aerated APW in a 8 L tank containing rocks and leaves collected in the field. Afterwards, gammarids were acclimated to the corresponding test temperature and vessels for another 3 days. In this time, they were fed ad libitum with the leaf mix from the field.

Methods: Exposure scenarios

Specimens of *G. pulex* were exposed for up to 24 h to a mix of 14 chemicals (the 12 compounds from Tab. S1, as well as the two cationic surfactants N-methyldodecylamine (S12) and N,N,N-trimethyl-1-tetradecylammonium (Q14) which were not quantified due to high carry over in the online-SPE system), 50 µg/L each, at three different temperatures (6°C, 16°C, 21°C). The day and night rhythm was set to 16:8 h day:night with an intensity of 1200 lux. Artificial pond water (APW), according to Naylor et al. (1989), was used as a test medium. The pH of APW was 7.9. The organism density was four gammarids per 500 mL beaker. Gammarids were not fed during the uptake phase. The exposure scenarios are summarised in Tab. S10.

Tab. S10: Exposure scenarios of the pre-test.

Temperature	6 °C	16 °C	21 °C
**Control 24 h**		X	
**Exposure 6 h**	X	X	X
**Exposure 24 h**	X	X	X
**Exposure 24 h small gammarids**		X	

Methods: Sample analysis

All gammarid samples were taken as triplicates (each consisted of four gammarids = one beaker), shock-frozen in liquid N_2 and stored at -20°C until analysis. At the beginning of the exposure and at the time of sampling (6h or 24h) 500 µL media samples were taken, conserved with 400 µL of methanol and 100 µL internal standard mix and stored at -20°C until analysis.

Lipid content was analysed as described in “SI A6: Determination of lipid and protein contents” in both gammarid size classes (sampled at the end of the experiment at 16°C) as triplicates. Tissue extraction and analysis was performed as described in the main document.

Methods: Bioconcentration

The ratio of internal concentration compared to the medium concentration was calculated after 6 h and 24 h, respectively (BCF_{6h} and BCF_{24h}).

Results: Lipid content

The lipid content in the gammarids ("large") at 16°C after 24 h of exposure was 2.0 ± 0.1%. Lipid content in the smaller gammarid fraction was twice as much with 4.0 ± 0.6 % Fig. S8.

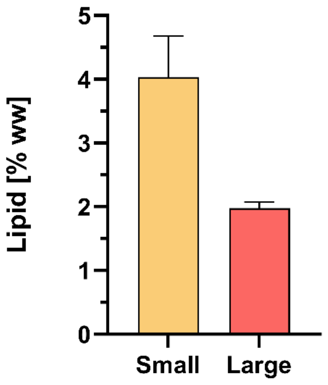

Fig. S8: Lipid content of the two size classes presented as mean ± SD (n = 3).

Results: Bioconcentration – size and lipid effect

The BCF_{24h} for the size comparison is presented in Fig. S9. The BCF_{24h} was slightly higher in the smaller gammarid size class, but no significant difference between the two size classes and lipid contents was detected except for benzotriazole. However, the difference was relatively small and did not correlate to the difference in lipid content. The results indicate that size class has little impact on the BCF. Further, based on the lipid normalisation approach a twofold higher accumulation would have been expected in the smaller gammarid size class, which was not observed. Thus, a lipid normalisation needs to be handled with caution to prevent misinterpretations. The measured lipid contents in *H. azteca* (Fig. S4) were in the range of the tested gammarids, thus we conclude that lipid normalisation would not be appropriate for a BCF comparison of the two species.

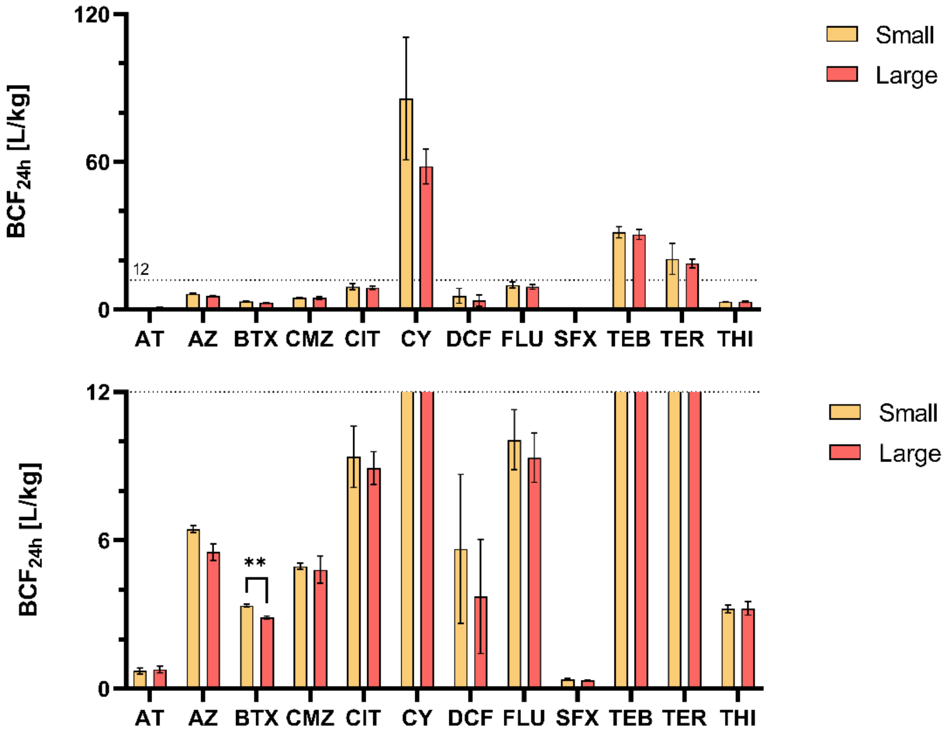

*Fig. S9: BCF_{24h} of the two gammarid size classes at 16°C. Presented as mean ± SD (n = 3). ** = p < 0.01 (t-test). AT = atenolol, AZ = azoxystrobin, BTX = benzotriazole, CIT = citalopram, CMZ = carbamazepine, CY = cyprodinil, DCF = diclofenac, FLU = fluopyram, TEB = tebuconazole, TER = terbutryn, THI = thiacloprid, SFX = sulfamethoxazole.*

Results: Bioconcentration – temperature effect

The BCFs calculated after 6 h (Fig. S10) and 24 h (Fig. S11) at the three different temperatures showed an increasing trend with temperature for most compounds at 6 h indicating a temperature-effect on ku in the non-equilibrium conditions. At 24 h, this trend remained only for citalopram and diclofenac. The BCF_{24h} decreased significantly from 16°C to 21°C for cyprodinil, tebuconazole and terbutryn, which might be explained by metabolic adaption (biotransformation) or the approximation to the physiological temperature limit of *G. pulex*.

A comparison of the pre-test data with the modelled parameters from the main experiment is presented in "SI A11: Confirmation of the modelled toxicokinetic parameters using the pre-test

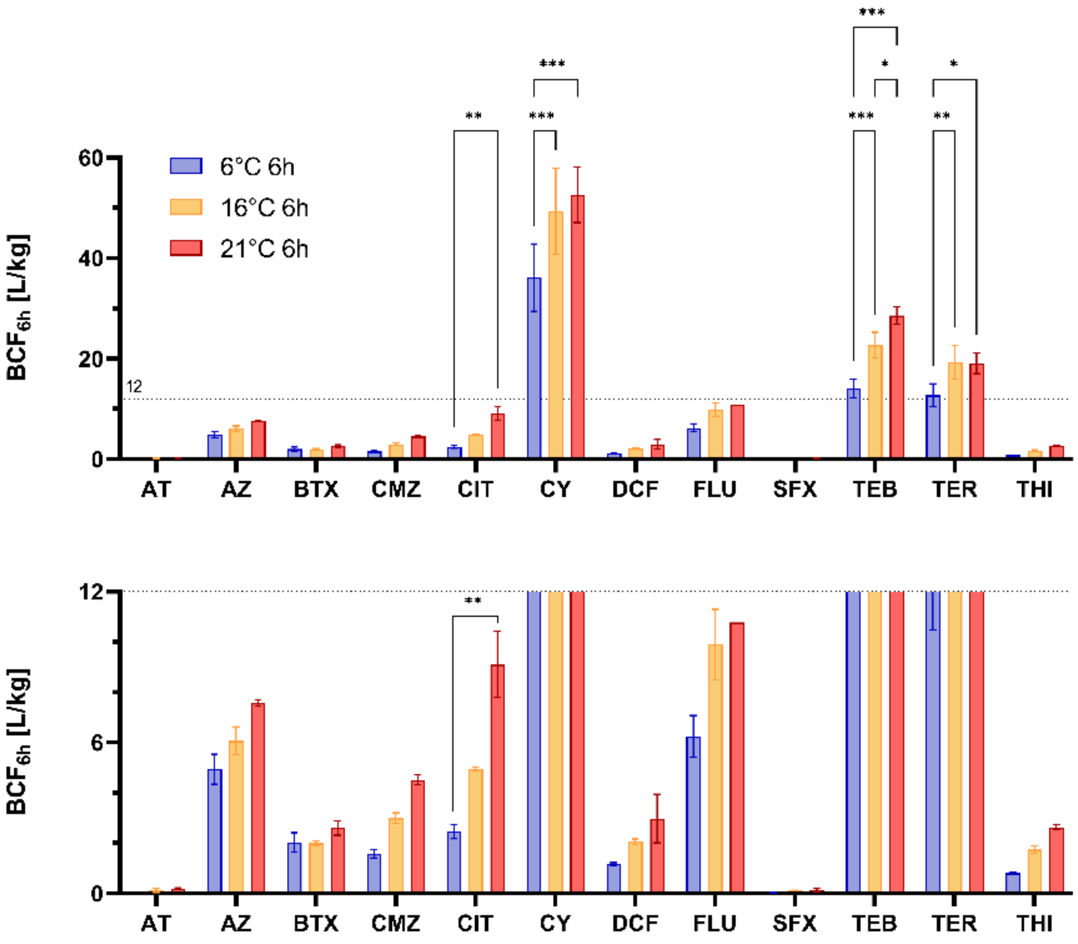

dataset.

*Fig. S10: Calculated BCF_{6h} across the tested temperature range. Presented as mean ± SD (n = 3). * = p < 0.05, * = p < 0.01, *** = p < 0.001 (two-way ANOVA, Tukey post-hoc). AT = atenolol, AZ = azoxystrobin, BTX = benzotriazole, CIT = citalopram, CMZ = carbamazepine, CY = cyprodinil, DCF = diclofenac, FLU = fluopyram, SFX = sulfamethoxazole, TEB = tebuconazole, TER = terbutryn, THI = thiacloprid.*

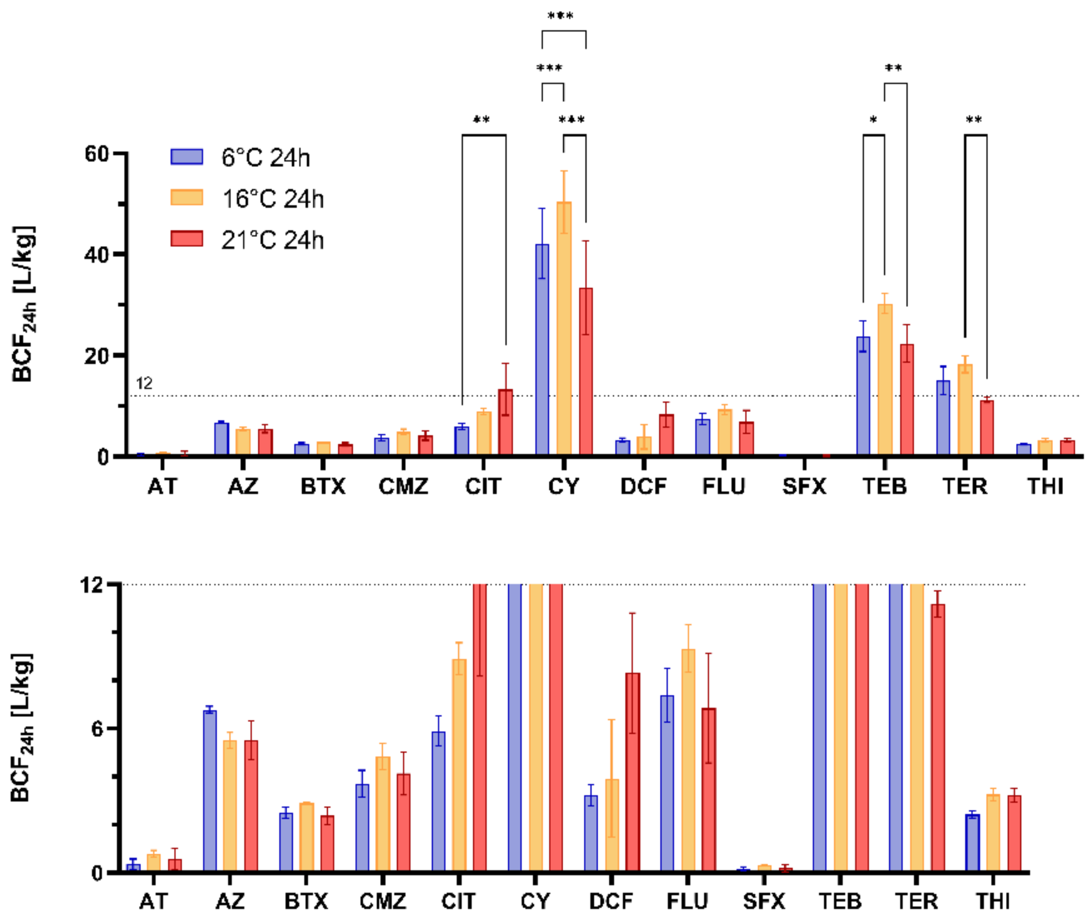

*Fig. S11: Calculated BCF_{24h} across the tested temperature range. Presented as mean ± SD (n = 3). * = p < 0.05, ** = p < 0.01, *** = p < 0.001 (two-way ANOVA, Tukey post-hoc). AT = atenolol, AZ = azoxystrobin, BTX = benzotriazole, CIT = citalopram, CMZ = carbamazepine, CY = cyprodinil, DCF = diclofenac, FLU = fluopyram, SFX = sulfamethoxazole, TEB = tebuconazole, TER = terbutryn, THI = thiacloprid.*

SI A10: Modelled toxicokinetic parameters (parent models)
All modelled toxicokinetic and fit parameters of the parent models are provided in SI B4 (4_TK_parent). The corresponding fits are presented in Fig. S12 to Fig. S21. A heat map providing an overview and clustering of the toxicokinetic rates is presented in Fig. S22. The TK rates of AZ, CY, FLU, and THI at 11°C in *G. pulex* were already used to model pesticide dynamics in Lauper et al. (Lauper et al., 2021).

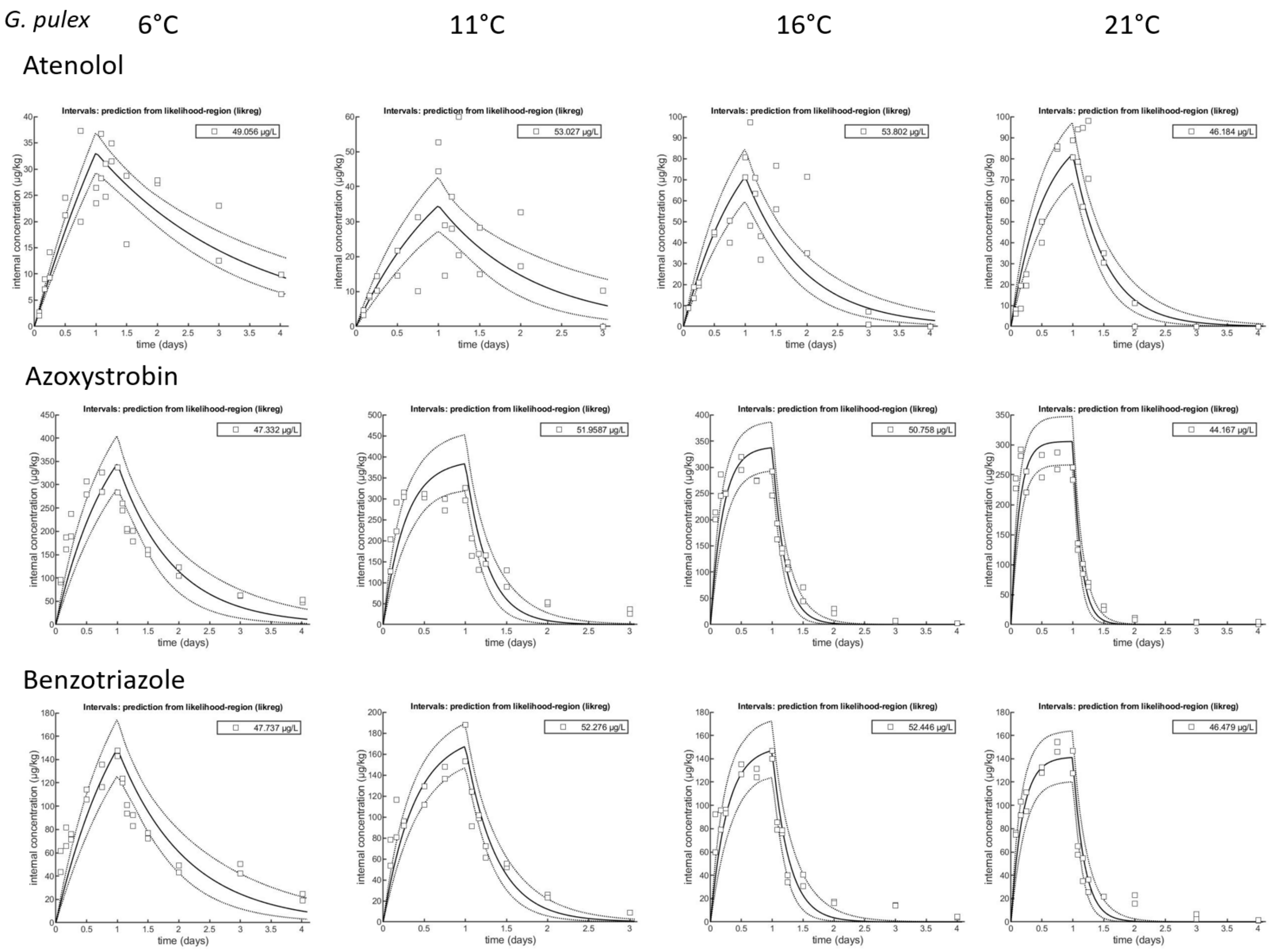

Fig. S12: Toxicokinetic models (parent model) of atenolol, azoxystrobin and benzotriazole in G. pulex. Concentrations presented in µg/kg. Please note different y-axes scales. Legend = C_{Water}.

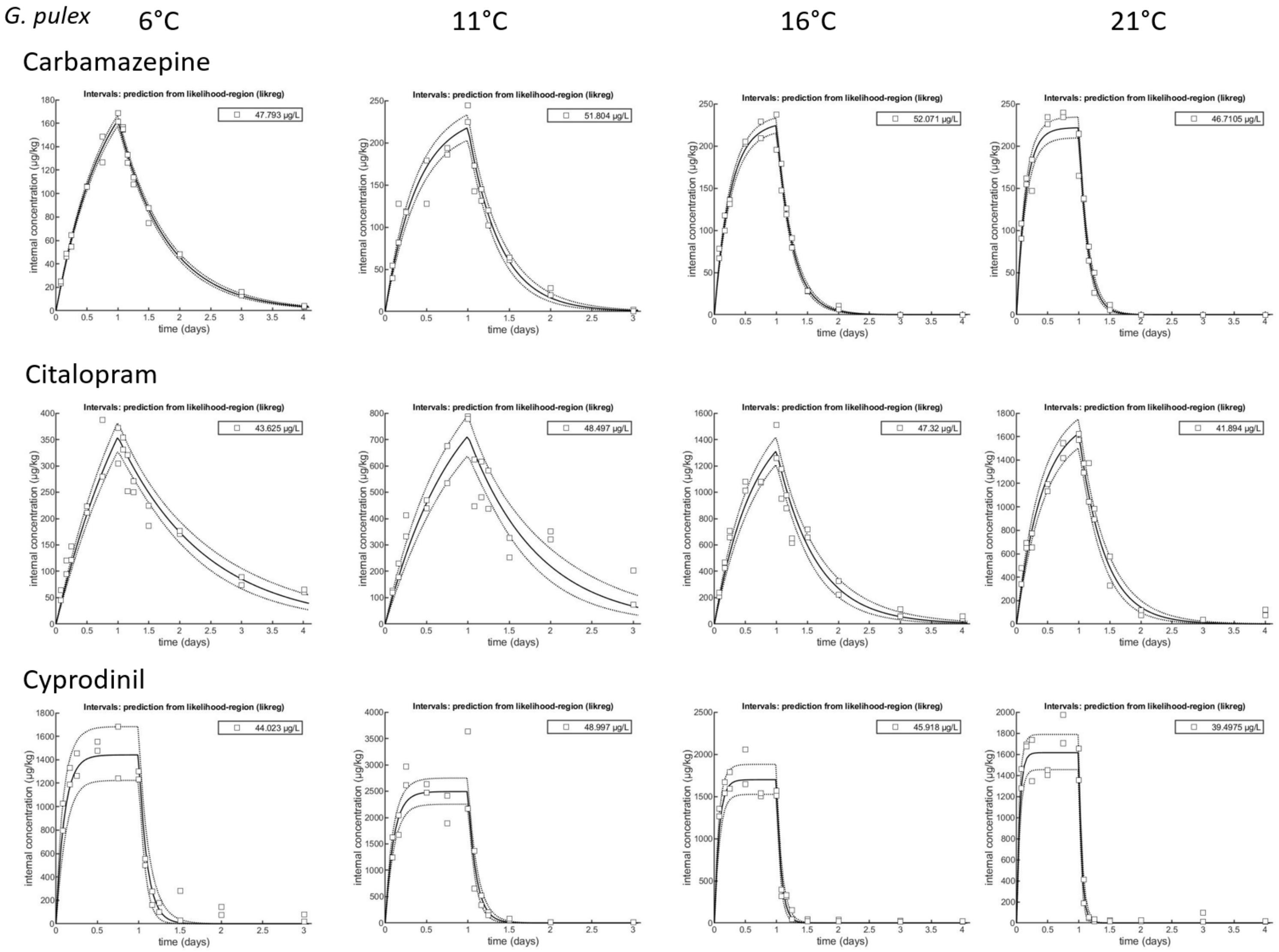

Fig. S13: Toxicokinetic models (parent model) of carbamazepine, citalopram and cyprodinil in G. pulex. Concentrations presented in µg/kg. Please note different y-axes scales. Legend = C_{Water}.

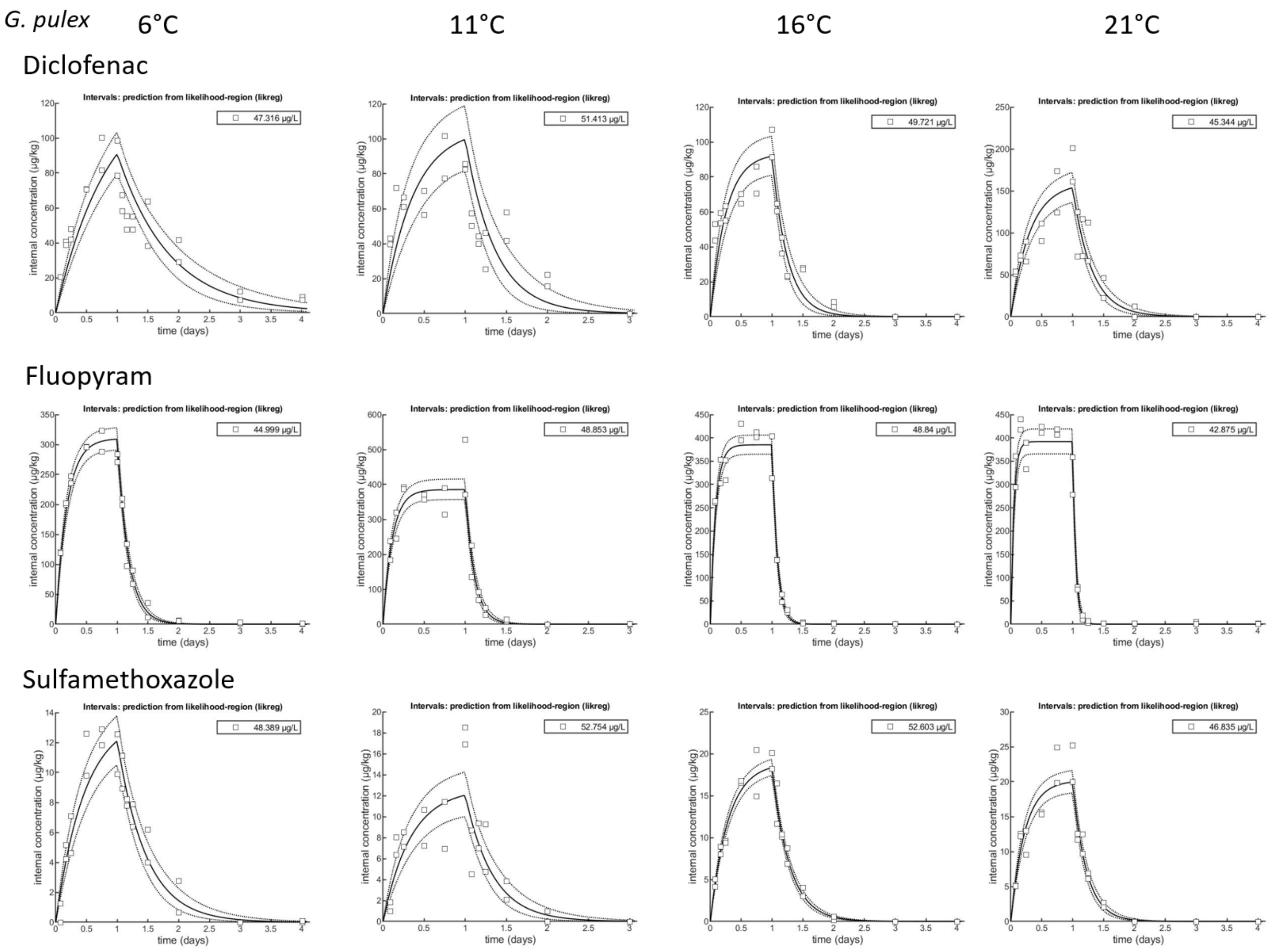

Fig. S14: Toxicokinetic models (parent model) of diclofenac, fluopyram and sulfamethoxazole in G. pulex. Concentrations presented in µg/kg. Please note different y-axes scales. Legend = C_{Water}.

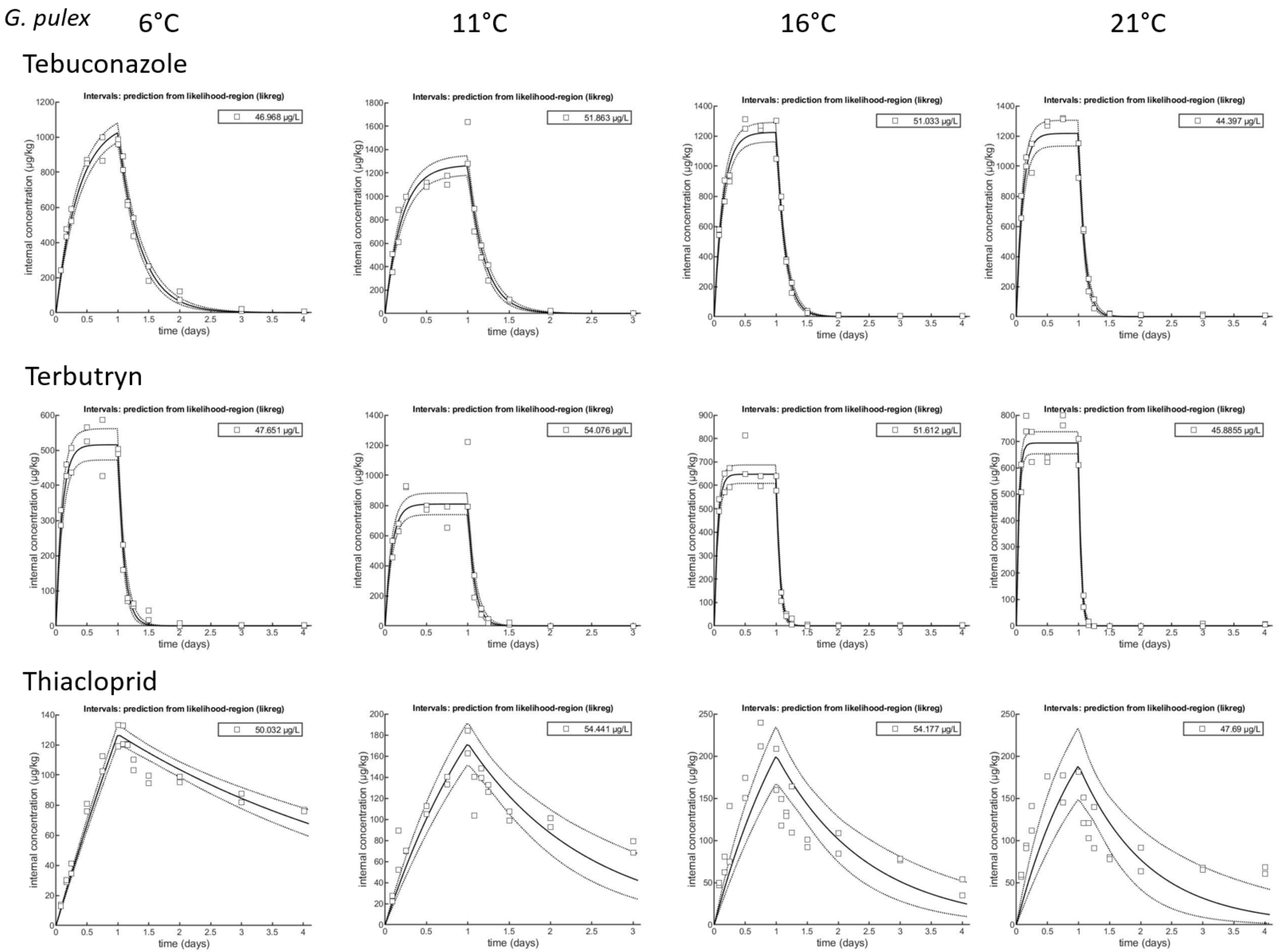

Fig. S15: Toxicokinetic models (parent model) of tebuconazole, terbutryn and thiacloprid in G. pulex. Concentrations presented in µg/kg. Please note different y-axes scales. Legend = C_{Water}.

G. pulex 11°C Alive 11°C Dead 11°C Alive 11°C Dead

Atenolol

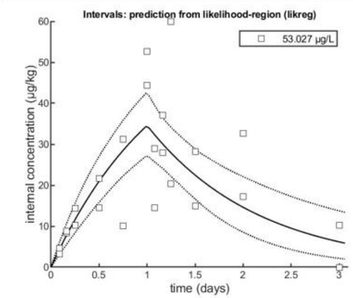

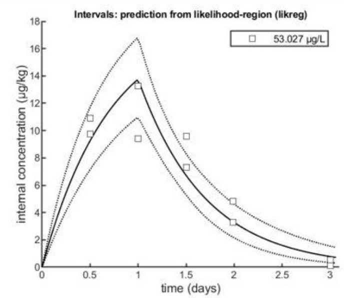

Carbamazepine

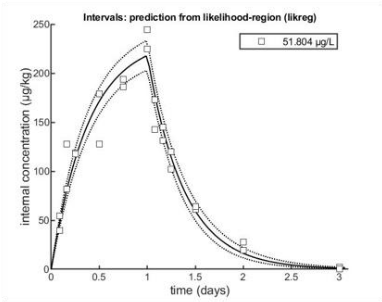

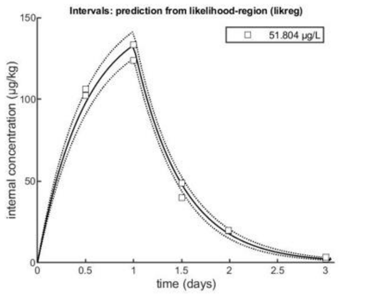

Azoxystrobin

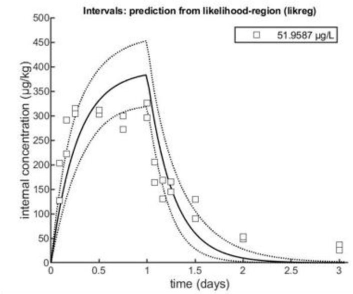

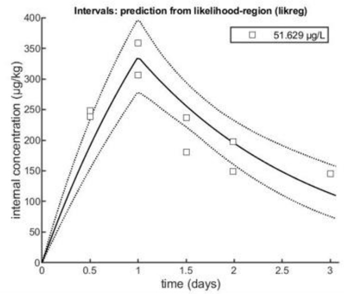

Citalopram

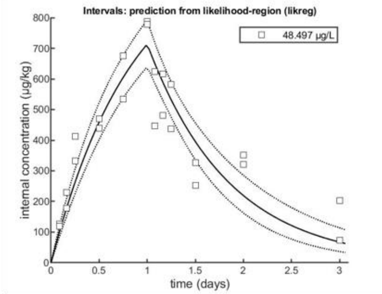

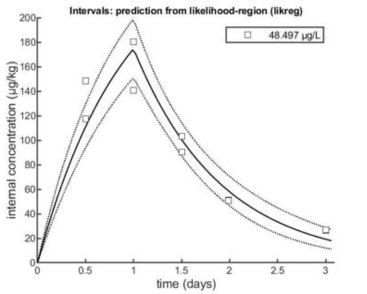

Benzotriazole

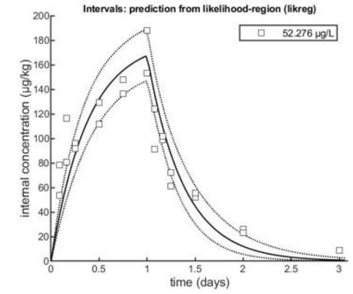

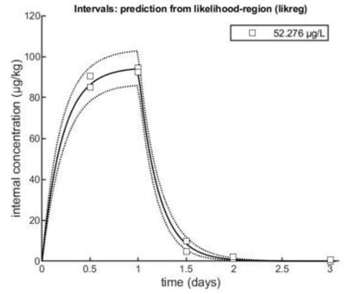

Cyprodinil

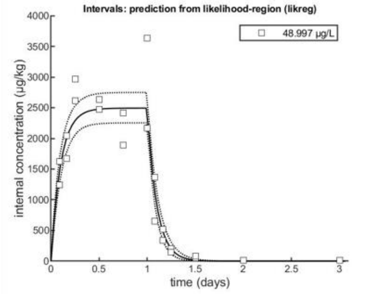

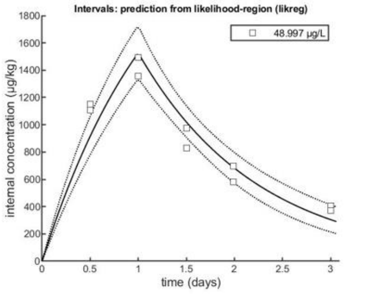

Fig. S16: Toxicokinetic models (parent model) of atenolol, azoxystrobin, benzotriazole, carbamazepine, citalopram and cyprodinil in alive vs. dead G. pulex. Concentrations presented in µg/kg. Please note different y-axes scales. Legend = C_{Water}.

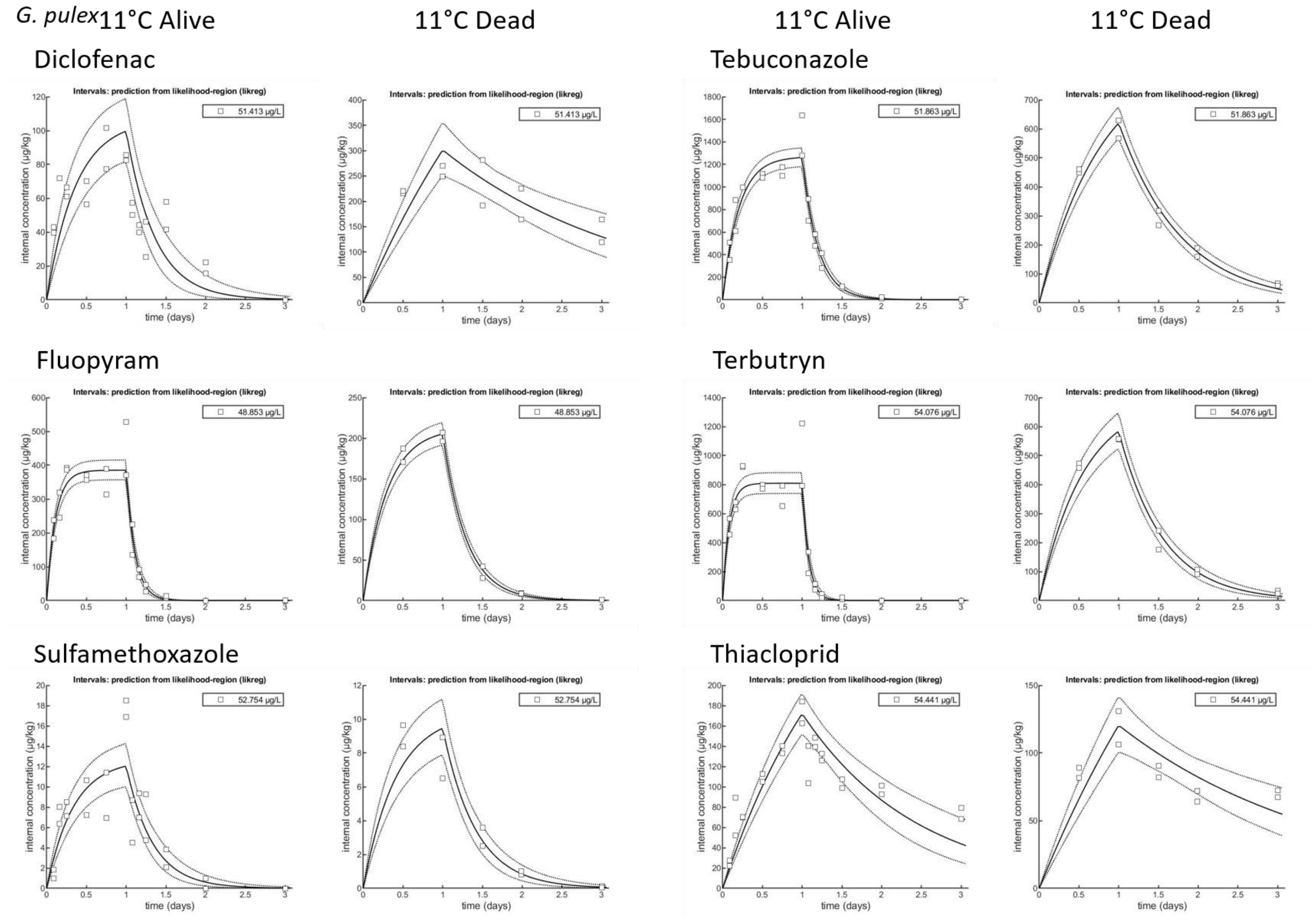

Fig. S17: Toxicokinetic models (parent model) of diclofenac, fluopyram, sulfamethoxazole, tebuconazole, terbutryn and thiacloprid in alive vs. dead G. pulex. Concentrations presented in µg/kg. Please note different y-axes scales. Legend = C_{Water}.

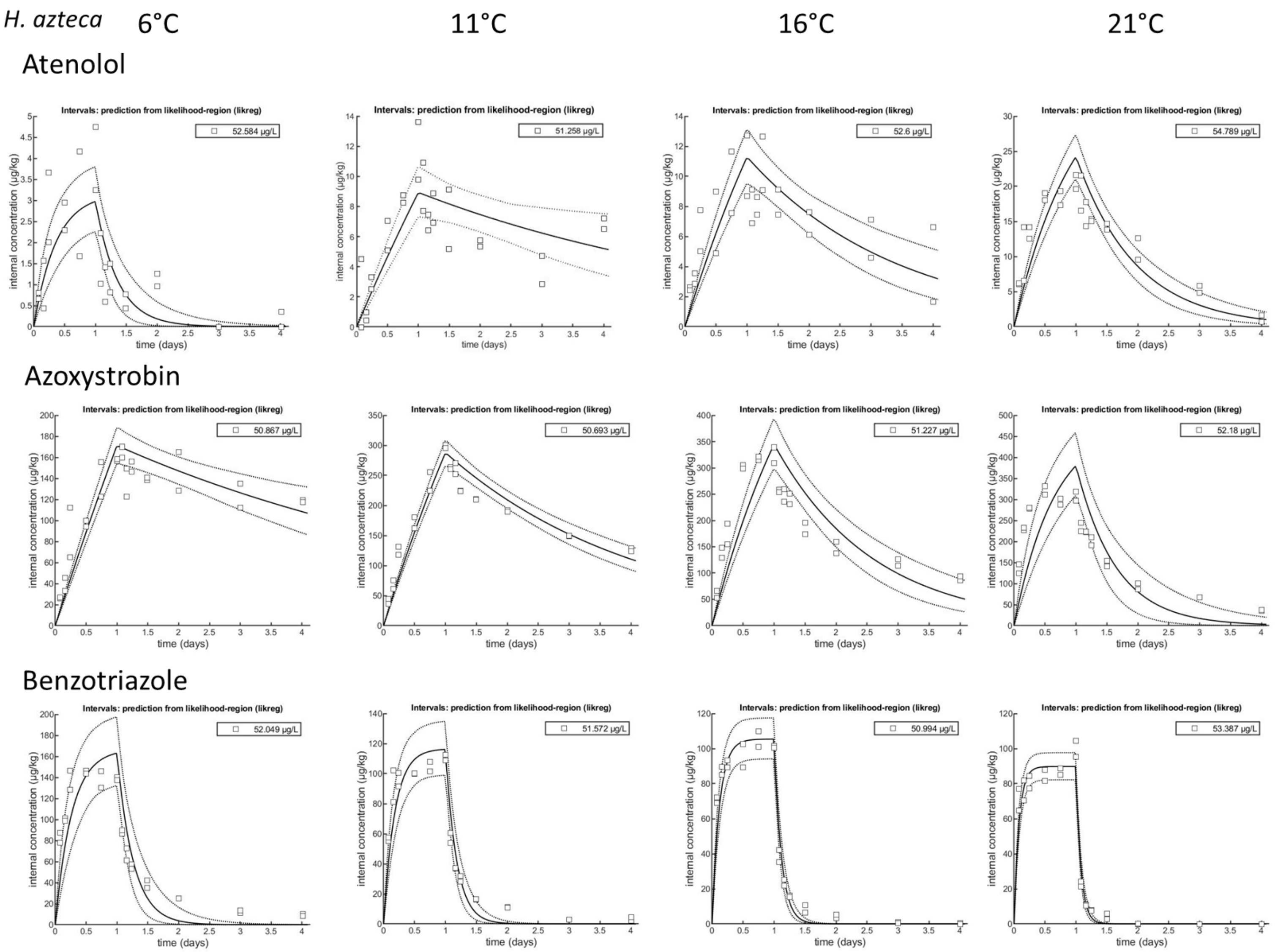

Fig. S18: Toxicokinetic models (parent model) of atenolol, azoxystrobin and benzotriazole in H. azteca. Concentrations presented in µg/kg. Please note different y-axes scales. Legend = C_{Water}.

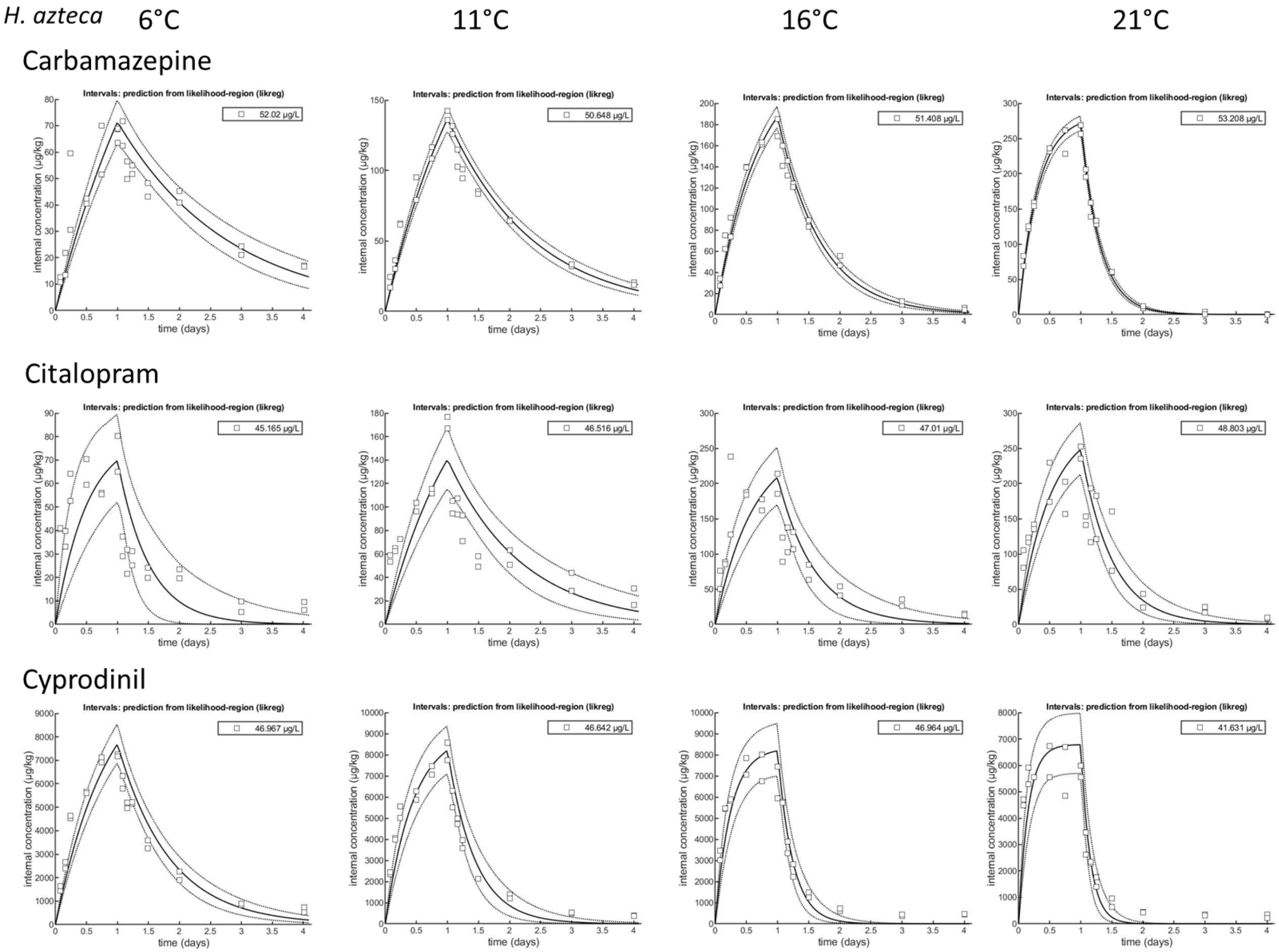

Fig. S19: Toxicokinetic models (parent model) of carbamazepine, citalopram and cyprodinil in H. azteca. Concentrations presented in µg/kg. Please note different y-axes scales. Legend = C_{Water}.

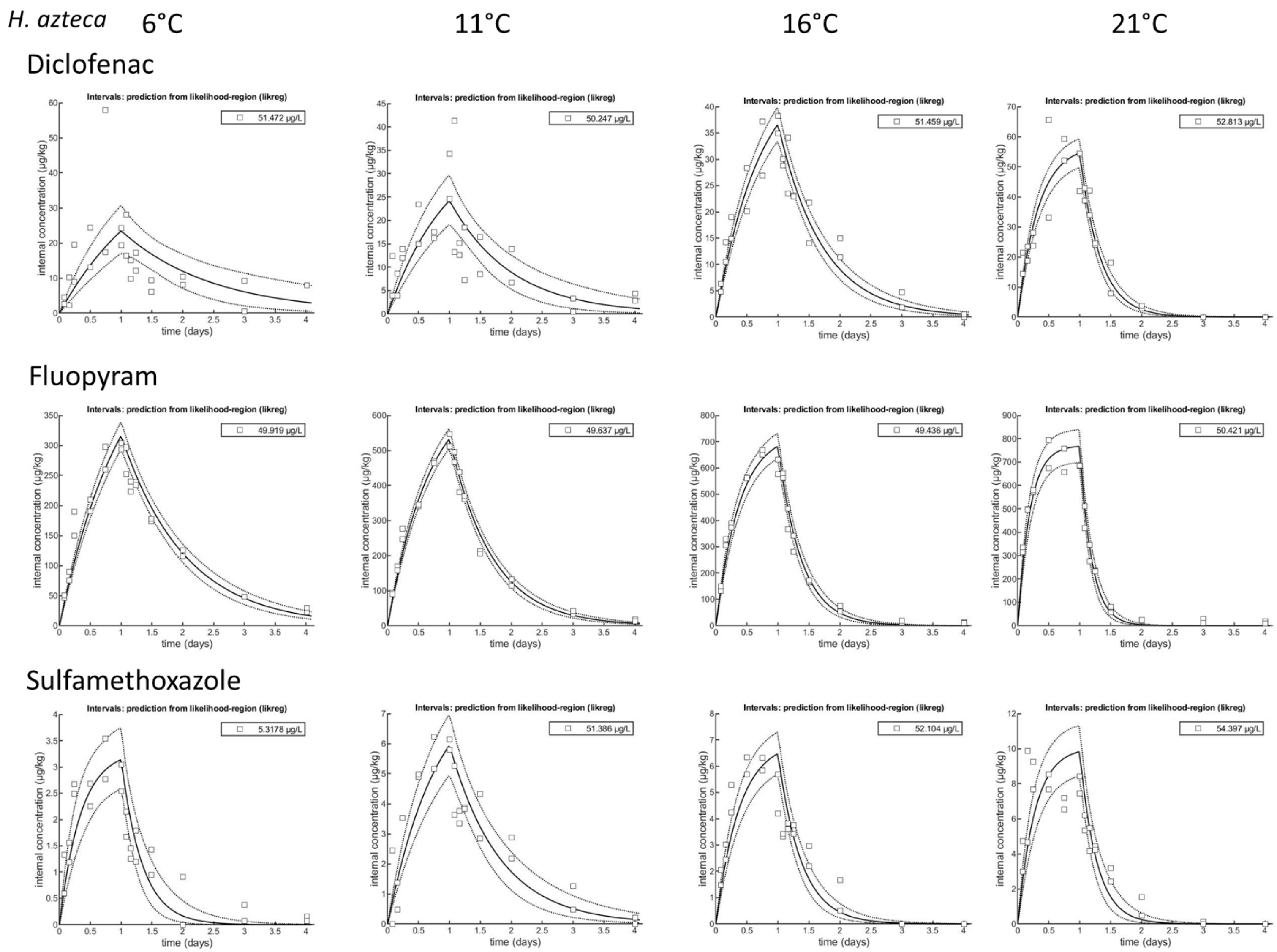

Fig. S20: Toxicokinetic models (parent model) of diclofenac, fluopyram and sulfamethoxazole in H. azteca. Concentrations presented in µg/kg. Please note different y-axes scales. Legend = C_{Water}.

H. azteca 6°C 11°C 16°C 21°C

Tebuconazole

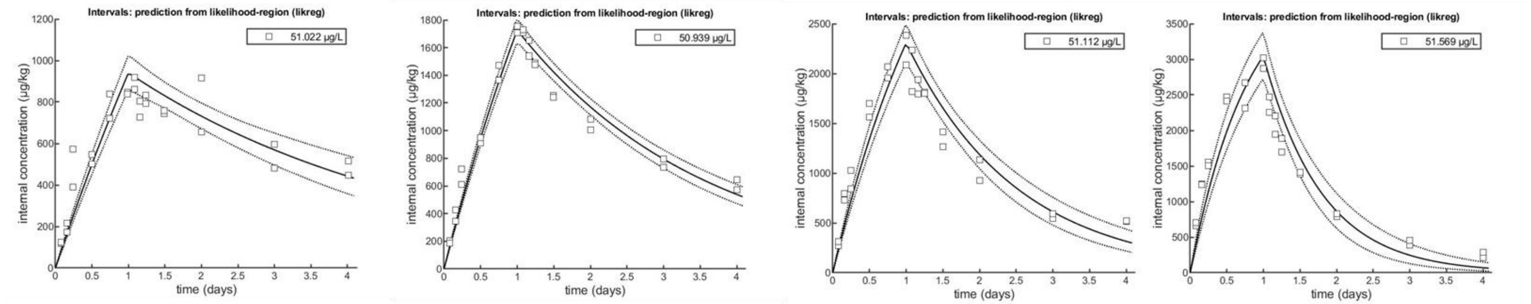

Terbutryn

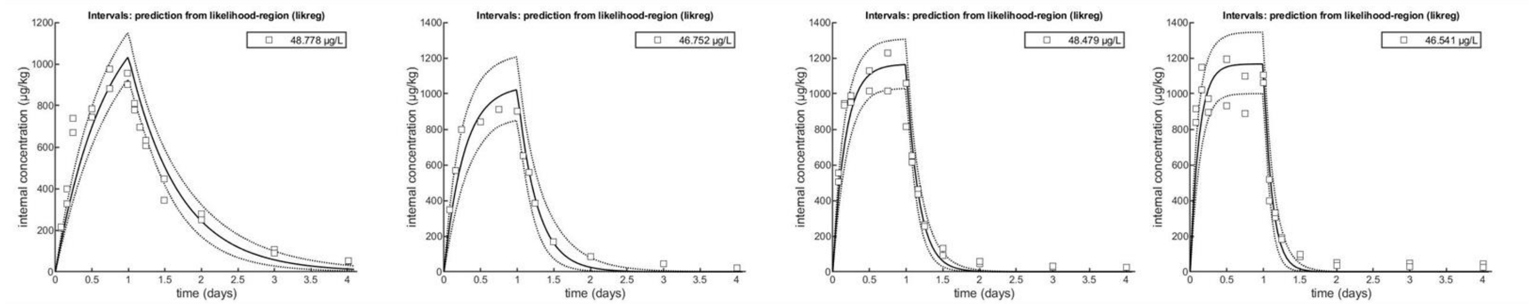

Thiacloprid

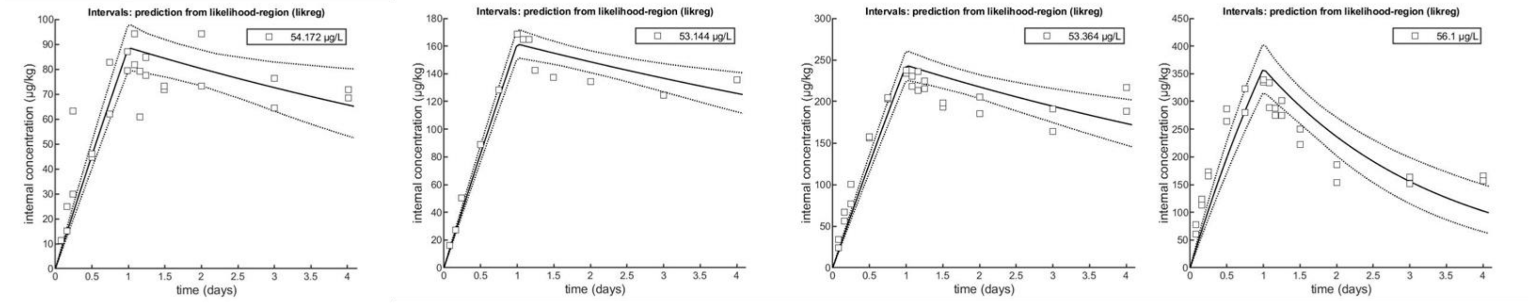

Fig. S21: Toxicokinetic models (parent model) of tebuconazole, terbutryn and thiacloprid in H. azteca. Concentrations presented in µg/kg. Please note different y-axes scales. Legend = C_{Water}.

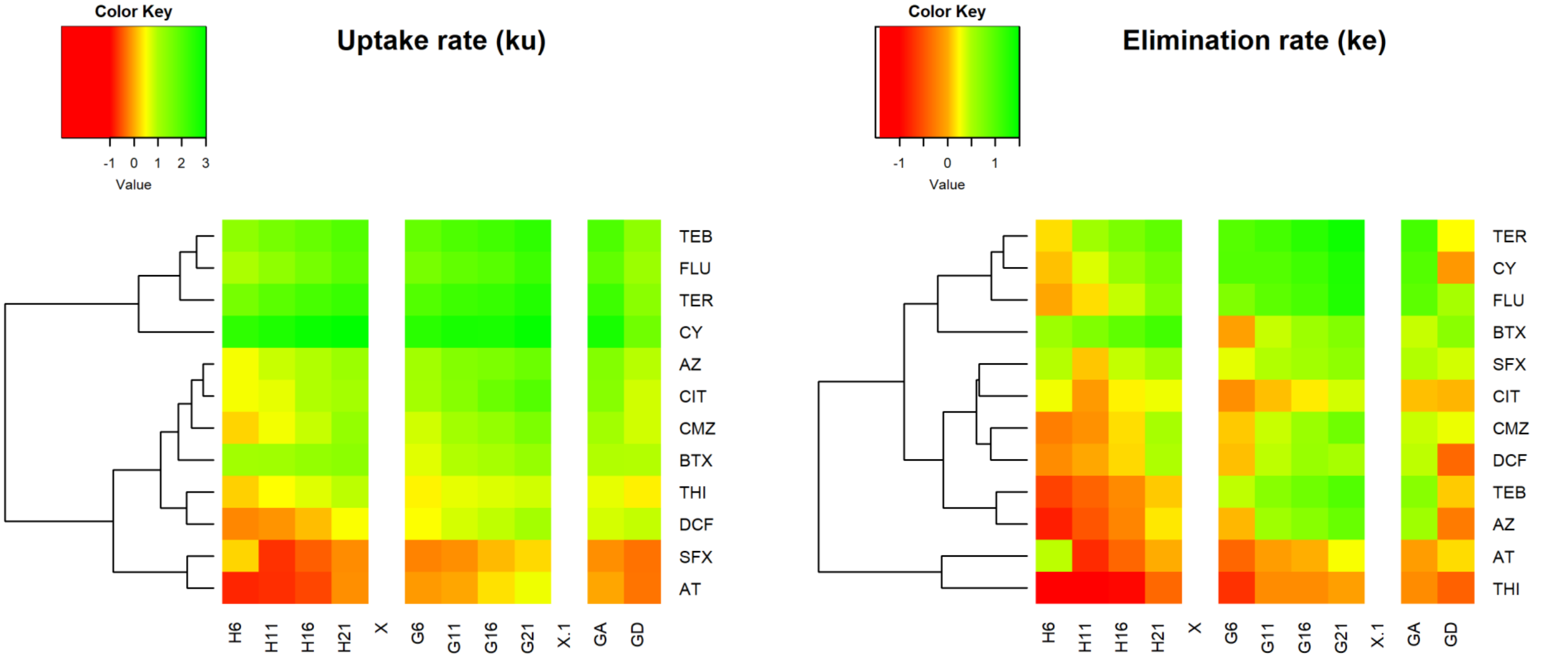

*Fig. S22: Heat maps and clustering of the log-normalised uptake (left) and elimination (right) rates modelled from the toxicokinetic experiments using the first order one-compartment model. Twelve compunds were clustering into three main groups, which were mostly similar between uptake and elimination rate. G = G. pulex, H = H. azteca. GA = alive gammarids, GD = dead gammarids, X and X.1 = column seperators. AT = atenolol, AZ = azoxystrobin, BTX = benzotriazole, CIT = citalopram, CMZ = carbamazepine, CY = cyprodinil, DCF = diclofenac, FLU = fluopyram, SFX = sulfamethoxazole, TEB = tebuconazole, TER = terbutryn, THI = thiacloprid. * = AZ and THI should be evaluated carefully as they showed obvious two-compartment kinetics.*

SI A11: Confirmation of the modelled toxicokinetic parameters using the pre-test dataset

The measured (pre-test, SI A9) and based on the obtained toxicokinetic parameters (k_u and k_e of the main experiment, SI A10) predicted internal concentrations showed a good agreement for all compounds except for citalopram (Fig. S23 to Fig. S25). This demonstrated the inter-experimental transferability of the obtained toxicokinetic parameters as well as underlines the independence of internal concentrations and lipid content (which was two times higher in the pre-test) of the tested polar compounds. The citalopram concentrations were up to about three times lower than predicted or measured in the main experiment. This could be either explained by different physiological properties of the two tested gammarid batches (May and September population) that might have been of importance for the proposed active uptake pathway of citalopram (i.e. cation channels). Another explanation could be the presence of two further cations in the exposure mix of the pre-test, which might have competed with the active uptake of citalopram. Predicted internal concentrations for the other cation, atenolol, were also generally higher but the deviation less stringent.

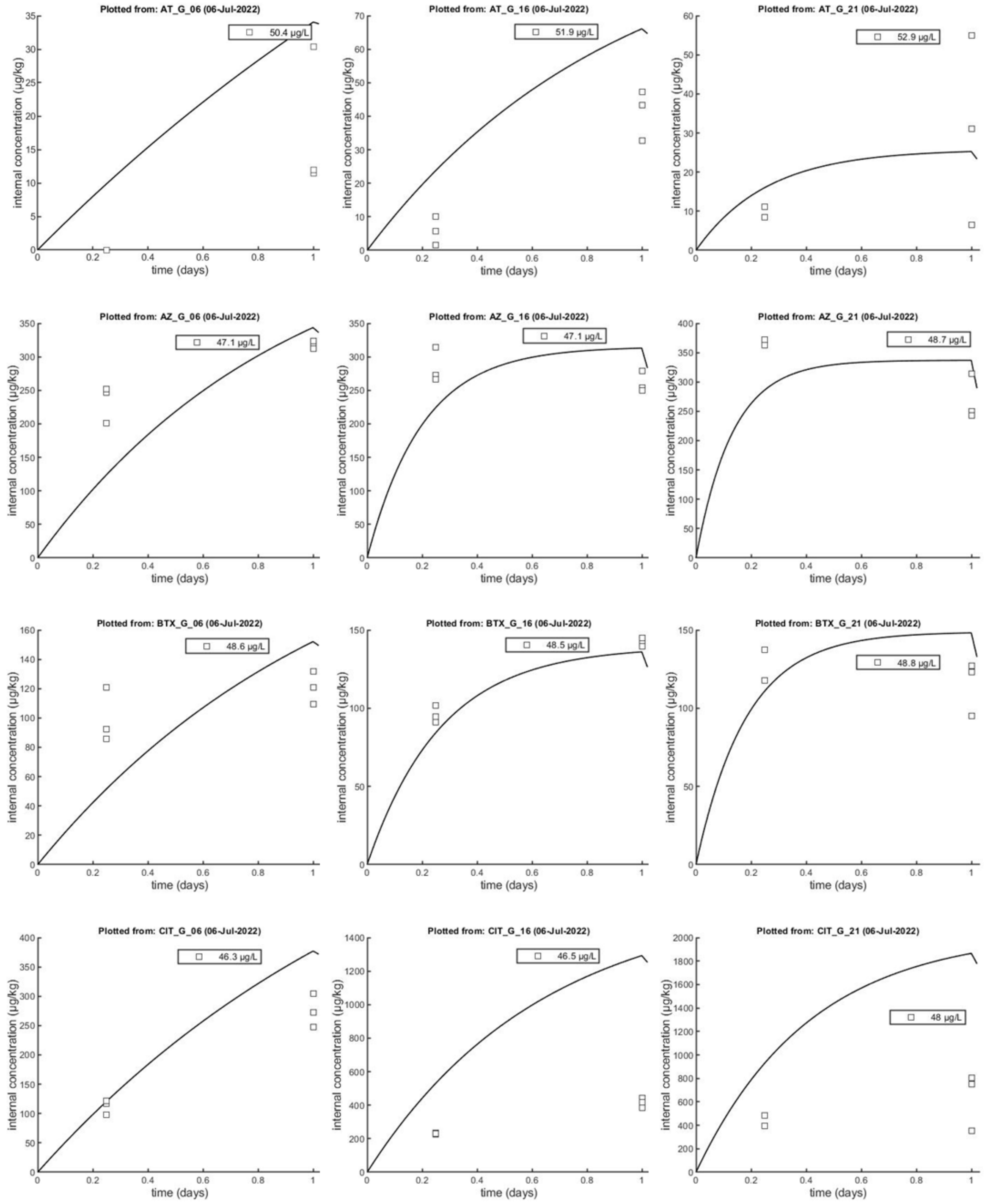

Fig. S23: Modelled (lines) and individual measured (square) concentrations (6 h and 24 h) of the pre-test, part 1/3. Model fits are based on the modelled parameters presented in section SI A10 and the measured medium concentrations of the pre-test (SI A9). AT = atenolol, AZ = azoxystrobin, BTX = benzotriazole, CIT = citalopram, G = G. pulex. The following number represents the test temperature (6°C left, 16°C centre, 21°C right). Legend = C_{Water}.

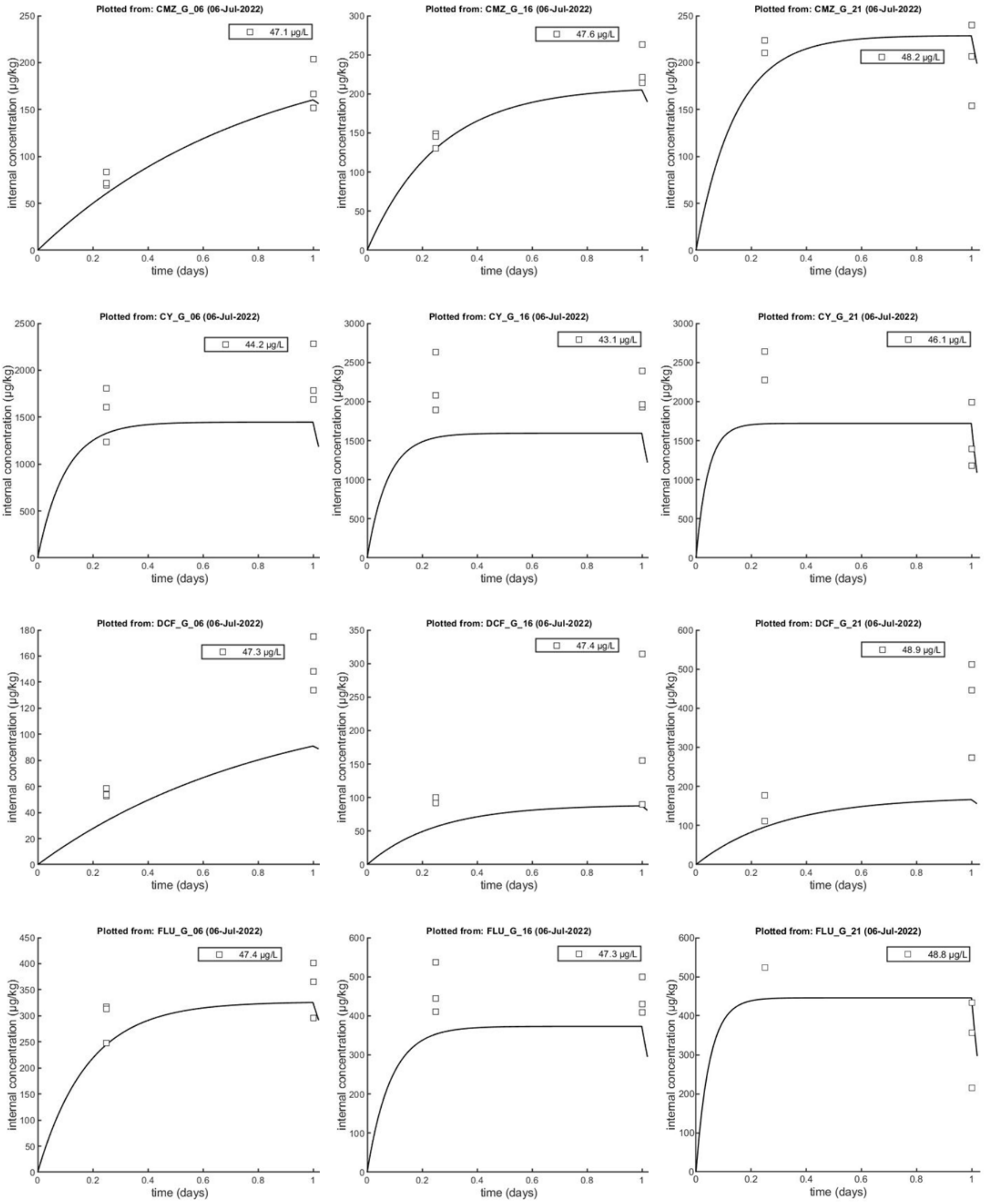

Fig. S24: Modelled (lines) and individual measured (square) concentrations (6 h and 24 h) of the pre-test, part 2/3. Model fits are based on the modelled parameters presented in section SI A10 and the measured medium concentrations of the pre-test (SI A9). CMZ = carbamazepine, CY = cyprodinil, DCF = diclofenac, FLU = fluopyram, G = G. pulex. The following number represents the test temperature (6°C left, 16°C centre, 21°C right). Legend = C_{Water}.

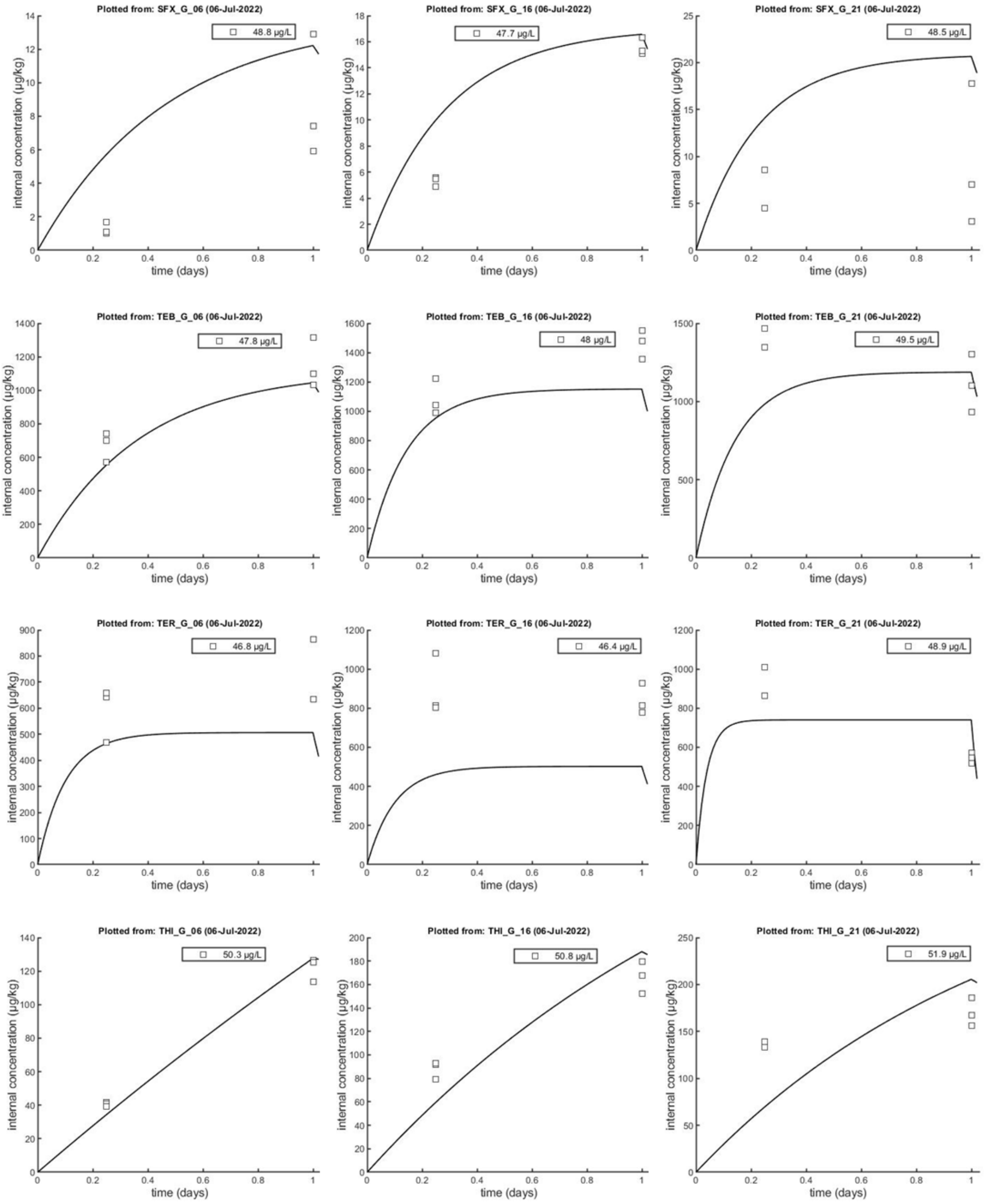

Fig. S25: Modelled (lines) and individual measured (square) concentrations (6 h and 24 h) of the pre-test, part 3/3. Model fits are based on the modelled parameters presented in section SI A10 and the measured medium concentrations of the pre-test (SI A9). SFX = sulfamethoxazole, TEB = tebuconazole, TER = terbutryn, THI = thiacloprid, G = G. pulex. The following number represents the test temperature (6°C left, 16°C centre, 21°C right). Legend = C_{Water}.

SI A12: Modelled toxicokinetic parameters (biotransformation models)

BTP classification

The classifications applied for the biotransformation models are presented in Tab. S11. Additionally, the biotransformation pathways of citalopram are presented in Fig. S26.

The observed differences in biotransformation were mostly quantitative, but little qualitative differences, i.e. kind of BTPs, were observed. The exposure concentrations were lower compared to experiments designed for BTP screening (Fu et al., 2018, 2020; Jeon et al., 2013; Rösch et al., 2016) so that some BTP concentrations might fall below LOD. However, the total biomass in *H. azteca* samples was only two times lower than in gammarids, which makes it unlikely that many BTPs were missed due to higher LODs in *H. azteca*. It is also important to mention, that no non-target analysis of BTPs was performed. BTP analysis was based on a suspect screening from literature reports. For azoxystrobin, diclofenac, tebuconazole and terbutryn it is assumed that previously reported BTPs are comprehensive (Fu et al., 2018, 2020; Jeon et al., 2013; Rösch et al., 2016).

Tab. S11: Biotransformation product classification for the biotransformation models.

Parent	BTP	Classification	Pathway	Reference
**Azoxystrobin**	AZ_M362a	1st	-C2H2O	(Fu et al., 2018)
**Azoxystrobin**	AZ_M362b	1st	-C2H2O	(Fu et al., 2018)
**Azoxystrobin**	AZ_M378	2nd	Ester hydrolysis → hydrogenation → demethylation	(Fu et al., 2018)
**Azoxystrobin**	AZ_M392	2nd	Ester hydrolysis → hydrogenation	(Fu et al., 2018)
**Azoxystrobin**	AZ_M638 (H)	2nd	Demethylation → glucose conjugation → malonyl conjugation	(Fu et al., 2018)
**Azoxystrobin**	AZ_M640 (H)	2nd	Demethylation → glucose conjugation → malonyl conjugation → hydrogenation	(Fu et al., 2018)
**Azoxystrobin**	Azoxystrobin acid (AZ_M390b)	1st	Ester hydrolysis	(Fu et al., 2018)
**Citalopram**	CIT N-desmethyl	1st	N-dealkylation	(Sangkuhl et al., 2011), Fig. S26
**Citalopram**	CIT N-oxide	1st	N-oxidation	(Sangkuhl et al., 2011), Fig. S26
**Citalopram**	CIT didesmethyl	2nd	N-dealkylation	(Sangkuhl et al., 2011), Fig. S26
**Cyprodinil**	CY_CGA_304075 (CY_M242a)	1st	Hydroxylation at benzene group	Fig. S3
**Cyprodinil**	CY_M242b	1st	Hydroxylation at benzene group	Fig. S3
**Diclofenac**	DCF methylesther	1st	Carboxylic acid methylation	(Fu et al., 2020)
**Tebuconazole**	TEB_M324a	1st	Hydroxylation at tert-butyl group	(Rösch et al., 2016)
**Terbutryn**	Irgarol-descyclopropyl (TER_M214)	1st	Dealkylation	(Jeon et al., 2013)
**Terbutryn**	TER_M258a	1st	Hydroxylation at tert-butyl group	(Jeon et al., 2013)
**Terbutryn**	TER_M272	2nd	Hydroxylation → oxidation	(Jeon et al., 2013)
**Terbutryn**	TER_M315a	2nd	Glutathione conjugation → carboxyl peptidase → glutamyl transpeptidase → rearangement	(Jeon et al., 2013)
**Terbutryn**	TER_M315b	2nd	Glutathione conjugation → carboxyl peptidase → glutamyl transpeptidase → rearangement	(Jeon et al., 2013)

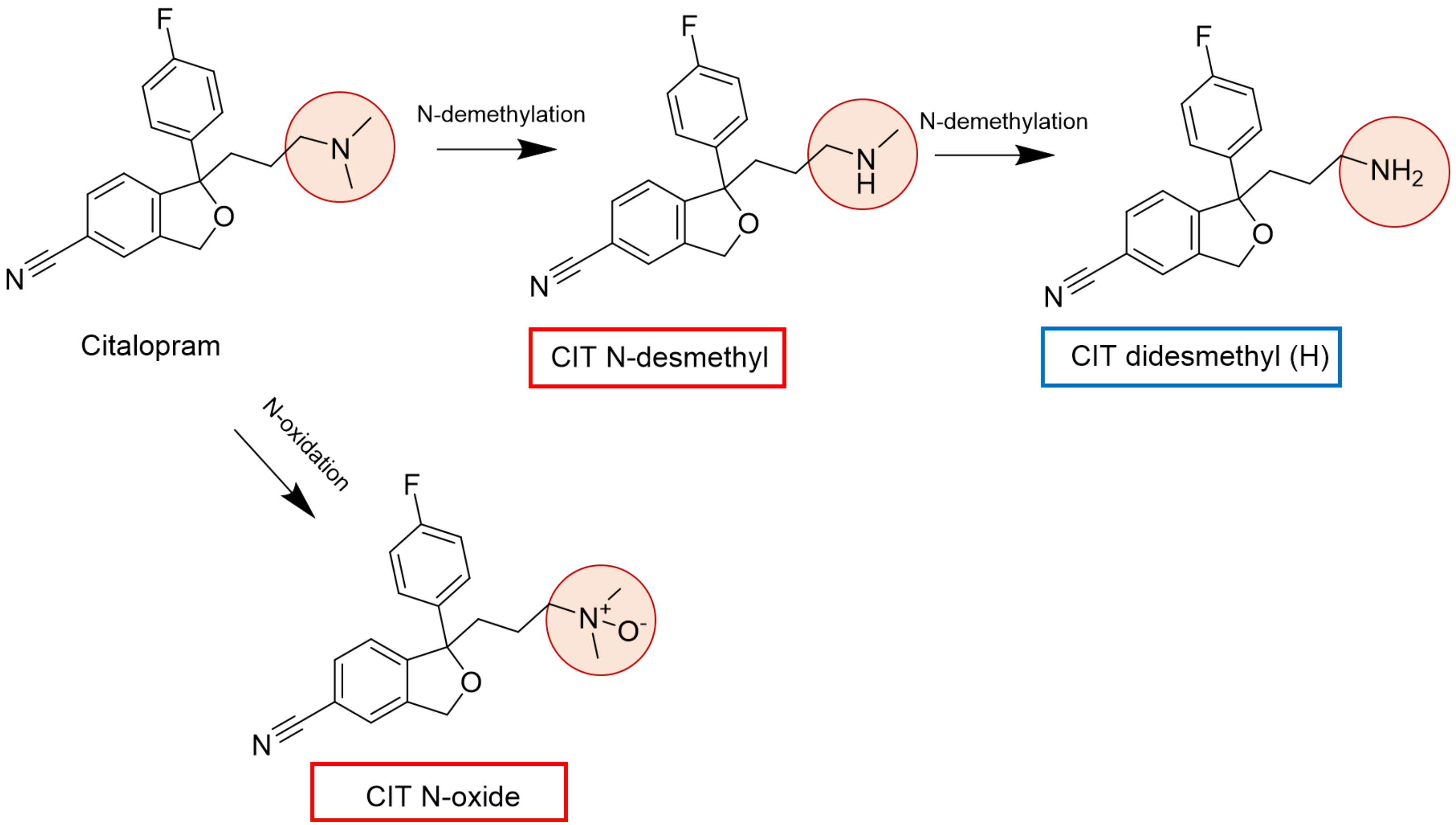

Fig. S26: Biotransformation pathways of citalopram in G. pulex and H. azteca according to Sangkuhl et al. (2011). (H) = only in H. azteca. Red = primary BTP, blue = secondary BTP. CIT = citalopram. CIT N-oxide was only a minor BTP.

Modelled parameters

All modelled toxicokinetic and fit parameters of the biotransformation models are presented in SI B5 (5_TK_BTP). The corresponding fits are presented in Fig. S28 to Fig. S31. An overview of the toxicokinetic rates is presented as a heat map in Fig. S27.

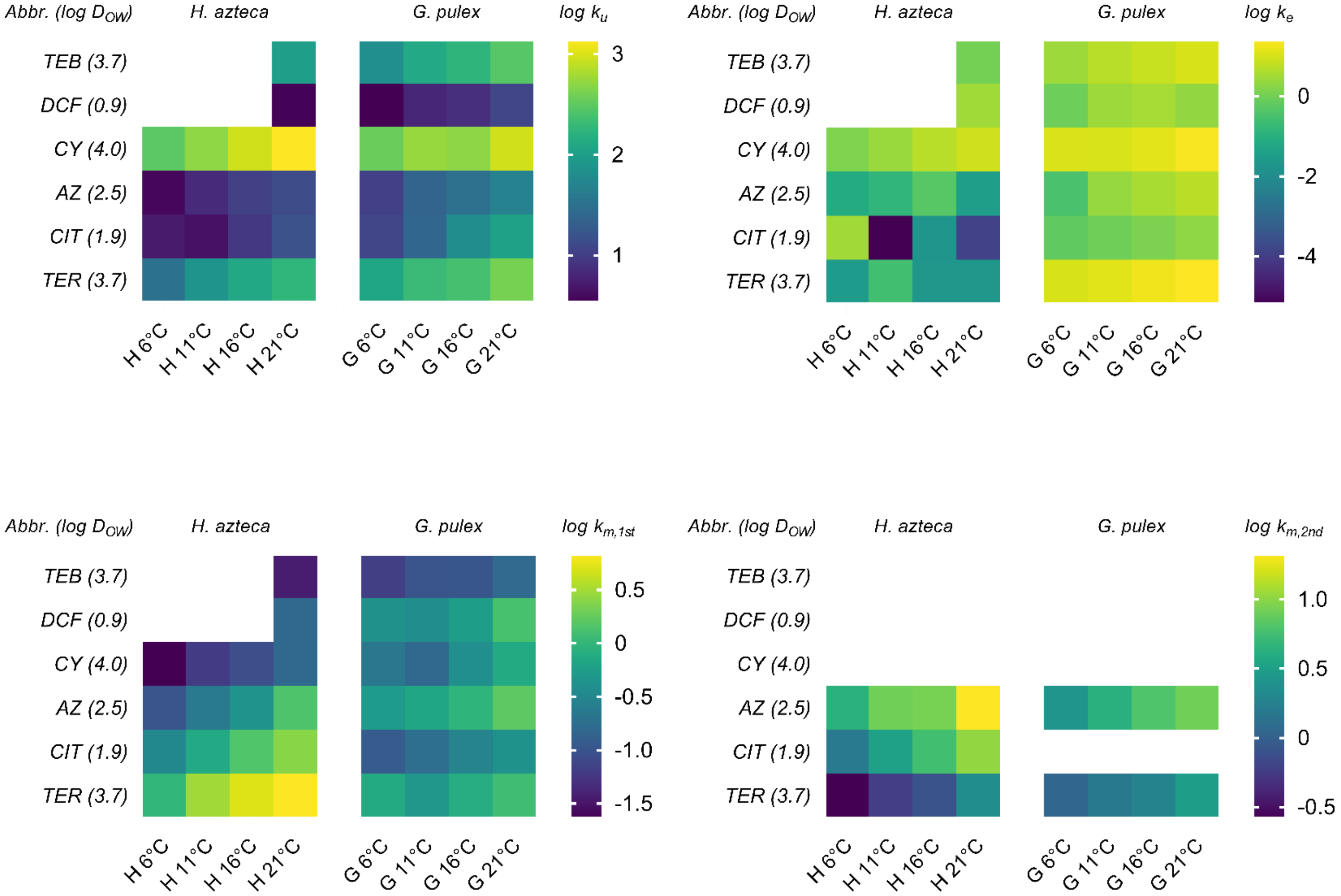

*Fig. S27: Heat map of the modelled toxicokinetic rates (biotransformation model). Uptake rate (top left), elimination rate (top right), primary biotransformation rate (bottom left) and secondary biotransformation rate (bottom right). The log D_{ow} is shown in brackets behind the compound shortcut. Compounds are sorted by increasing primary biotransformation rate at 21°C in H. azteca. AZ = azoxystrobin, CIT = citalopram, CY = cyprodinil, DCF = diclofenac, TEB = tebuconazole, TER = terbutryn. * = AZ should be evaluated carefully as it showed obvious two-compartment kinetics.*

G. pulex 6°C 11°C 16°C 21°C

Azoxystrobin

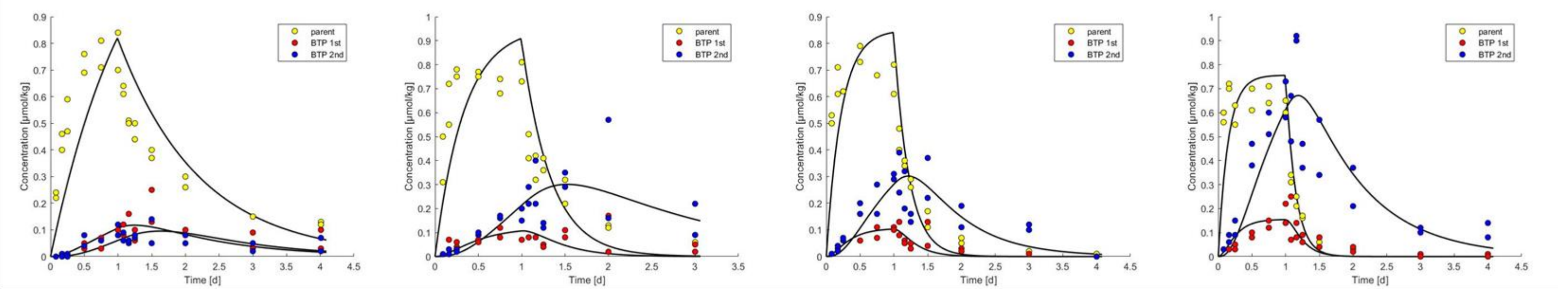

Citalopram

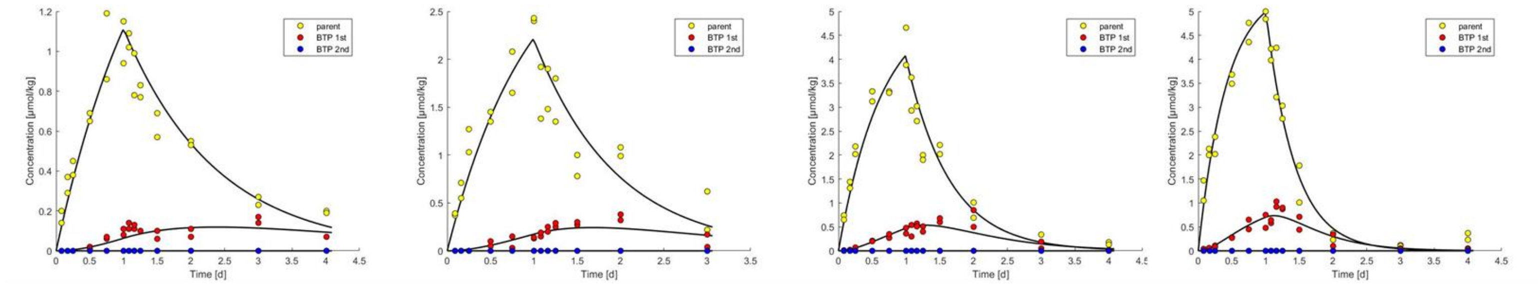

Cyprodinil

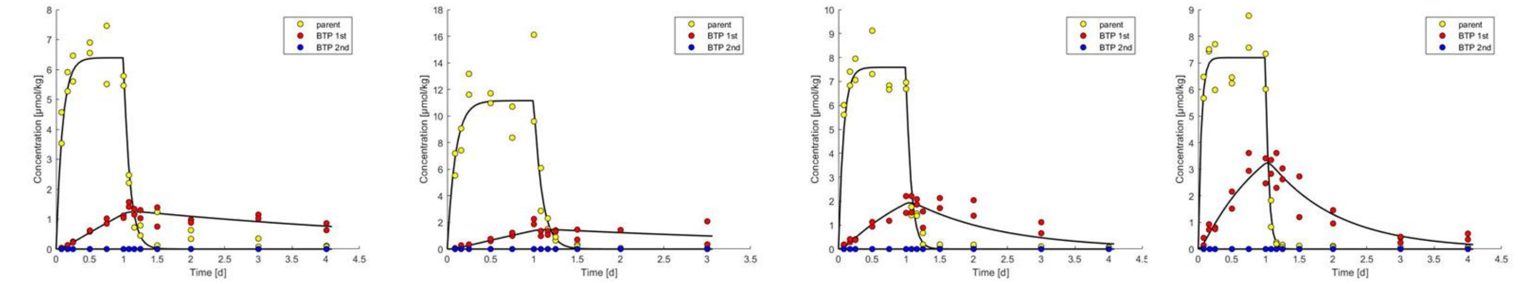

Fig. S28: Toxicokinetic models (biotransformation model) of azoxystrobin, citalopram and cyprodinil in G. pulex. Concentrations presented in µmol/kg. Please note different y-axes scales.

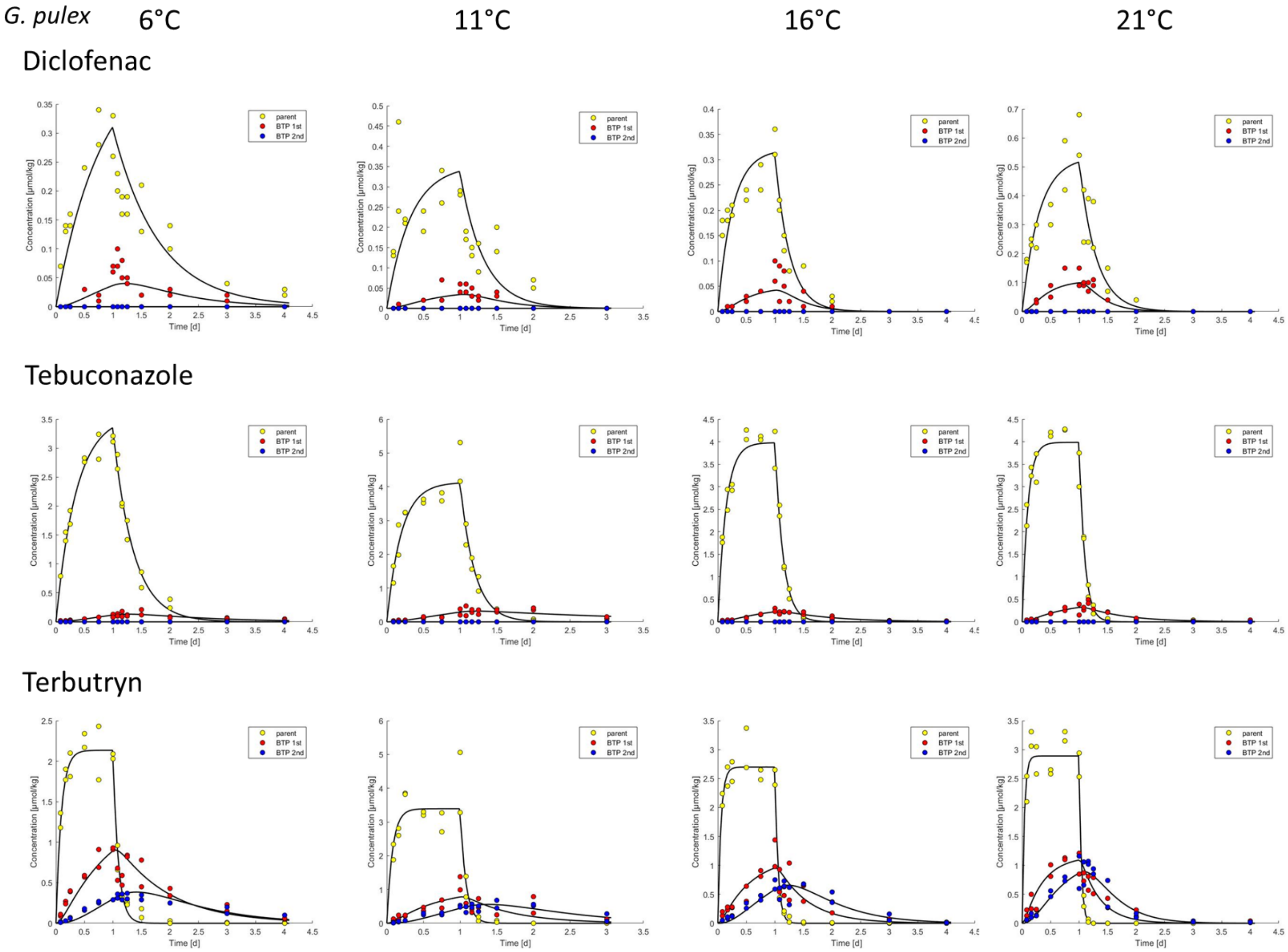

Fig. S29: Toxicokinetic models (biotransformation model) of diclofenac, tebuconazole and terbutryn in G. pulex. Concentrations presented in µmol/kg. Please note different y-axes scales.

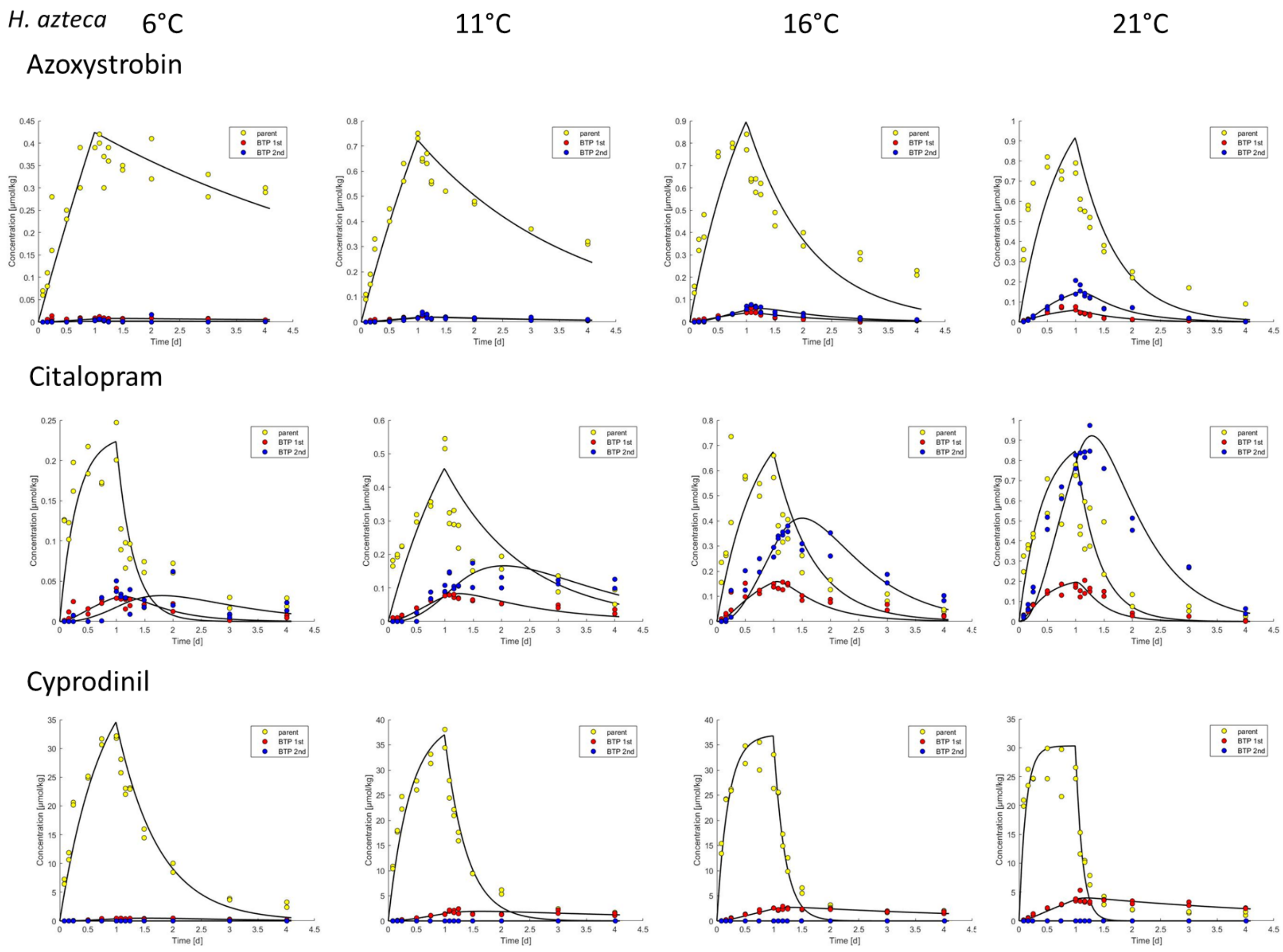

Fig. S30: Toxicokinetic models (biotransformation model) of azoxystrobin, citalopram and cyprodinil in H. azteca. Concentrations presented in µmol/kg. Please note different y-axes scales.

H. azteca 6°C 11°C 16°C 21°C

Diclofenac

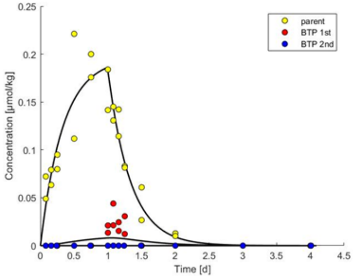

Tebuconazole

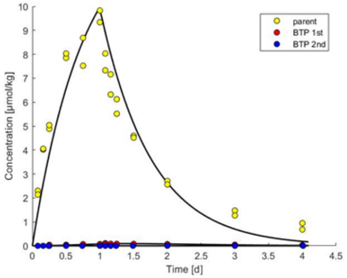

Terbutryn

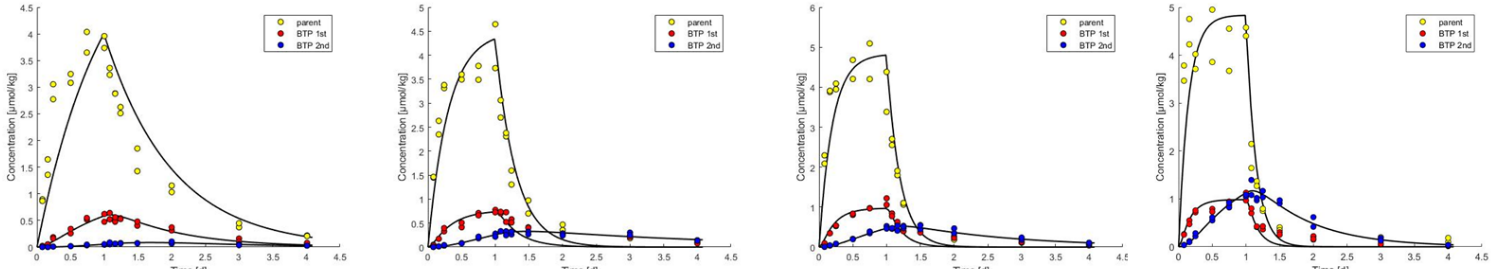

Fig. S31: Toxicokinetic models (biotransformation model) of diclofenac, tebuconazole and terbutryn in H. azteca. Concentrations presented in µmol/kg. Please note different y-axes scales. BTP concentrations of diclofenac and tebuconazole at temperatures lower than 21°C were too low to be quantified, thus no models were fitted for these treatments.

A comparison of the modelled elimination rates and primary biotransformation rates is presented in Fig. S32. Biotransformation contributed only a minor proportion to the parent elimination in *G. pulex* whereas biotransformation rates in *H. azteca* were partially so high that the parent elimination rate could not be distinguished from zero.

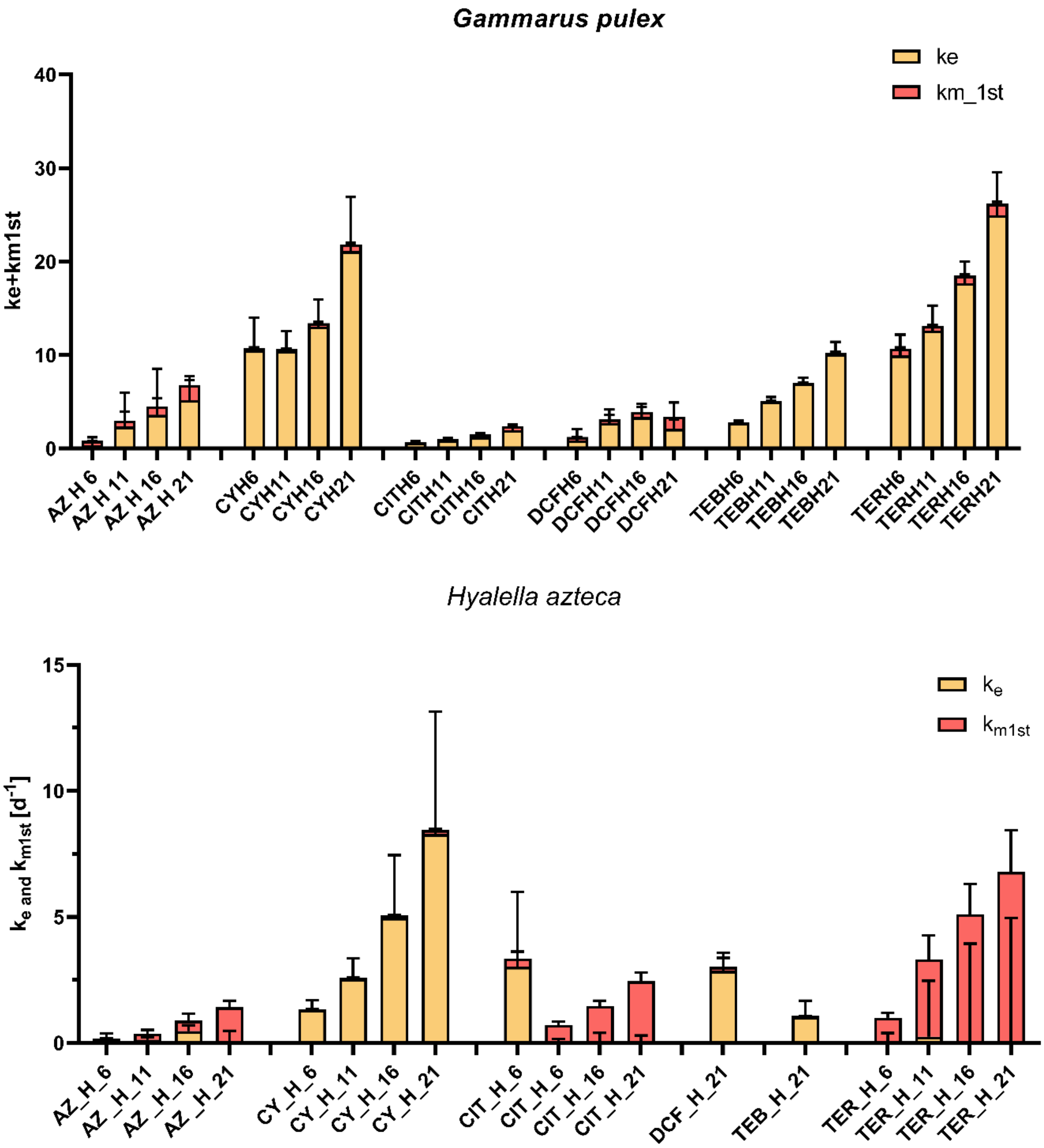

*Fig. S32: A comparison of the modelled elimination and primary biotransformation rates. Presented as modelled rate ± 95% CI. AZ = azoxystrobin, CIT = citalopram, CY = cyprodinil, DCF = diclofenac, TEB = tebuconazole, TER = terbutryn. * = AZ should be evaluated carefully as it showed obvious two-compartment kinetics. Please note the different y-axis scales. G = G. pulex, H = H. azteca. The numbers indicate the test temperatures in °C.*

SI A13: Comparison of modelled toxicokinetic parameters with literature values

A comparison of the modelled toxicokinetic parameters with previous studies is presented in Tab. S12 to Tab. S15. Most of the referred parameters originate from studies that used organisms of the same origin populations than the present study (Arlos et al., 2020; Fu et al., 2018; Kosfeld et al., 2020). Other populations were used by for carbamazepine and diclofenac (Meredith-Williams et al., 2012; Miller et al., 2016, 2017). The compared toxicokinetic parameters show mostly a very good agreement (same order of magnitude and overlapping confidence intervals) between the different studies, except for Miller et al., (2016, 2017).

*Tab. S12: Comparison of the modelled toxicokinetic parameters (parent model, G. pulex) of the present study with literature values. * = rates would show much better agreement with reference studies under the assumption they were calculated on a h^{-1} basis (as the graphs in the references imply) but mistakenly reported on a d^{-1} basis, which would result in 24 times higher values.*

**Parent model *G. pulex***			
**Compound**	**Rates**	**BCF_{kin}**	**Experimental conditions**
**Atenolol**	Arlos et al. 2020:<br>ku: 0.42 [0.32, 0.53]<br>ke: 0.12 [0.02, 0.24]	3.4	50 µg/L exposure concentration<br>48 h uptake and 48 h depuration phase<br>12h/12h light/dark cycle<br>Controlled temperature 11°C, pH 7.9
	Present study:<br>ku: 1.0 [0.7, 1.3]<br>ke: 0.9 [0.4, 1.4]	1.1	50 µg/L exposure concentration<br>24 h uptake and 48 h depuration phase<br>16h/8h light/dark cycle<br>Controlled temperature 11°C, pH 7.9
**Benzotriazole**	Arlos et al. 2020:<br>ku: 8.72 [6.00, 12.84]<br>ke: 4.93 [3.62, 7.21]	1.8	50 µg/L exposure concentration<br>48 h uptake and 48 h depuration phase<br>12h/12h light/dark cycle<br>Controlled temperature 11°C, pH 7.9
	Present study:<br>ku: 9.1 [7.1, 11.6]<br>ke: 2.6 [2.0, 3.4]	3.5	50 µg/L exposure concentration<br>24 h uptake and 48 h depuration phase<br>16h/8h light/dark cycle<br>Controlled temperature 11°C, pH 7.9
**Carbamazepine**	Meredith-Williams et al 2012:<br>ku: 5.199 ± 0.6551<br>ke: 0.7207 ± 0.1298	7.2	100 µg/L exposure concentration<br>48 h uptake and 72 h depuration phase<br>12h/12h light/dark cycle<br>Controlled temperature 12°C ± 2°C, pH range of 8.48–9.15
	Miller et al, 2017*:<br>ku: 0.5307 ± 0.115<br>ke: 0.0214 ± 0.058	24.8	10 µg/L exposure concentration<br>48 h uptake and 48 h depuration phase<br>12h/12h light/dark cycle<br>Controlled temp of 15°C average pH of 8.19 ± 0.05
	Arlos et al. 2020:<br>ku: 6.05 [5.11, 6.93]<br>ke: 1.68 [1.46, 1.93]	3.6	50 µg/L exposure concentration<br>48 h uptake and 48 h depuration phase<br>12h/12h light/dark cycle<br>Controlled temperature 11°C, pH 7.9
	Present study:<br>ku: 11.8 [10.5, 13.3]<br>ke: 2.6 [2.3, 2.9]	4.5	50 µg/L exposure concentration<br>24 h uptake and 48 h depuration phase<br>16h/8h light/dark cycle<br>Controlled temperature 11°C, pH 7.9

Compound	Rates	BCF	Experimental conditions
**Citalopram**	Arlos et al. 2020:<br>ku: 7.26 [6.34, 8.41]<br>ke: 0.14 [0.08, 0.21]	52.5	50 µg/L exposure concentration<br>48 h uptake and 48 h depuration phase<br>12h/12h light/dark cycle<br>Controlled temperature 11°C, pH 7.9
	Present study:<br>ku: 25.1 [21.1, 29.8]<br>ke: 1.2 [0.9, 1.5]	21.3	50 µg/L exposure concentration<br>24 h uptake and 48 h depuration phase<br>16h/8h light/dark cycle<br>Controlled temperature 11°C, pH 7.9
**Diclofenac**	Miller et al, 2016*:<br>ku: 0.273 ± 0.037<br>ke: 0.012 ± 0.008	22.8	10 µg/L exposure concentration<br>48 h uptake and 48 h depuration phase<br>12h/12h light/dark cycle<br>Controlled temp of 15°C average pH of 8.19 ± 0.05
	Arlos et al. 2020:<br>ku: 5.08 [3.23, 10.06]<br>ke: 1.04 [0.49, 1.87]	4.9	50 µg/L exposure concentration<br>48 h uptake and 48 h depuration phase<br>12h/12h light/dark cycle<br>Controlled temperature 11°C, pH 7.9
	Present study:<br>ku: 5.7 [4.0, 8.0]<br>ke: 2.8 [1.9, 3.8]	2.1	50 µg/L exposure concentration<br>24 h uptake and 48 h depuration phase<br>16h/8h light/dark cycle<br>Controlled temperature 11°C, pH 7.9
**Sulfamethoxazole**	Arlos et al. 2020:<br>ku: 0.47 [0.38, 0.61]<br>ke: 2.26 [1.82, 2.86]	0.2	50 µg/L exposure concentration<br>48 h uptake and 48 h depuration phase<br>12h/12h light/dark cycle<br>Controlled temperature 11°C, pH 7.9
	Present study:<br>ku: 0.7 [0.5, 0.9]<br>ke: 3.0 [2.3, 3.9]	0.2	50 µg/L exposure concentration<br>24 h uptake and 48 h depuration phase<br>16h/8h light/dark cycle<br>Controlled temperature 11°C, pH 7.9

Tab. S13: Comparison of the modelled toxicokinetic parameters (biotransformation model, G. pulex) of the present study with literature values.

**Biotransformation model *G. pulex***			
**Compound**	**Rates**	**BCF_{kin}**	**Experimental conditions**
**Azoxystrobin**	Fu et al. 2018:<br>ku: 43 [32, 53]<br>ke: 7.8 [5.5, 10]<br>km,1st 0.9 [0.7, 1.1]<br>km,2nd 3.1 [2.3, 4.1]	5.0	80 µg/L exposure concentration<br>24 h uptake and 120 h depuration phase<br>12h/12h light/dark cycle<br>Controlled temperature 11°C, pH 7.9
	Present study:<br>ku: 22.5 [12.4, 31,7]<br>ke: 2.3 [0.8, 4.0]<br>km,1st 0.67 [0.42, 3.65]<br>km,2nd 4.2 [2.7, 6.3]	7.5	50 µg/L exposure concentration<br>24 h uptake and 48 h depuration phase<br>16h/8h light/dark cycle<br>Controlled temperature 11°C, pH 7.9

Tab. S14: Comparison of the modelled toxicokinetic parameters (parent model, H. azteca) of the present study with literature values.

**Parent model *H. azteca***			
**Compound**	**Rates**	**BCF_{kin}**	**Experimental conditions**
**Terbutryn**	Kosfeld et al. 2020:<br>ku: 196.7 ± 24.6<br>ke: 9.1 ± 1.1	21.6	50 µg/L exposure concentration<br>48 h uptake and 48 h depuration phase<br>16h/8h light/dark cycle<br>Controlled temperature 23°C, pH 8.4
	Present study:<br>ku: 214.2 [157.4, 290.7]<br>ke: 8.5 [6.5, 11.3]	25.1	50 µg/L exposure concentration<br>24 h uptake and 72 h depuration phase<br>16h/8h light/dark cycle<br>Controlled temperature 21°C, pH 8.4

Tab. S15: Comparison of the modelled toxicokinetic parameters (biotransformation model, H. azteca) of the present study with literature values.

**Biotransformation model *H. azteca***			
**Compound**	**Rates**	**BCF_{kin}**	**Experimental conditions**
**Azoxystrobin**	Fu et al. 2018:<br>ku: 11 [9, 12]<br>ke: 0.13 [1E-04, 0.8]<br>km,1st 1.8 [1.4, 2.1]<br>km,2nd 8.6 [7.6, 11]	6.0	80 µg/L exposure concentration<br>24 h uptake and 120 h depuration phase<br>16h/8h light/dark cycle<br>Controlled temperature 23°C, pH 8.4
	Present study:<br>ku: 13.3 [11.9, 16.3]<br>ke: 0.0 [0.0, 0.5]<br>km,1st 1.39 [1.01, 1.65]<br>km,2nd 21.0 [18.0, 27.3]	9.4	50 µg/L exposure concentration<br>24 h uptake and 72 h depuration phase<br>16h/8h light/dark cycle<br>Controlled temperature 21°C, pH 8.4
**Terbutryn**	Kosfeld et al. 2020:<br>ku: 149.8 [130.7, 175,9]<br>ke: 1.09E-06 [1E-06, 1.203]<br>km,1st 6.05 [5.276, 7.104]<br>km,2nd 3.115 [2.209, 4.261]	24.8	50 µg/L exposure concentration<br>48 h uptake and 48 h depuration phase<br>16h/8h light/dark cycle<br>Controlled temperature 23°C, pH 8.4
	Present study:<br>ku: 170.4 [135.6, 217.9]<br>ke: 0.0 [0.0, 4.9]<br>km,1st 6.77 [3.42, 8.42]<br>km,2nd 2.2 [1.8, 2.8]	25.1	50 µg/L exposure concentration<br>24 h uptake and 72 h depuration phase<br>16h/8h light/dark cycle<br>Controlled temperature 21°C, pH 8.4

SI A14: Arrhenius temperature calculations

The Arrhenius fits and calculated Arrhenius temperatures (T_A) for the toxicokinetic rates are presented in Tab. S16 (parent model) and Tab. S17 (biotransformation model) as well as Fig. S33 and Fig. S34 (parent model) and Fig. S35 and Fig. S36 (biotransformation model). More detailed data on the fits are provided in SI B6.

*Tab. S16: Arrhenius temperatures of the toxicokinetic rates of the parent model. AT = atenolol, AZ = azoxystrobin, BTX = benzotriazole, CIT = citalopram, CMZ = carbamazepine, CY = cyprodinil, DCF = diclofenac, FLU = fluopyram, SFX = sulfamethoxazole, TEB = tebuconazole, TER = terbutryn, THI = thiacloprid. * = AZ and THI should be evaluated carefully as they showed obvious two-compartment kinetics.*

***Gammarus pulex* (parent model)**												
**Elimination rate**												
	**AT**	**AZ***	**BTX**	**CIT**	**CMZ**	**CY**	**DCF**	**FLU**	**SFX**	**TEB**	**TER**	**THI***
**T_A**	8040	10260	9660	6750	9320	4290	5600	7130	4040	7310	5480	7490
**SD**	1010	2540	2160	400	640	1080	2980	380	370	770	440	3100
**R^2**	0.97	0.89	0.91	0.99	0.99	0.89	0.64	0.99	0.98	0.98	0.99	0.74
**Uptake rate**												
**T_A**	9230	7770	6690	12490	9330	4910	6820	8590	6810	8110	6960	4100
**SD**	1610	1500	1000	1000	490	1090	580	630	1010	660	670	880
**R^2**	0.94	0.93	0.96	0.99	0.99	0.91	0.99	0.99	0.96	0.99	0.98	0.92
***Hyalella azteca* (parent model)**												
**Elimination rate**												
**T_A**	3340	12930	6900	1220	10130	9660	8310	9560	2460	9090	9760	8020
**SD**	10700	140	370	3770	1410	850	910	700	4060	470	1790	3810
**R^2**	0.05	1.00	0.99	0.05	0.96	0.98	0.98	0.99	0.15	0.99	0.94	0.69
**Uptake rate**												
**T_A**	8000	7480	3480	7160	12570	7620	9490	11710	3820	8810	9380	8410
**SD**	2160	630	690	1420	160	640	1700	450	8320	650	1140	550
**R^2**	0.87	0.99	0.93	0.93	1.00	0.99	0.94	1.00	0.10	0.99	0.97	0.99

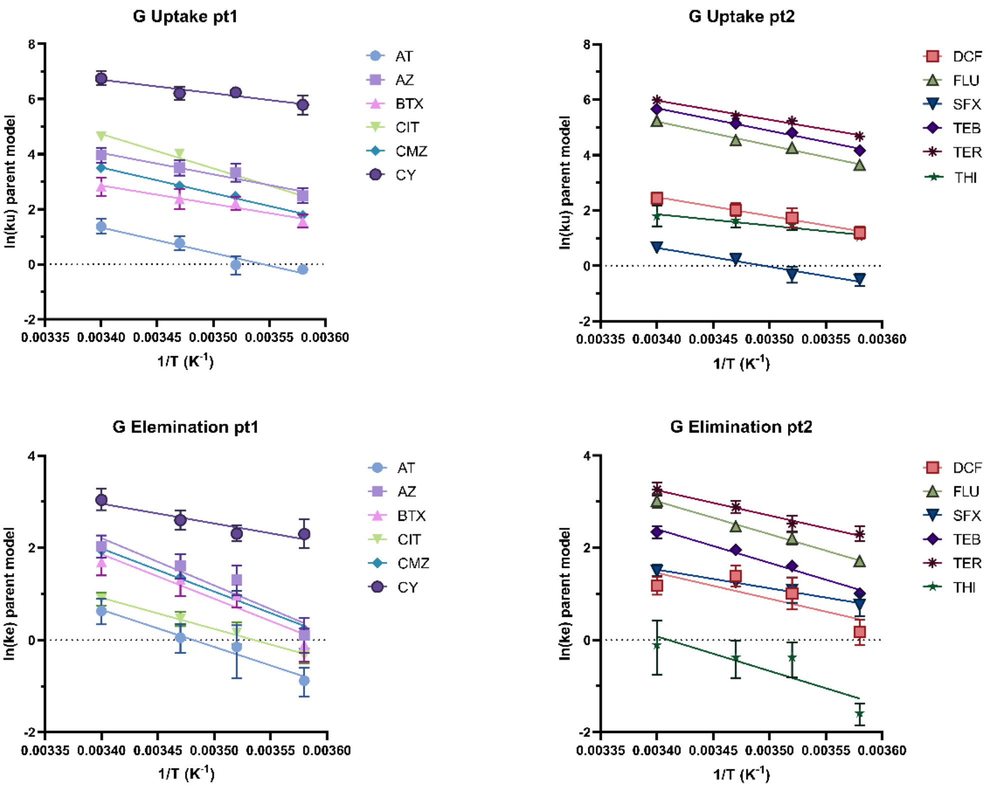

*Fig. S34: Arrhenius fits for the calculated toxicokinetic rates of the parent model in G. pulex (G). Presented as ln(k) ± 95% CI and a linear regression fit. AT = atenolol, AZ = azoxystrobin, BTX = benzotriazole, CIT = citalopram, CMZ = carbamazepine, CY = cyprodinil, DCF = diclofenac, FLU = fluopyram, SFX = sulfamethoxazole, TEB = tebuconazole, TER = terbutryn, THI = thiacloprid. * = AZ and THI should be evaluated carefully as they showed obvious two-compartment kinetics.*

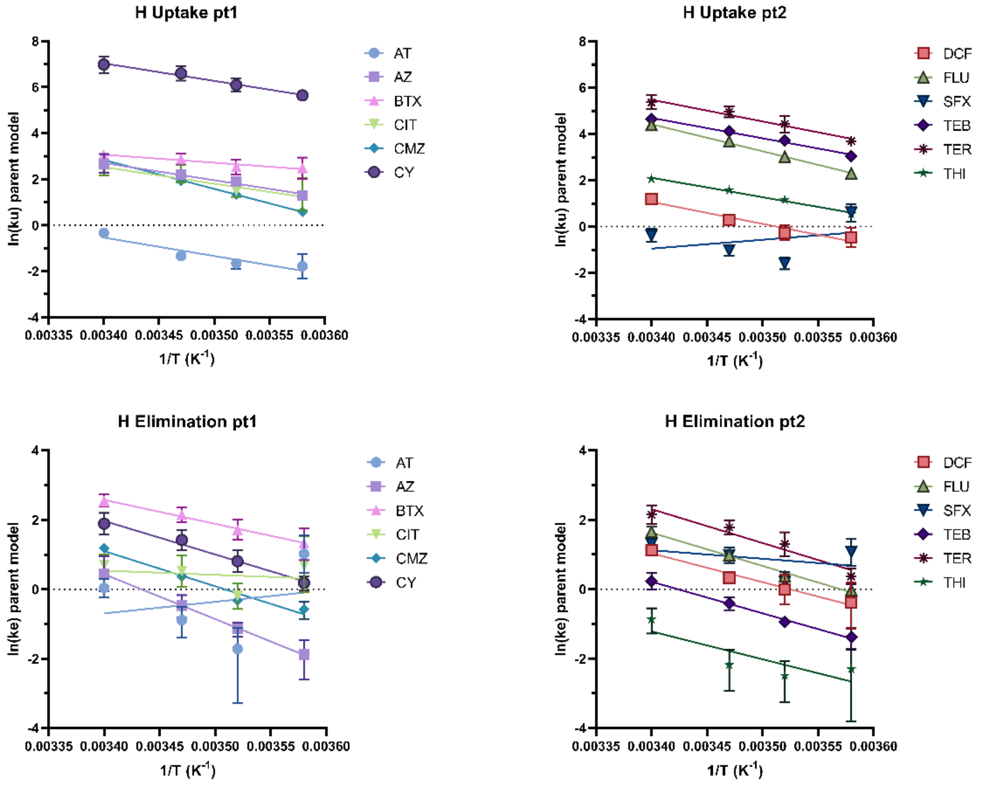

*Fig. S33: Arrhenius fits for the calculated toxicokinetic rates of the parent model in H. azteca (H). Presented as ln(k) ± 95% CI and a linear regression fit. AT = atenolol, AZ = azoxystrobin, BTX = benzotriazole, CIT = citalopram, CMZ = carbamazepine, CY = cyprodinil, DCF = diclofenac, FLU = fluopyram, SFX = sulfamethoxazole, TEB = tebuconazole, TER = terbutryn, THI = thiacloprid. * = AZ and THI should be evaluated carefully as they showed obvious two-compartment kinetics.*

*Tab. S17: Arrhenius temperatures of the toxicokinetic rates of the biotransformation model. AZ = azoxystrobin, CIT = citalopram, CY = cyprodinil, DCF = diclofenac, TEB = tebuconazole, TER = terbutryn. * = AZ should be evaluated carefully as it showed obvious two-compartment kinetics. n.a. = could not be calculated properly because the rates were close to zero.*

***Gammarus pulex* (biotransformation model)**																								
**Uptake rate**							**Elimination rate**							**Primary biotransformation rate**							**Secondary biotransformation rate**			
**ku**	**AZ***	**CY**	**CIT**	**DCF**	**TEB**	**TER**	**ke**	**AZ***	**CY**	**CIT**	**DCF**	**TEB**	**TER**	**km1**	**AZ***	**CY**	**CIT**	**DCF**	**TEB**	**TER**	**km2**	**AZ***	**CIT**	**TER**
**T_A**	8194	4701	12470	6630	7970	6560	**T_A**	6510	3950	6510	4740	7190	5220	**T_A**	6560	7600	7380	6750	4540	3470	**T_A**	6440		5450
**SD**	1210	1290	1620	900	690	670	**SD**	980	1210	110	4550	820	340	**SD**	650	3300	810	2410	880	2570	**SD**	900		390
**R^2**	0.96	0.87	0.97	0.96	0.99	0.98	**R^2**	0.98	0.84	1.00	0.35	0.97	0.99	**R^2**	0.98	0.73	0.98	0.80	0.93	0.48	**R^2**	0.96		0.99
***Hyalella azteca* (biotransformation model)**																								
**Uptake rate**							**Elimination rate**							**Primary biotransformation rate**							**Secondary biotransformation rate**			
**T_A**	7190	8640	6620			9340	**T_A**	n.a.	10450	n.a.			n.a.	**T_A**	14530	10240	11450			10580	**T_A**	8330	10480	11430
**SD**	1340	740	2160			1690	**SD**	n.a.	890	n.a.			n.a.	**SD**	700	1530	1120			2580	**SD**	1490	570	1000
**R^2**	0.94	0.99	0.82			0.94	**R^2**	n.a.	0.99	n.a.			n.a.	**R^2**	1.00	0.96	0.98			0.89	**R^2**	0.94	0.99	0.99

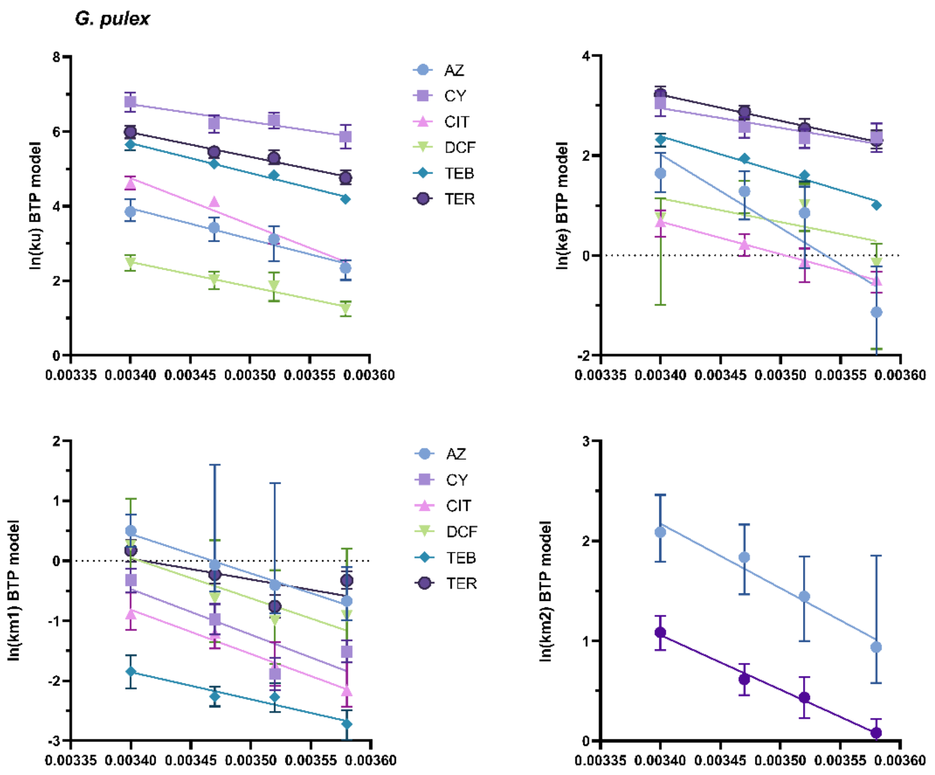

*Fig. S36: Arrhenius fits for the calculated toxicokinetic rates of the biotransformation model in G. pulex. Presented as ln(k) ± 95% CI and a linear regression fit. AZ = azoxystrobin, CIT = citalopram, CY = cyprodinil, DCF = diclofenac, TEB = tebuconazole, TER = terbutryn. * = AZ should be evaluated carefully as it showed obvious two-compartment kinetics. Please note the different y-axis scales.*

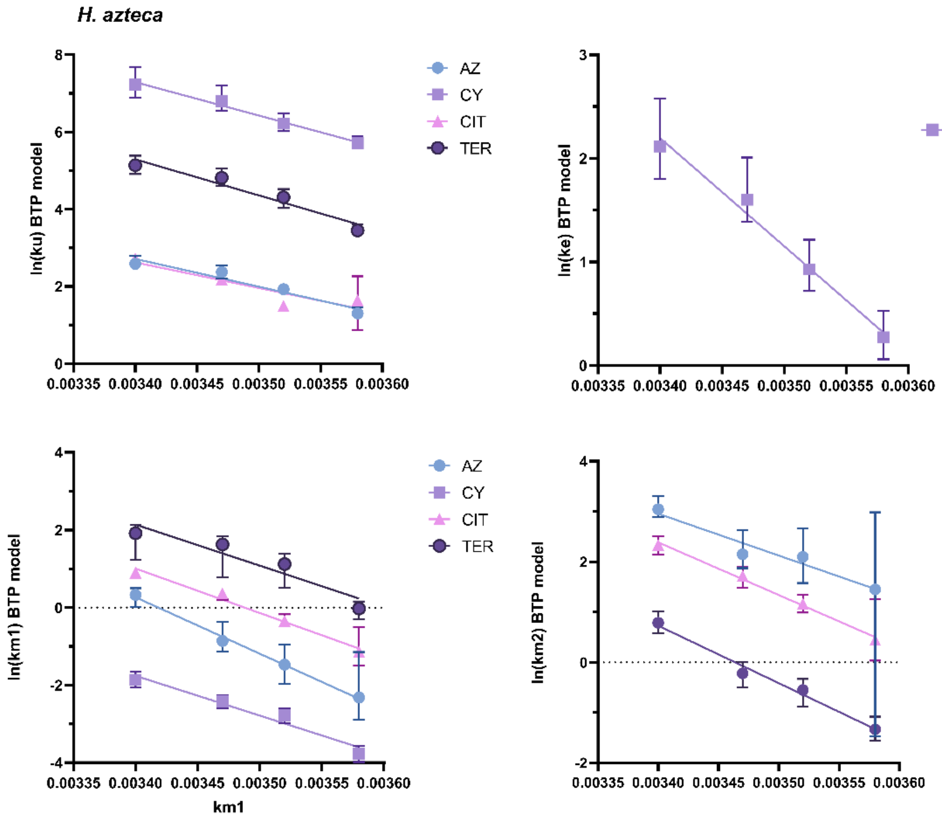

*Fig. S35: Arrhenius fits for the calculated toxicokinetic rates of the biotransformation model in H. azteca. Presented as ln(k) ± 95% CI and a linear regression fit. AZ = azoxystrobin, CIT = citalopram, CY = cyprodinil, DCF = diclofenac, TEB = tebuconazole, TER = terbutryn. * = AZ should be evaluated carefully as it showed obvious two-compartment kinetics. Please note the different y-axis scales.*

SI A15: Temperature dependence of the kinetic BCF

The relationships between the BCF and temperature are presented in Fig. S38 and Fig. S37. The model parameters are provided in SI B7. The slope of most temperature BCF_{kin} relationships was not significantly different from zero. Most slopes were driven by outliners at 6 or 21°C. The slope was significantly different for citalopram and fluopyram in *G. pulex* (positive relationship), and azoxystrobin and cyprodinil in *H. azteca* (negative relationship). However, the temperature effect on the BCF_{kin} of azoxystrobin was due to the two-compartment kinetics, which were not depictured properly by the applied one compartment model. The relationships were similar in the biotransformation model.

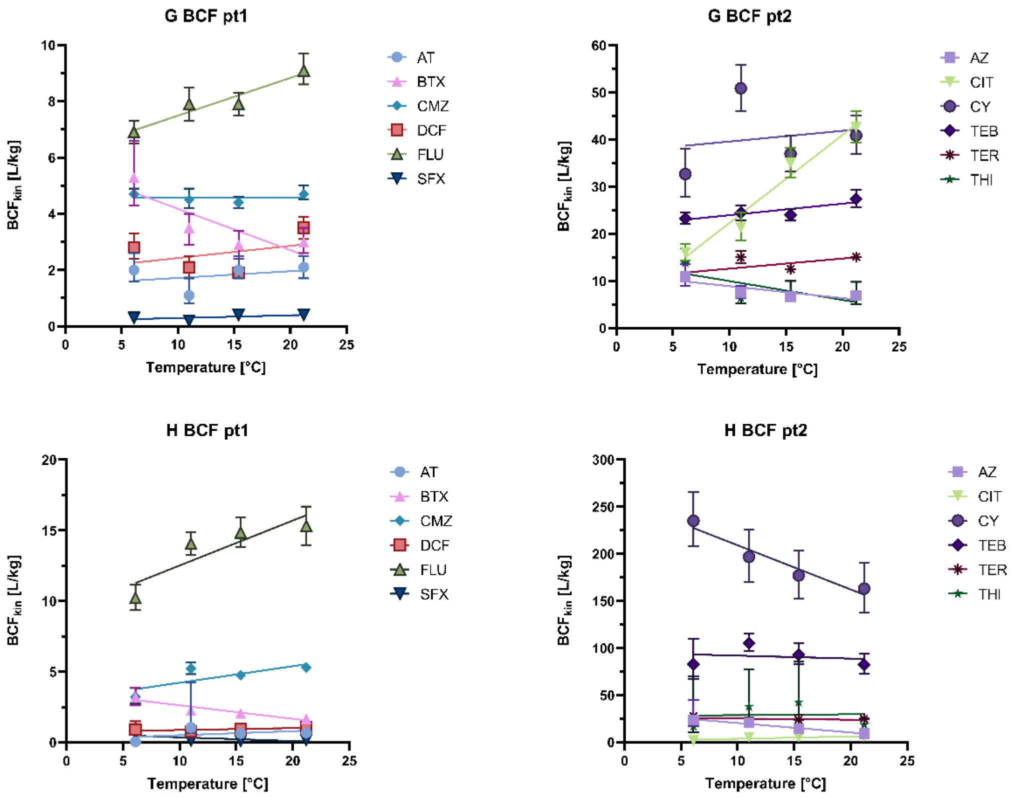

*Fig. S38: Relationship between temperature and BCF_{kin} of the parent model. Presented as BCF_{kin} ± 95% CI and a linear regression fit. G = G. pulex, H = H. azteca. AZ = azoxystrobin, CIT = citalopram, CY = cyprodinil, DCF = diclofenac, TEB = tebuconazole, TER = terbutryn. * = AZ should be evaluated carefully as it showed obvious two-compartment kinetics. Please note the different y-axis scales.*

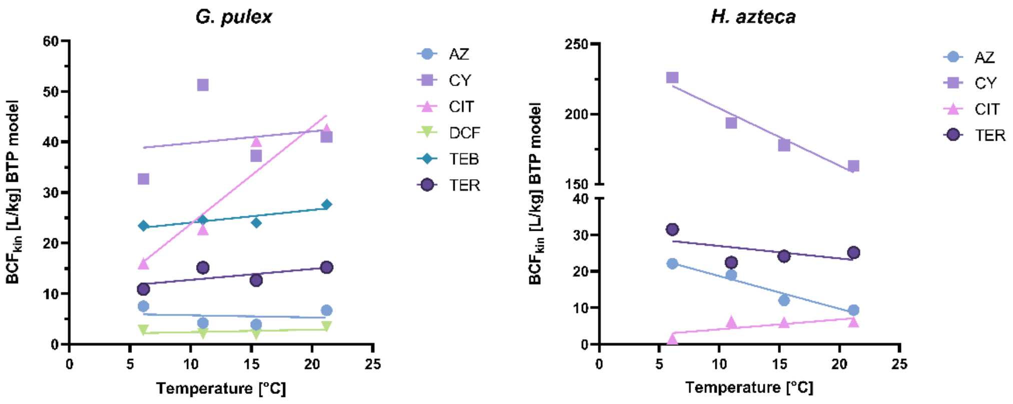

*Fig. S37: Relationship between temperature and BCF_{kin} of the biotransformation model. Presented as BCF_{kin} ± 95% CI and a linear regression fit. G = G. pulex, H = H. azteca. AZ = azoxystrobin, CIT = citalopram, CY = cyprodinil, DCF = diclofenac, TEB = tebuconazole, TER = terbutryn. * = AZ should be evaluated carefully as it showed obvious two-compartment kinetics. Please note the different y-axis scales.*

SI A16: Species differences in the kinetic bioconcentration factor

A linear fit of the BCF_{kin} in *G. pulex* and *H. azteca* is presented in Fig. S39 and resulted in a good fit if citalopram was excluded from the dataset. Compounds accumulated higher in G. pulex in the very low BCF_{kin} range (log BCF_{kin} < 0.65) and increased by a factor of 1.8 in *H. azteca*, resulting in higher BCFs in *H. azteca* in the log BCF_{kin} > 0.65 range.

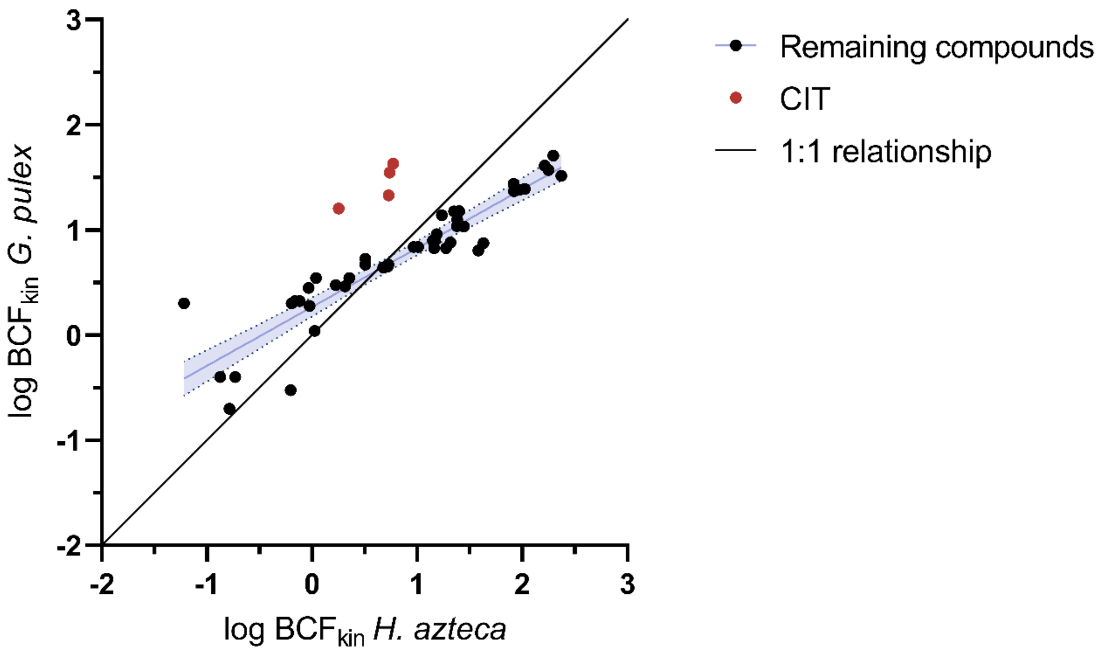

*Fig. S39: Linear regression of the log BCF_{kin} in G. pulex and H. azteca. Each compound is plotted at the four different temperatures. The black line indicates a 1:1 relationship. Citalopram (CIT, red) was excluded from the fit. R^2 = 0.85, slope significantly different from zero (p < 0.0001). Equation: Y = 0.56*X + 0.27.*

A comparison of the BCF_{kin} at same, and the different common test temperatures, of the two species is presented in Fig. S40. The qualitative differences in the BCF_{kin} did not change along the temperature comparisons.

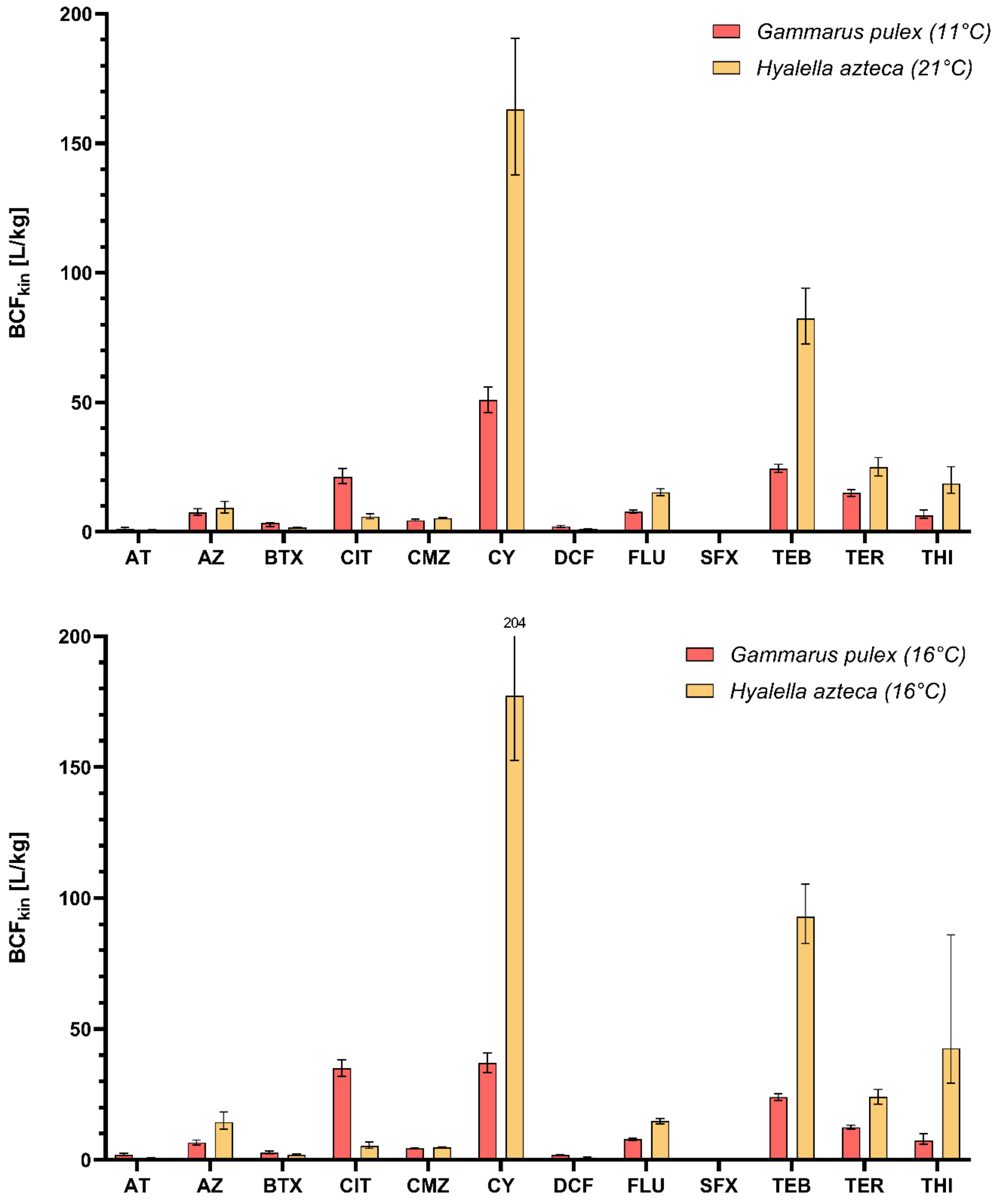

*Fig. S40: Comparison of the BCF_{kin} of G. pulex and H. azteca at their common test temperatures (above) and the same temperature (below). Presented as BCF_{kin} ± 95% CI. AT = atenolol, AZ = azoxystrobin, BTX = benzotriazole, CIT = citalopram, CMZ = carbamazepine, CY = cyprodinil, DCF = diclofenac, FLU = fluopyram, SFX = sulfamethoxazole, TEB = tebuconazole, TER = terbutryn, THI = thiacloprid. * = AZ and THI should be evaluated carefully as it showed obvious two-compartment kinetics.*

SI A17: Dead vs. alive gammarids

The modelled uptake an elimination rates were generally much higher in alive compared to dead gammarids (Fig. S41 and Fig. S42). However, the calculated BCF_{kin} was much more similar (Fig. S43).

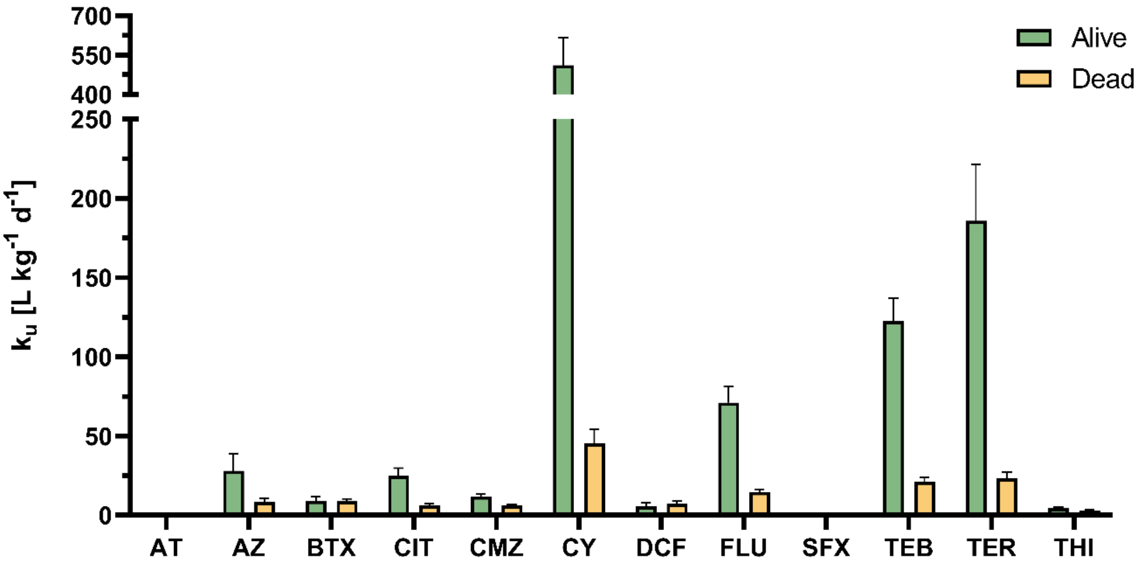

*Fig. S41: Uptake rate compared between dead and alive G. pulex. AT = atenolol, AZ = azoxystrobin, BTX = benzotriazole, CIT = citalopram, CMZ = carbamazepine, CY = cyprodinil, DCF = diclofenac, FLU = fluopyram, SFX = sulfamethoxazole, TEB = tebuconazole, TER = terbutryn, THI = thiacloprid. * = AZ and THI should be evaluated carefully as they showed obvious two-compartment kinetics.*

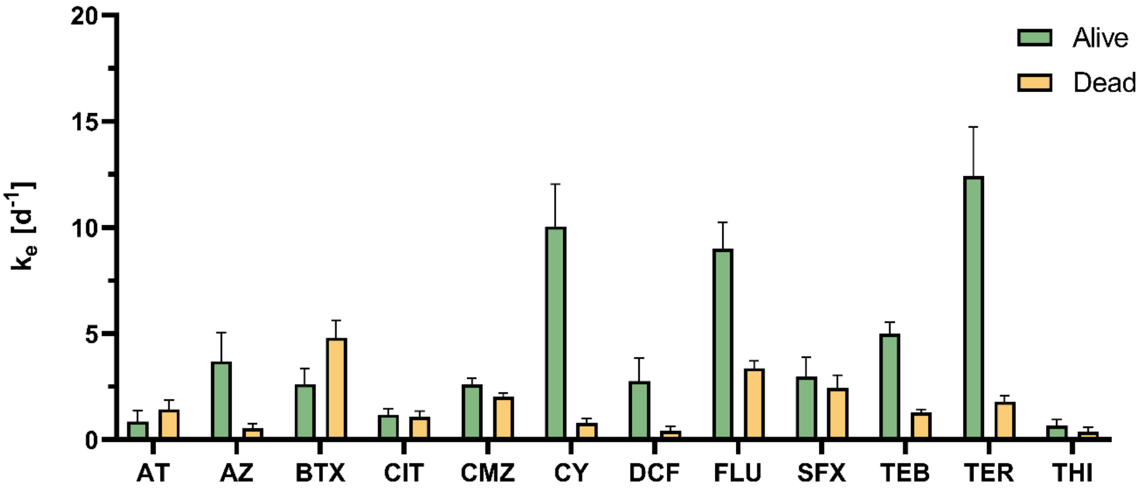

*Fig. S42: Elimination rate compared between dead and alive G. pulex. AT = atenolol, AZ = azoxystrobin, BTX = benzotriazole, CIT = citalopram, CMZ = carbamazepine, CY = cyprodinil, DCF = diclofenac, FLU = fluopyram, SFX = sulfamethoxazole, TEB = tebuconazole, TER = terbutryn, THI = thiacloprid. * = AZ and THI should be evaluated carefully as they showed obvious two-compartment kinetics.*

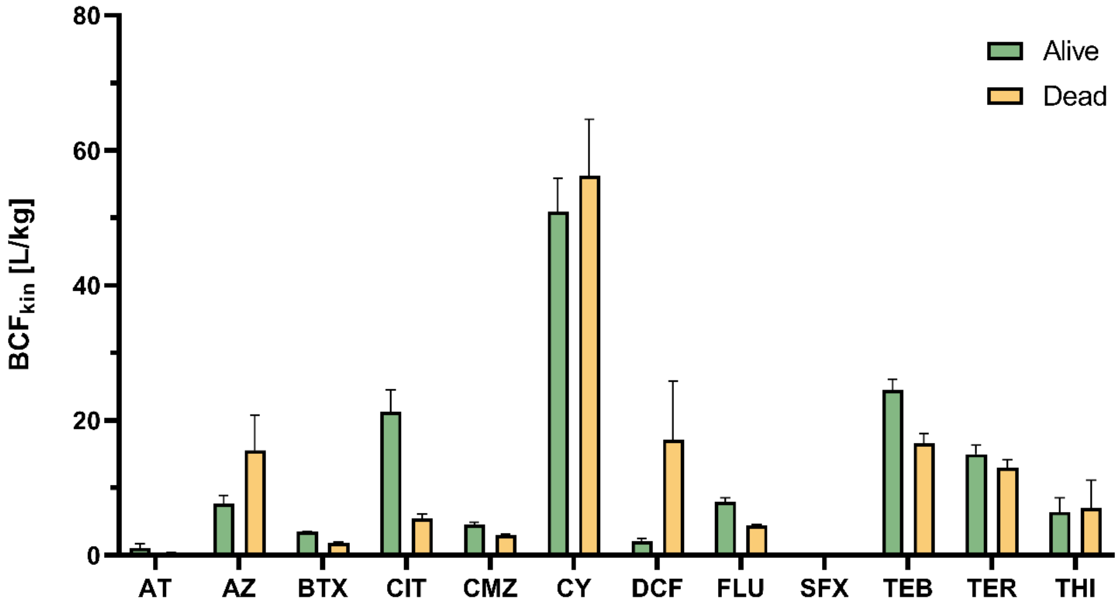

*Fig. S43: Kinetic bioconcentration factor compared between dead and alive G. pulex. AT = atenolol, AZ = azoxystrobin, BTX = benzotriazole, CIT = citalopram, CMZ = carbamazepine, CY = cyprodinil, DCF = diclofenac, FLU = fluopyram, SFX = sulfamethoxazole, TEB = tebuconazole, TER = terbutryn, THI = thiacloprid. * = AZ and THI should be evaluated carefully as they showed obvious two-compartment kinetics.*

SI A18: Arrhenius temperature calculations from Dai et al. (2021)

The calculated Arrhenius temperatures in *Enchytraeus albidus* from Dai et al. (2021) were 7530 ± 860 K (k_u) and 4940 ± 700 K (k_e). Corresponding fits are presented in Fig. S44. No AmP database (AmP, 2018) values for T_A were available for *E. albidus*, but for other earthworm species such as *Lumbricus terrestris* (6600) and *Eisenia andrei* (8000). The observed temperature dependent BCF_{kin} can be explained by the lower effect of temperature on the elimination rate compared to the uptake rate.

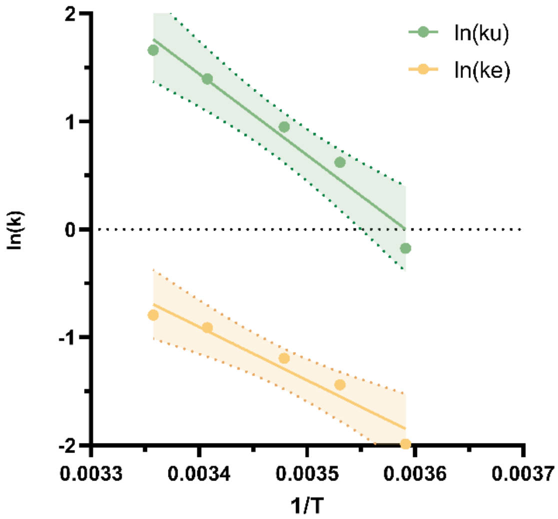

Fig. S44: Arrhenius relationships drawn from Dai et al. (2021). Presented with 95% CIs.

k_u:	*$R^2 = 0.96$*	*ln(k) = -7530 * 1/T + 7.0*
k_e:	*$R^2 = 0.94$*	*ln(k) = -4940 * 1/T + 15.9*

SI A19: Modelled simulations of different exposure and climate scenarios

The whole-body concentration the selected compounds in gammarids was modelled individually using the aqueous concentrations and temperature dependent toxicokinetic rates as input data. A python script was developed in SageMath 9.0 (https://www.sagemath.org/) where the differential equation is solved numerically using Heun's method (Ascher & Petzold, 1998) with 10,000 time-steps (Code and input data adapted from Lauper et al., 2021). The model start concentrations are obtained from the measured data of Lauper et al. (2021) and are close to zero.

The modelled internal concentration of fluopyram in *G. pulex* during an empirically determined short time exposure peak at the four different temperatures is presented in Fig. S45.

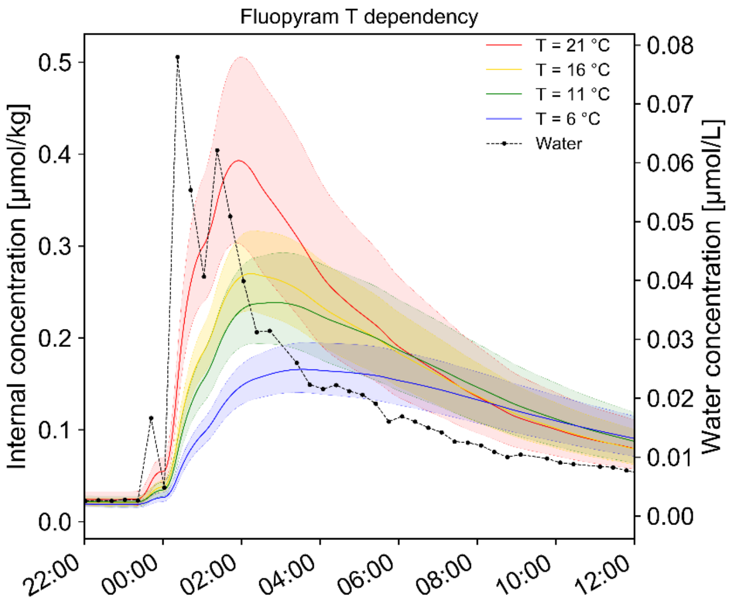

Fig. S45: Temperature dependent internal concentration of fluopyram in G. pulex during a monitored run off event (Lauper et al. 2021). Internal concentrations ± 95% CI were modelled using the toxicokinetic rates determined in the present study.

Additionally, modelled cyprodinil concentrations are presented in Fig. S46 (*G. pulex*) and Fig. S47 (*H. azteca*).

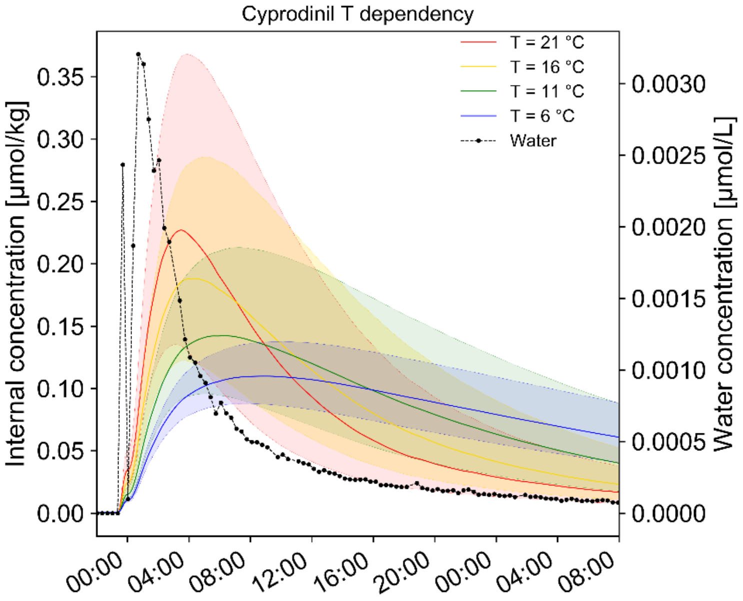

Fig. S47: Temperature dependent internal concentration of cyprodinil in H. azteca during a monitored run off event (Lauper et al. 2021). Internal concentrations ± 95% CI were modelled using the toxicokinetic rates determined in the present study.

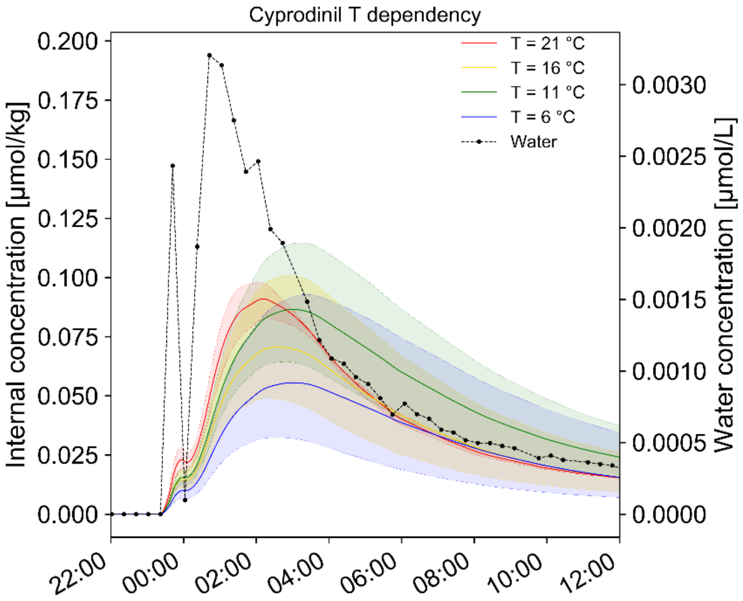

Fig. S46: Temperature dependent internal concentration of cyprodinil in G. pulex during a monitored run off event (Lauper et al. 2021). Internal concentrations ± 95% CI were modelled using the toxicokinetic rates determined in the present study.

SI A20: Effect of the exclusion of the 21°C *G. pulex* data set on calculated Arrhenius temperatures

The mortality of *G. pulex* in the exposure experiment at 21 °C was higher than expected (28%) based on previous experiments and exceeded a threshold of 20% mortality that is often applied to toxicokinetic experiments (i.e. according to OECD 305). Thus, the calculated Arrhenius temperatures based on a reduced data set, which excluded the 21 °C exposure scenario, were compared with Arrhenius temperatures of the full data set (Fig S48).

The comparison showed very little difference for the Arrhenius temperatures of the uptake rates, but slightly higher Arrhenius temperatures for the elimination rate of some compounds (azoxystrobin, benzotriazole, diclofenac and thiacloprid) if the 21 °C treatment was excluded. However, the fits of these four compounds had higher uncertainties, for instance because of two-compartment kinetics.

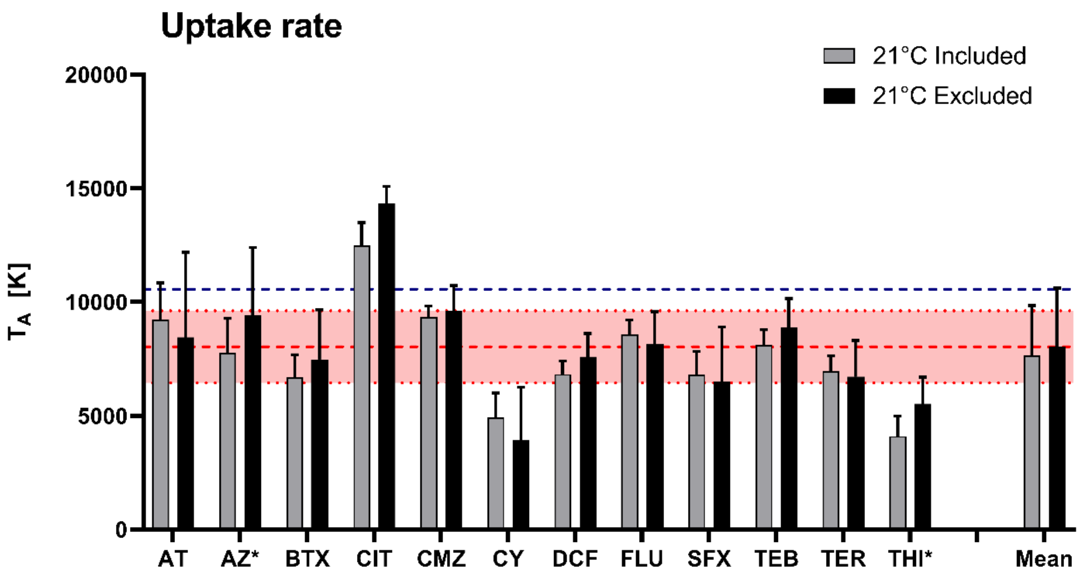

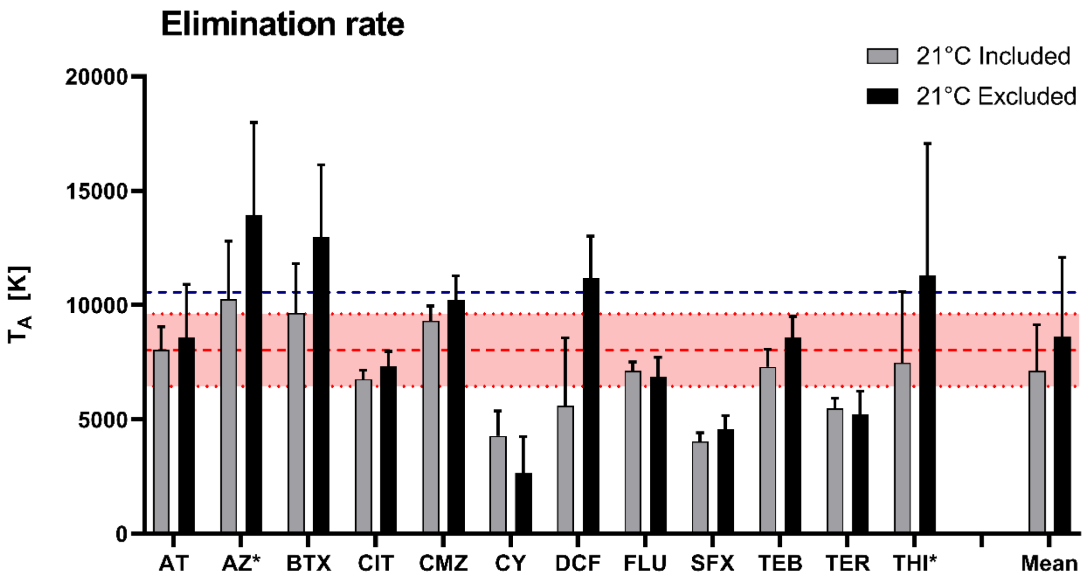

*Fig S48: Comparison of Arrhenius temperatures of the temperature dependent uptake (top) and elimination (bottom) rates in G. pulex (± SE) using the full data set (grey) and a reduced data set, which excluded the 21°C treatment (black). The dotted red lines represent the experimental determined physiological T_A (± SE) for H. azteca. The dark blue lines represent the T_A values from the AmP database. AT = atenolol, AZ = azoxystrobin, BTX = benzotriazole, CIT = citalopram, CMZ = carbamazepine, CY = cyprodinil, DCF = diclofenac, FLU = fluopyram, SFX = sulfamethoxazole, TEB = tebuconazole, TER = terbutryn, THI = thiacloprid. * = AZ and THI showed two-compartment kinetics (SI A10).*

References

AmP. (2018). *Add my Pet portal*. Add-My-Pet Database of Code, Data and DEB Model Parameters. https://www.bio.vu.nl/thb/deb/deblab/add_my_pet/index.html (accessed Feb 8, 2021).

Applied Biosystems. (2010). *Purifying extension products. In BigDye terminator v3.1 Cycle sequencing kit*.

Arlos, M. J., Schürz, F., Fu, Q., Lauper, B. B., Stamm, C., & Hollender, J. (2020). Coupling River Concentration Simulations with a Toxicokinetic Model Effectively Predicts the Internal Concentrations of Wastewater-Derived Micropollutants in Field Gammarids. *Environmental Science & Technology*, *54*(3), 1710–1719. https://doi.org/10.1021/acs.est.9b05736

Ascher, U. M., & Petzold, L. R. (1998). *Computer Methods for Ordinary Differential Equations and Differential-Algebraic Equations*. SIAM.

Ashauer, R., Hintermeister, A., Potthoff, E., & Escher, B. I. (2011). Acute toxicity of organic chemicals to *Gammarus pulex* correlates with sensitivity of *Daphnia magna* across most modes of action. Aquat Toxicol, 103(1–2), 38–45. https://doi.org/10.1016/j.aquatox.2011.02.002

Beketov, M. A., & Liess, M. (2008). Acute and delayed effects of the neonicotinoid insecticide thiacloprid on seven freshwater arthropods. Environmental Toxicology and Chemistry, 27(2), 461–470. https://doi.org/10.1897/07-322R.1

Borgmann, U. (1996). Systematic analysis of aqueous ion requirements of Hyalella azteca: A standard artificial medium including the essential bromide ion. *Archives of Environmental Contamination and Toxicology*, *30*(3), 356–363. https://doi.org/10.1007/BF00212294

Camp, A. A., & Buchwalter, D. B. (2016). Can't take the heat: Temperature-enhanced toxicity in the mayfly Isonychia bicolor exposed to the neonicotinoid insecticide imidacloprid. *Aquat Toxicol*, *178*, 49–57. https://doi.org/10.1016/j.aquatox.2016.07.011

Christensen, A. M., Faaborg-Andersen, S., Ingerslev, F., & Baun, A. (2007). Mixture and single-substance toxicity of selective serotonin reuptake inhibitors toward algae and crustaceans. Environmental Toxicology and Chemistry, 26(1), 85–91. https://doi.org/10.1897/06-219r.1

Dai, W., Slotsbo, S., van Gestel, C. A. M., & Holmstrup, M. (2021). Temperature-Dependent Toxicokinetics of Phenanthrene in *Enchytraeus albidus* (Oligochaeta). *Environmental Science & Technology*, *55*(3), 1876–1884. https://doi.org/10.1021/acs.est.0c06182

Dai, Y.-J., Ji, W.-W., Chen, T., Zhang, W.-J., Liu, Z.-H., Ge, F., & Yuan, S. (2010). Metabolism of the Neonicotinoid Insecticides Acetamiprid and Thiacloprid by the Yeast Rhodotorula mucilaginosa Strain IM-2. *Journal of Agricultural and Food Chemistry*, *58*(4), 2419–2425. https://doi.org/10.1021/jf903787s

Damalas, D. E., Bletsou, A. A., Agalou, A., Beis, D., & Thomaidis, N. S. (2018). Assessment of the Acute Toxicity, Uptake and Biotransformation Potential of Benzotriazoles in Zebrafish (*Danio rerio*) Larvae Combining HILIC- with RPLC-HRMS for High-Throughput Identification. *Environmental Science & Technology*, *52*(10), 6023–6031. https://doi.org/10.1021/acs.est.8b01327

Folmer, O., Black, M., Wr, H., Lutz, R., & Vrijenhoek, R. (1994). DNA primers for amplification of mitochondrial Cytochrome C oxidase subunit I from diverse metazoan invertebrates. *Molecular Marine Biology and Biotechnology*, *3*, 294–299.

Ford, K. A., & Casida, J. E. (2008). Comparative Metabolism and Pharmacokinetics of Seven Neonicotinoid Insecticides in Spinach. *Journal of Agricultural and Food Chemistry*, *56*(21), 10168–10175. https://doi.org/10.1021/jf8020909

Fu, Q., Fedrizzi, D., Kosfeld, V., Schlechtriem, C., Ganz, V., Derrer, S., Rentsch, D., & Hollender, J. (2020). Biotransformation Changes Bioaccumulation and Toxicity of Diclofenac in Aquatic Organisms. *Environmental Science & Technology*, *54*(7), 4400–4408. https://doi.org/10.1021/acs.est.9b07127

Fu, Q., Rösch, A., Fedrizzi, D., Vignet, C., & Hollender, J. (2018). Bioaccumulation, Biotransformation, and Synergistic Effects of Binary Fungicide Mixtures in *Hyalella azteca* and *Gammarus pulex*: How Different/Similar are the Two Species? *Environmental Science & Technology*, *52*(22), 13491–13500. https://doi.org/10.1021/acs.est.8b04057

Gergs, R., Rothhaupt, K.-O., & Behrmann-Godel, J. (2010). Characterisation of polymorphic microsatellite markers for the freshwater amphipod Gammarus pulex L. (Crustacea: Amphipoda). *Molecular Ecology Resources*, *10*(1), 232–236. https://doi.org/10.1111/j.1755-0998.2009.02796.x

Götz, A., Imhof, H. K., Geist, J., & Beggel, S. (2021). Moving Toward Standardized Toxicity Testing Procedures with Particulates by Dietary Exposure of Gammarids. *Environmental Toxicology and Chemistry*, *n/a*(n/a). https://doi.org/10.1002/etc.4990

Huntscha, S., Hofstetter, T. B., Schymanski, E. L., Spahr, S., & Hollender, J. (2014). Biotransformation of Benzotriazoles: Insights from Transformation Product Identification and Compound-Specific Isotope Analysis. *Environmental Science & Technology*, *48*(8), 4435–4443. https://doi.org/10.1021/es405694z

Janssen, E. M.-L., Choi, Y., & Luthy, R. G. (2012). Assessment of Nontoxic, Secondary Effects of Sorbent Amendment to Sediments on the Deposit-Feeding Organism Neanthes arenaceodentata. *Environmental Science & Technology*, *46*(7), 4134–4141. https://doi.org/10.1021/es204066g

Jeon, J., Kurth, D., & Hollender, J. (2013). Biotransformation pathways of biocides and pharmaceuticals in freshwater crustaceans based on structure elucidation of metabolites using high resolution mass spectrometry. *Chem Res Toxicol*, *26*(3), 313–324. https://doi.org/10.1021/tx300457f

Kiefer, K., Müller, A., Singer, H., & Hollender, J. (2019). New relevant pesticide transformation products in groundwater detected using target and suspect screening for agricultural and urban micropollutants with LC-HRMS. *Water Research*, *165*, 114972. https://doi.org/10.1016/j.watres.2019.114972

Kosfeld, V., Fu, Q., Ebersbach, I., Esser, D., Schauerte, A., Bischof, I., Hollender, J., & Schlechtriem, C. (2020). Comparison of Alternative Methods for Bioaccumulation Assessment: Scope and Limitations of In Vitro Depletion Assays with Rainbow Trout and Bioconcentration Tests in the Freshwater Amphipod Hyalella azteca. *Environmental Toxicology and Chemistry*, *39*(9), 1813–1825. https://doi.org/10.1002/etc.4791

Kretschmann, A., Ashauer, R., Preuss, T. G., Spaak, P., Escher, B. I., & Hollender, J. (2011). Toxicokinetic model describing bioconcentration and biotransformation of diazinon in Daphnia magna. *Environ Sci Technol*, *45*(11), 4995–5002. https://doi.org/10.1021/es104324v

Kumar, S., Nei, M., Dudley, J., & Tamura, K. (2008). MEGA: A biologist-centric software for evolutionary analysis of DNA and protein sequences. *Briefings in Bioinformatics*, *9*(4), 299–306. https://doi.org/10.1093/bib/bbn017

Lauper, B. B., Anthamatten, E., Raths, J., Arlos, M., & Hollender, J. (2021). Systematic Underestimation of Pesticide Burden for Invertebrates under Field Conditions: Comparing the Influence of Dietary Uptake and Aquatic Exposure Dynamics. *ACS Environmental Au*. https://doi.org/10.1021/acsenvironau.1c00023

Maloney, E. M., Taillebois, E., Gilles, N., Morrissey, C. A., Liber, K., Servent, D., & Thany, S. H. (2021). Binding properties to nicotinic acetylcholine receptors can explain differential toxicity of neonicotinoid insecticides in Chironomidae. *Aquatic Toxicology*, *230*, 105701. https://doi.org/10.1016/j.aquatox.2020.105701

Mathias, J. A. (1971). Energy Flow and Secondary Production of the Amphipods *Hyalella azteca* and *Crangonyx richmondensis occidentalis* in Marion Lake, British Columbia. *Journal of the Fisheries Research Board of Canada*, *28*(5), 711–726. https://doi.org/10.1139/f71-100

Meredith-Williams, M., Carter, L. J., Fussell, R., Raffaelli, D., Ashauer, R., & Boxall, A. B. (2012). Uptake and depuration of pharmaceuticals in aquatic invertebrates. *Environmental Pollution*, *165*, 250–258.

Miller, T. H., Bury, N. R., Owen, S. F., & Barron, L. P. (2017). Uptake, biotransformation and elimination of selected pharmaceuticals in a freshwater invertebrate measured using liquid chromatography tandem mass spectrometry. *Chemosphere*, *183*, 389–400. https://doi.org/10.1016/j.chemosphere.2017.05.083

Miller, T. H., McEneff, G. L., Stott, L. C., Owen, S. F., Bury, N. R., & Barron, L. P. (2016). Assessing the reliability of uptake and elimination kinetics modelling approaches for estimating bioconcentration factors in the freshwater invertebrate, Gammarus pulex. *Science of The Total Environment*, *547*, 396–404. https://doi.org/10.1016/j.scitotenv.2015.12.145

Naylor, C., Maltby, L., & Calow, P. (1989). Scope for growth in Gammarus pulex, a freshwater benthic detritivore. *Hydrobiologia*, *188*(1), 517–523. https://doi.org/10.1007/BF00027819

Pesticide Property Database PPDB (2020), https://sitem.herts.ac.uk/aeru/ppdb/en/atoz.htm, accessed 01.03.2020

Raths, J., Kuehr, S., & Schlechtriem, C. (2020). Bioconcentration, Metabolism, and Spatial Distribution of 14C-Labeled Laurate in the Freshwater Amphipod Hyalella azteca. *Environmental Toxicology and Chemistry*, *39*(2), 310–322. https://doi.org/10.1002/etc.4623

Rösch, A., Anliker, S., & Hollender, J. (2016). How Biotransformation Influences Toxicokinetics of Azole Fungicides in the Aquatic Invertebrate Gammarus pulex. *Environmental Science & Technology*, *50*(13), 7175–7188. https://doi.org/10.1021/acs.est.6b01301

Rösch, A., Gottardi, M., Vignet, C., Cedergreen, N., & Hollender, J. (2017). Mechanistic Understanding of the Synergistic Potential of Azole Fungicides in the Aquatic Invertebrate *Gammarus pulex*. Environmental Science & Technology, 51(21), 12784–12795. https://doi.org/10.1021/acs.est.7b03088

Sangkuhl, K., Klein, T. E., & Altman, R. B. (2011). PharmGKB summary: Citalopram pharmacokinetics pathway. *Pharmacogenetics and Genomics*, *21*(11), 769–772. https://doi.org/10.1097/FPC.0b013e328346063f

Sapp, M., Ertunç, T., Bringmann, I., Schäffer, A., & Schmidt, B. (2004). Characterization of the bound residues of the fungicide cyprodinil formed in plant cell suspension cultures of wheat. *Pest Management Science*, *60*(1), 65–74. https://doi.org/10.1002/ps.787

Schlechtriem, C., Kampe, S., Bruckert, H.-J., Bischof, I., Ebersbach, I., Kosfeld, V., Kotthoff, M., Schäfers, C., & L'Haridon, J. (2019). Bioconcentration studies with the freshwater amphipod Hyalella azteca: Are the results predictive of bioconcentration in fish? *Environmental Science and Pollution Research*, *26*(2), 1628–1641.

Schuelke, M. (2000). An economic method for the fluorescent labeling of PCR fragments. *Nature Biotechnology*, *18*(2), 233–234. https://doi.org/10.1038/72708

Seeland, A., Oetken, M., Kiss, A., Fries, E., & Oehlmann, J. (2012). Acute and chronic toxicity of benzotriazoles to aquatic organisms. Environmental Science and Pollution Research, 19(5), 1781–1790. https://doi.org/10.1007/s11356-011-0705-z

Smedes, F. (1999). Determination of total lipid using non-chlorinated solvents. *Analyst*, *124*(11), 1711–1718.

Švara, V., Norf, H., Luckenbach, T., Brack, W., & Michalski, S. G. (2019). Isolation and characterization of eleven novel microsatellite markers for fine-scale population genetic analyses of Gammarus pulex (Crustacea: Amphipoda). *Molecular Biology Reports*, *46*(6), 6609–6615. https://doi.org/10.1007/s11033-019-05077-y

Tain, L., Perrot-Minnot, M.-J., & Cézilly, F. (2006). Altered host behaviour and brain serotonergic activity caused by acanthocephalans: Evidence for specificity. *Proceedings of the Royal Society B: Biological Sciences*, *273*(1605), 3039–3045. https://doi.org/10.1098/rspb.2006.3618

Tamura, K., Peterson, D., Peterson, N., Stecher, G., Nei, M., & Kumar, S. (2011). MEGA5: Molecular evolutionary genetics analysis using maximum likelihood, evolutionary distance, and maximum parsimony methods. *Molecular Biology and Evolution*, *28*(10), 2731–2739. https://doi.org/10.1093/molbev/msr121

Vargas-Pérez, M., Egea González, F. J., & Garrido Frenich, A. (2020). Dissipation and residue determination of fluopyram and its metabolites in greenhouse crops. *Journal of the Science of Food and Agriculture*, *100*(13), 4826–4833. https://doi.org/10.1002/jsfa.10542

Westram, A. M., Jokela, J., & Keller, I. (2010). Isolation and characterization of ten polymorphic microsatellite markers for three cryptic *Gammarus fossarum* (Amphipoda) species. *Conservation Genetics Resources*, *2*(1), 401–404. https://doi.org/10.1007/s12686-010-9287-1

Zubrod, J. P., Englert, D., Wolfram, J., Wallace, D., Schnetzer, N., Baudy, P., Konschak, M., Schulz, R., & Bundschuh, M. (2015). Waterborne toxicity and diet-related effects of fungicides in the key leaf shredder *Gammarus fossarum* (Crustacea: Amphipoda). Aquat Toxicol, 169, 105–112. https://doi.org/10.1016/j.aquatox.2015.10.008
